# Supplementary material for: Decision Tree Analyses to Explore the Relevance of Multiple Sex/Gender Dimensions for the Exposure to Green Spaces: Results from the KORA INGER Study
Source: Int J Environ Res Public Health. 2022 Jun 18;19(12):7476. doi: 10.3390/ijerph19127476 (PMC9224469; doi:10.3390/ijerph19127476)

KORA Analyses

Load required packages and data

```
rm(list = ls())
setwd("D:/Projekte/INGER/KORA_Analysen")

library(rpart)
library(partykit)
```

```
## Warning: package 'partykit' was built under R version 4.0.3
## Loading required package: grid
## Loading required package: libcoin
## Warning: package 'libcoin' was built under R version 4.0.3
## Loading required package: mvtnorm
```

```
library(ggpubr)
```

```
## Warning: package 'ggpubr' was built under R version 4.0.3
## Loading required package: ggplot2
## Warning: package 'ggplot2' was built under R version 4.0.3
```

```
load("KORA_Analyses.Rda")

KORA = KORA_Analyses
```

Analysis of exposure variable "publicgreen"

Barplot

```
barplot(table(KORA$publicgreen), ylim=c(0,3500))
```

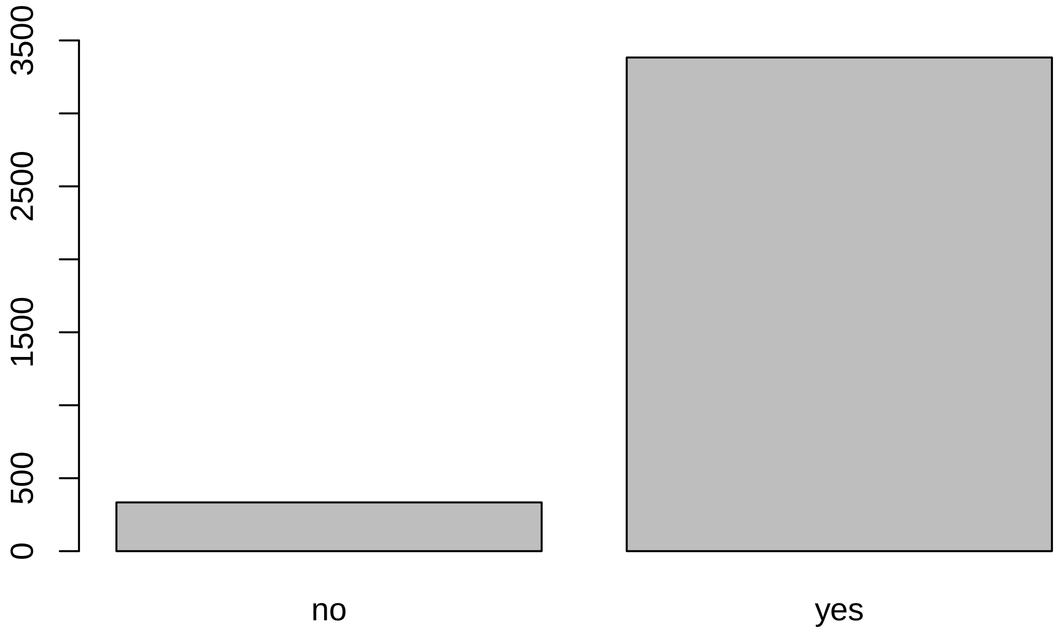

CART

```
set.seed(1234)

publicgreen_formula = publicgreen ~ SexAtBirth +  SGIdentity +   SGExpressionLooks +
  SGExpressionBehavior + SGExpressionSAGE +
  SGRolesFemMascuFeeling +   SGRolesFemMascuWish +
  SGRolesFemMascuChange +   SGRolesBothIncomeContribute + SGRolesMenMoneyWomenHouse +
  SGRolesGoodRelationshipWorkingMom + SGRolesWomenWorkChildSuffer +
  SGRolesWomenWorkFamilySuffer + SGRolesHousewifeFulfilling +
  SGRolesHousehusbandFulfilling + SGRolesSingleParentEqual +
  SGRolesSameSexEqual + CareActivitiesChildren +
  CareActivitiesSick + CareActivitiesCooking +
  CareActivitiesHousework + CareActivitiesErrands +
  CareActivitiesAdministrativeTasks +
  CareActivitiesHandicraft + CareActivitiesGardening +
  DiscriminationSocialPosition + DiscriminationAge +
  DiscriminationHeight + DiscriminationWeight +
  DiscriminationDisability + DiscriminationEthnicity +
  DiscriminationSG + DiscriminationSexualOrientation +
  DiscriminationAskedifParentsBornAbroad + SGRelationsIncome +
  SGRelationsEmployment + SGRelationsSchoolEducation +
  SGRelationsVocationalEducation + SGRelationsFamilySituation +
  SGRelationsUrbanisation
```

```r
tree_publicgreen = rpart(formula = publicgreen_formula, method = 'class',
                         data = KORA, parms = list(split='Gini'), cp = 0.001,
                         xval = 10, usesurrogate = 2,
                         minbucket = 50, maxdepth = 4)

printcp(tree_publicgreen)
```

```
##
## Classification tree:
## rpart(formula = publicgreen_formula, data = KORA, method = "class",
##     parms = list(split = "Gini"), cp = 0.001, xval = 10, usesurrogate = 2,
##     minbucket = 50, maxdepth = 4)
##
## Variables actually used in tree construction:
## character(0)
##
## Root node error: 334/3717 = 0.089857
##
## n=3717 (25 observations deleted due to missingness)
##
##   CP nsplit rel error xerror xstd
## 1  0      0         1      0    0
```

Pruning

```r
plot(as.party(tree_publicgreen),main='CART: publicgreen',
     ep_args = list(justmin = 15),gp = gpar(fontsize = 10),
     terminal_panel = node_barplot, tp_args = list(beside=TRUE))
```

CART: publicgreen

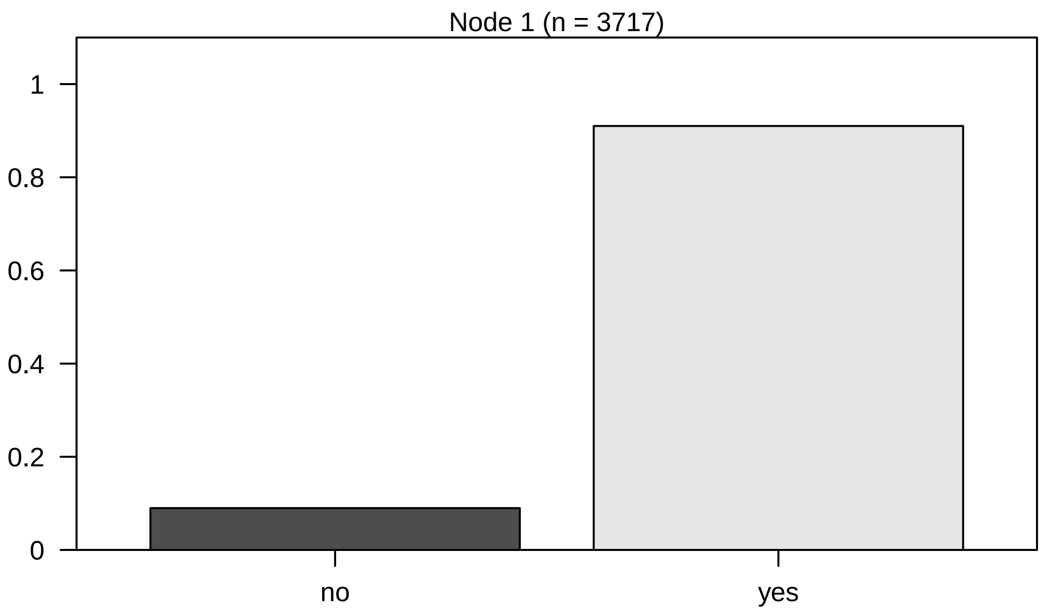

Summary

```
summary(tree_publicgreen)
```

```
## Call:
## rpart(formula = publicgreen_formula, data = KORA, method = "class",
##     parms = list(split = "Gini"), cp = 0.001, xval = 10, usesurrogate = 2,
##     minbucket = 50, maxdepth = 4)
##   n=3717 (25 observations deleted due to missingness)
##
##   CP nsplit rel error xerror xstd
## 1  0      0         1      0    0
##
## Node number 1: 3717 observations
##   predicted class=yes  expected loss=0.08985741  P(node) =1
##     class counts:   334  3383
##    probabilities: 0.090 0.910
```

CIT

```
set.seed(2345)

ctree_publicgreen = ctree(formula = publicgreen_formula,
                          data = KORA[!is.na(KORA$publicgreen),],
                          control = ctree_control(maxsurrogate = 5,
```

```
                                                  minbucket=50,
                                                  alpha = 0.05,
                                                  testtype = "Bonferroni",
                                                  maxdepth = 4))

plot(ctree_publicgreen,main='CIT: publicgreen',
     ep_args = list(justmin = 15),gp = gpar(fontsize = 10),
     terminal_panel = node_barplot, tp_args = list(beside=TRUE))
```

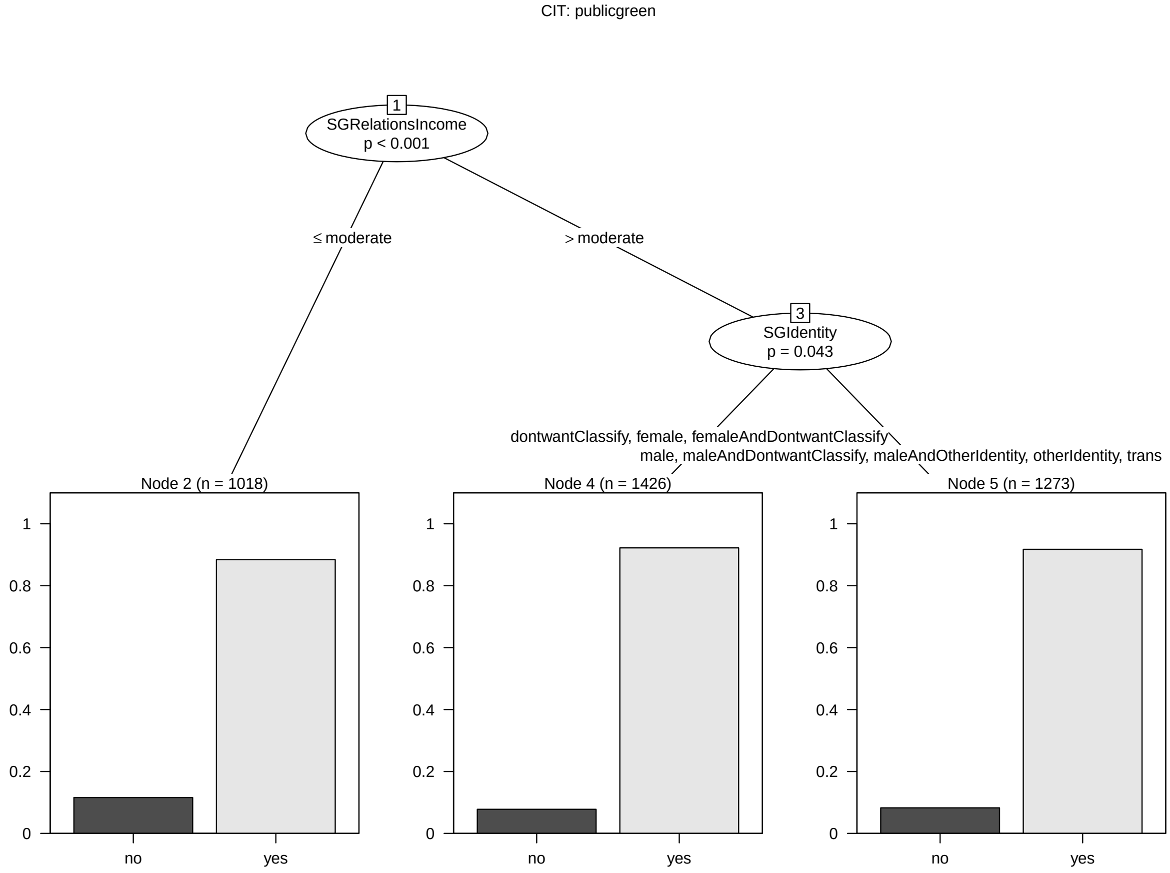

Variable importance via random forests

```
set.seed(3456)

cforest_publicgreen = cforest(formula = publicgreen_formula,
                              data=KORA[!is.na(KORA$publicgreen),],
                              control = ctree_control(testtype = "Univariate",
                                                      mincriterion = 0.95))

Variable_importance_publicgreen = varimp(cforest_publicgreen)
```

Variable importance plot

```
colors_publicgreen = c(rep('cornflowerblue',
                          times = sum(Variable_importance_publicgreen <
                                      abs(min(Variable_importance_publicgreen)))),
                      rep('blue3',
                          times = sum(Variable_importance_publicgreen >
                                      abs(min(Variable_importance_publicgreen)))))

par(mar=c(5,55,4,1)+.1)
barplot(sort(Variable_importance_publicgreen), space = 0.75,
        names.arg= rownames(Variable_importance_publicgreen),
        col = colors_publicgreen,
        horiz = TRUE, cex.names = 3.5, cex = 0.45,cex.axis=4, las = 1)
```

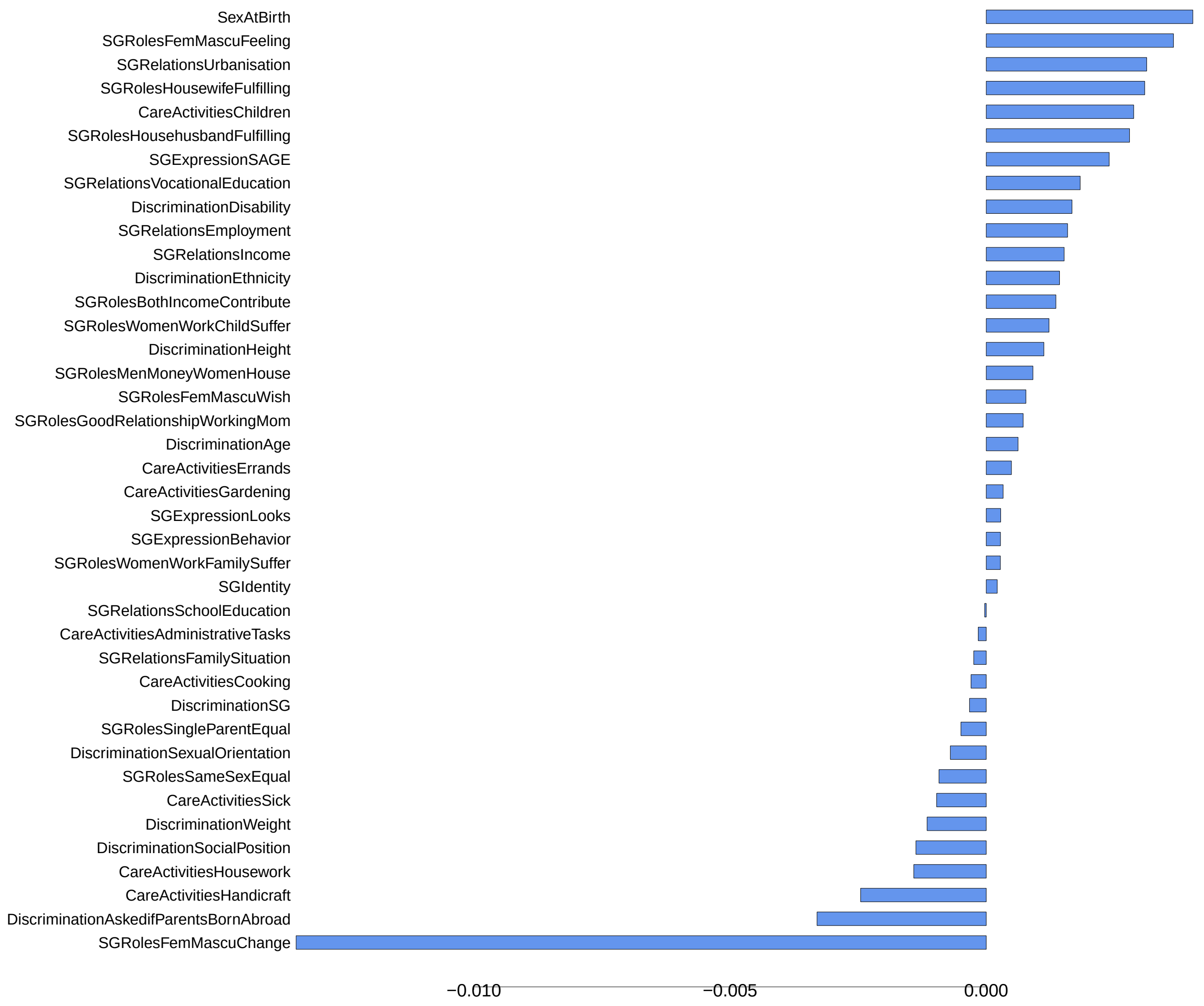

Analysis of exposure variable "qualitypublicgreen"

Barplot

```
barplot(table(KORA$qualitypublicgreen), ylim=c(0,2500))
```

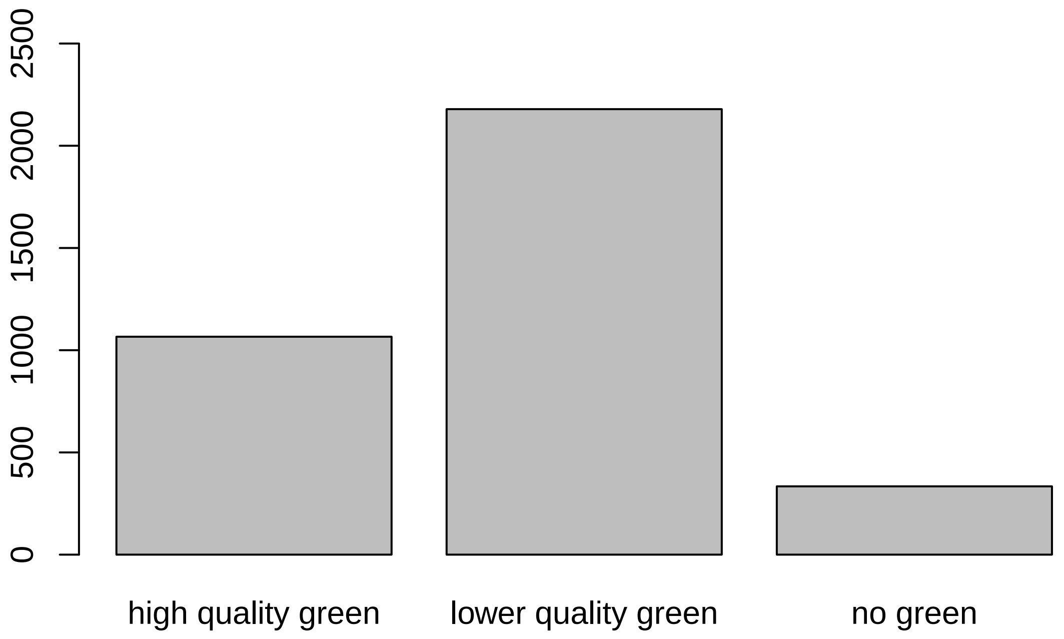

CART

```
set.seed(4567)

qualitypublicgreen_formula = qualitypublicgreen ~ SexAtBirth +  SGIdentity +
  SGExpressionLooks + SGExpressionBehavior + SGExpressionSAGE +
  SGRolesFemMascuFeeling +   SGRolesFemMascuWish +
  SGRolesFemMascuChange +   SGRolesBothIncomeContribute + SGRolesMenMoneyWomenHouse +
  SGRolesGoodRelationshipWorkingMom + SGRolesWomenWorkChildSuffer +
  SGRolesWomenWorkFamilySuffer + SGRolesHousewifeFulfilling +
  SGRolesHousehusbandFulfilling + SGRolesSingleParentEqual +
  SGRolesSameSexEqual + CareActivitiesChildren +
  CareActivitiesSick + CareActivitiesCooking +
  CareActivitiesHousework + CareActivitiesErrands +
  CareActivitiesAdministrativeTasks +
  CareActivitiesHandicraft + CareActivitiesGardening +
  DiscriminationSocialPosition + DiscriminationAge +
  DiscriminationHeight + DiscriminationWeight +
  DiscriminationDisability + DiscriminationEthnicity +
```

```
  DiscriminationSG + DiscriminationSexualOrientation +
  DiscriminationAskedifParentsBornAbroad + SGRelationsIncome +
  SGRelationsEmployment + SGRelationsSchoolEducation +
  SGRelationsVocationalEducation + SGRelationsFamilySituation +
  SGRelationsUrbanisation

tree_qualitypublicgreen = rpart(formula = qualitypublicgreen_formula, method = 'class',
                                data = KORA, parms = list(split='Gini'), cp = 0.001,
                                xval = 10, usesurrogate = 2,
                                minbucket = 50, maxdepth = 4)
printcp(tree_qualitypublicgreen)
```

```
##
## Classification tree:
## rpart(formula = qualitypublicgreen_formula, data = KORA, method = "class",
##     parms = list(split = "Gini"), cp = 0.001, xval = 10, usesurrogate = 2,
##     minbucket = 50, maxdepth = 4)
##
## Variables actually used in tree construction:
## [1] DiscriminationAge          SGRelationsIncome
## [3] SGRolesHousewifeFulfilling
##
## Root node error: 1400/3579 = 0.39117
##
## n=3579 (163 observations deleted due to missingness)
##
##          CP nsplit rel error  xerror     xstd
## 1 0.0092857      0   1.00000 1.00000 0.020854
## 2 0.0057143      2   0.98143 0.99500 0.020835
## 3 0.0010000      3   0.97571 0.98714 0.020805
```

Pruning

```
tree_qualitypublicgreen = prune(tree_qualitypublicgreen,cp=0.005)

plot(as.party(tree_qualitypublicgreen),main='CART: qualitypublicgreen',
     ep_args = list(justmin = 15),gp = gpar(fontsize = 10))
```

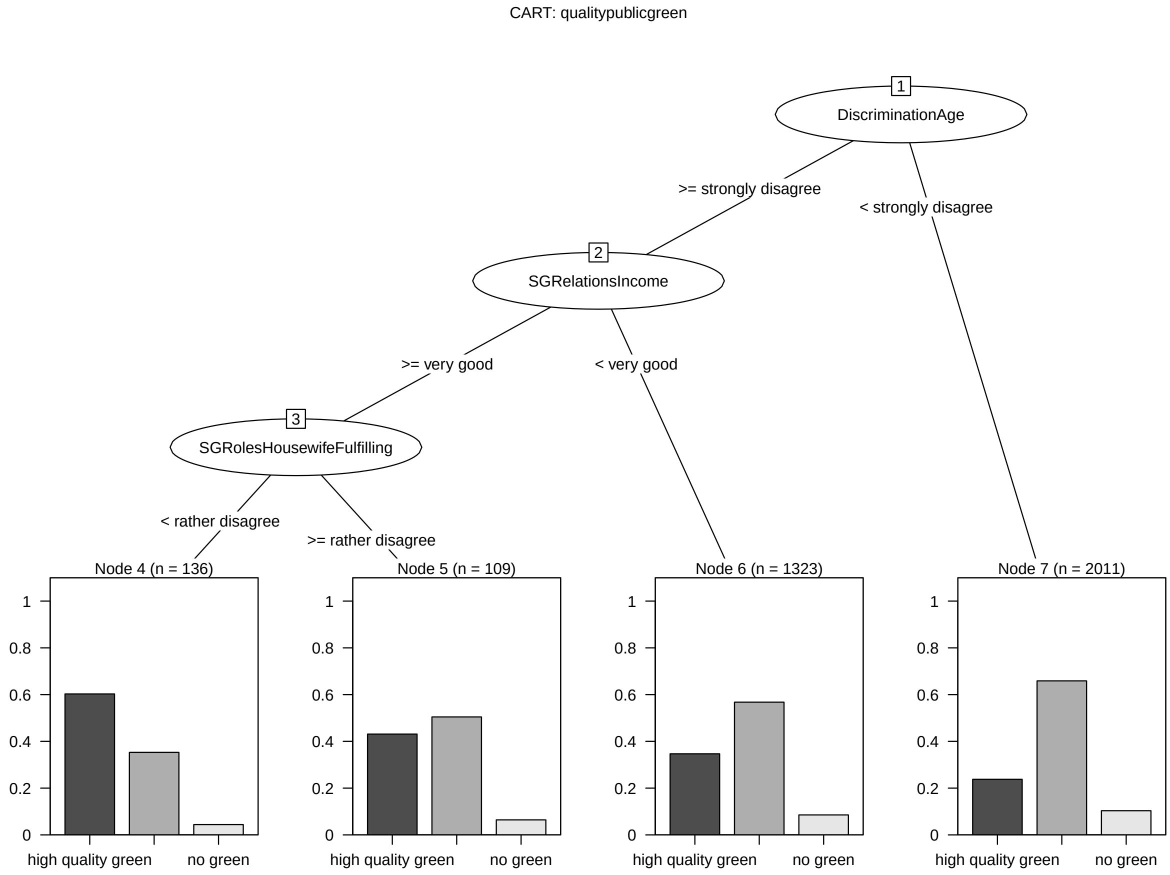

Summary

```r
summary(tree_qualitypublicgreen)
```

```
## Call:
## rpart(formula = qualitypublicgreen_formula, data = KORA, method = "class",
##     parms = list(split = "Gini"), cp = 0.001, xval = 10, usesurrogate = 2,
##     minbucket = 50, maxdepth = 4)
##   n=3579 (163 observations deleted due to missingness)
##
##            CP nsplit rel error    xerror       xstd
## 1 0.009285714      0 1.0000000 1.0000000 0.02085373
## 2 0.005714286      2 0.9814286 0.9950000 0.02083492
## 3 0.001000000      3 0.9757143 0.9871429 0.02080464
##
## Variable importance
##             DiscriminationAge  DiscriminationSocialPosition
##                            31                            18
##             SGRelationsIncome          DiscriminationHeight
##                            12                            12
##          DiscriminationWeight      DiscriminationDisability
##                            10                             7
##    SGRolesHousewifeFulfilling              DiscriminationSG
##                             4                             2
```

```
## SGRolesHousehusbandFulfilling     SGRolesMenMoneyWomenHouse
##                             2                             1
##
## Node number 1: 3579 observations,    complexity param=0.009285714
##   predicted class=lower quality green  expected loss=0.3911707  P(node) =1
##     class counts:  1066  2179   334
##    probabilities: 0.298 0.609 0.093
##   left son=2 (1568 obs) right son=3 (2011 obs)
##   Primary splits:
##       DiscriminationAge             splits as  RRRRL, improve=28.78425, (30 missing)
##       DiscriminationSocialPosition splits as  RRRRL, improve=26.77156, (40 missing)
##       SGRelationsIncome             splits as  RRRL,  improve=23.03132, (25 missing)
##       DiscriminationHeight          splits as  RRRRL, improve=15.70252, (29 missing)
##       DiscriminationWeight          splits as  RRRRL, improve=15.56627, (32 missing)
##   Surrogate splits:
##       DiscriminationSocialPosition splits as  RRRRL, agree=0.812, adj=0.574, (5 split)
##       DiscriminationHeight          splits as  RRRRL, agree=0.722, adj=0.371, (1 split)
##       DiscriminationWeight          splits as  RRRRL, agree=0.707, adj=0.336, (0 split)
##       DiscriminationDisability      splits as  RRRRL, agree=0.656, adj=0.220, (1 split)
##       DiscriminationSG              splits as  RRRRL, agree=0.589, adj=0.067, (0 split)
##
## Node number 2: 1568 observations,    complexity param=0.009285714
##   predicted class=lower quality green  expected loss=0.4553571  P(node) =0.4381112
##     class counts:   588   854   126
##    probabilities: 0.375 0.545 0.080
##   left son=4 (245 obs) right son=5 (1323 obs)
##   Primary splits:
##       SGRelationsIncome             splits as  RRRL,   improve=11.559190, (8 missing)
##       DiscriminationSocialPosition splits as  RRRRL,  improve= 5.298706, (4 missing)
##       CareActivitiesGardening       splits as  LLLRLL, improve= 4.211614, (13 missing)
##       SGRelationsUrbanisation       splits as  RLL,    improve= 3.228508, (179 missing)
##       SGRolesWomenWorkFamilySuffer splits as  RLLLL,  improve= 2.870207, (18 missing)
##
## Node number 3: 2011 observations
##   predicted class=lower quality green  expected loss=0.3411238  P(node) =0.5618888
##     class counts:   478  1325   208
##    probabilities: 0.238 0.659 0.103
##
## Node number 4: 245 observations,    complexity param=0.005714286
##   predicted class=high quality green   expected loss=0.4734694  P(node) =0.06845488
##     class counts:   129   103    13
##    probabilities: 0.527 0.420 0.053
##   left son=8 (136 obs) right son=9 (109 obs)
##   Primary splits:
##       SGRolesHousewifeFulfilling    splits as  LLLRR,   improve=3.406649, (4 missing)
##       SGRolesHousehusbandFulfilling splits as  LLLRR,   improve=2.203096, (8 missing)
##       SGRolesSameSexEqual           splits as  LRRRR,   improve=2.143286, (2 missing)
##       SGRolesWomenWorkChildSuffer   splits as  RRLLL,   improve=1.701268, (2 missing)
##       SGRolesFemMascuFeeling        splits as  LLLLLRR, improve=1.496083, (4 missing)
##   Surrogate splits:
##       SGRolesHousehusbandFulfilling splits as  LLLRR, agree=0.797, adj=0.542, (2 split)
##       SGRolesMenMoneyWomenHouse     splits as  LLLLR, agree=0.631, adj=0.168, (2 split)
##       SGRolesBothIncomeContribute   splits as  RLLLL, agree=0.614, adj=0.131, (0 split)
##       CareActivitiesHandicraft      splits as  LLLRR, agree=0.614, adj=0.131, (0 split)
```

```
##       SGRolesWomenWorkFamilySuffer  splits as  LLLLR, agree=0.610, adj=0.121, (0 split)
##
## Node number 5: 1323 observations
##   predicted class=lower quality green  expected loss=0.4323507  P(node) =0.3696563
##     class counts:   459   751   113
##    probabilities: 0.347 0.568 0.085
##
## Node number 8: 136 observations
##   predicted class=high quality green   expected loss=0.3970588  P(node) =0.03799944
##     class counts:    82    48     6
##    probabilities: 0.603 0.353 0.044
##
## Node number 9: 109 observations
##   predicted class=lower quality green  expected loss=0.4954128  P(node) =0.03045543
##     class counts:    47    55     7
##    probabilities: 0.431 0.505 0.064
```

CIT

```r
set.seed(5678)

ctree_qualitypublicgreen = ctree(formula = qualitypublicgreen_formula,
                                 data = KORA[!is.na(KORA$qualitypublicgreen),],
                                 control = ctree_control(maxsurrogate = 5,
                                                         minbucket=50,
                                                         alpha = 0.05,
                                                         testtype = "Bonferroni",
                                                         maxdepth = 4))

plot(ctree_qualitypublicgreen,main='CIT: qualitypublicgreen',
     ep_args = list(justmin = 15),gp = gpar(fontsize = 10))
```

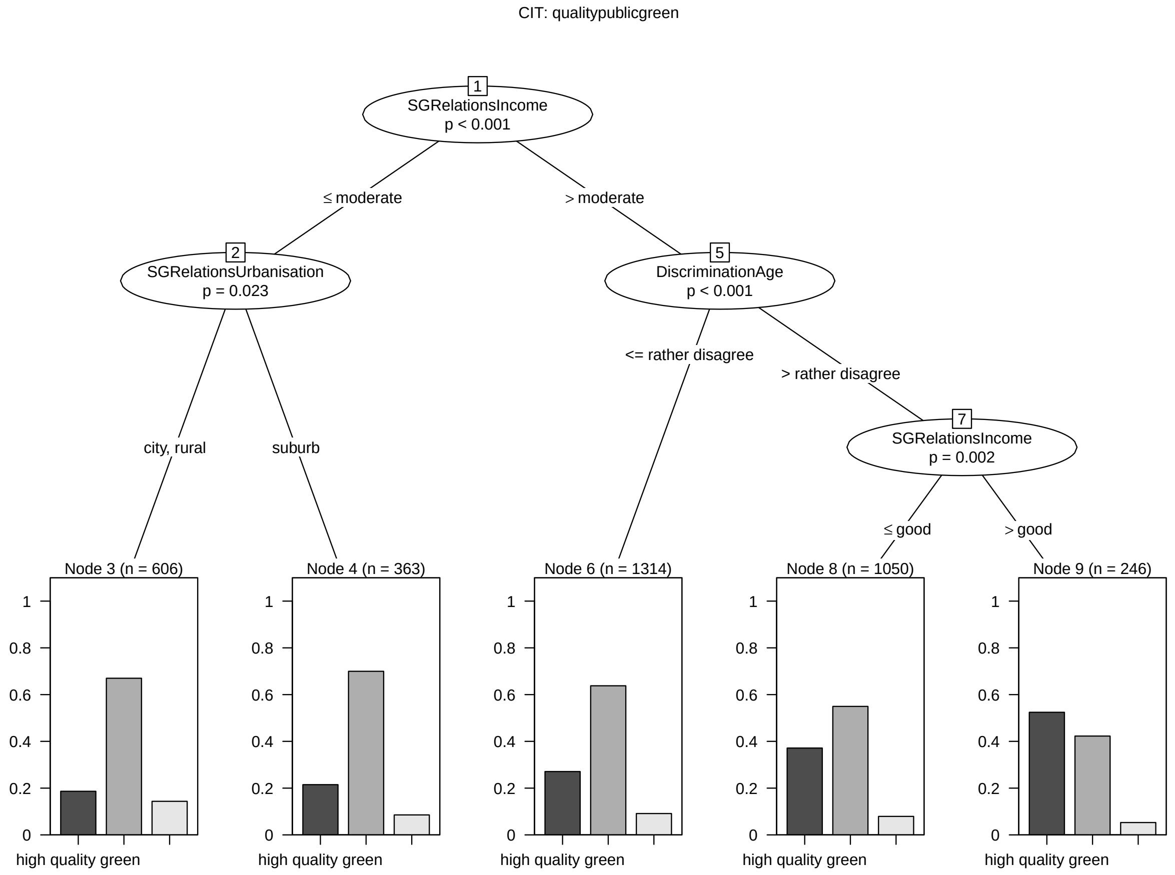

Variable importance via random forests

```
set.seed(6789)

cforest_qualitypublicgreen = cforest(formula = qualitypublicgreen_formula,
                                     data=KORA[!is.na(KORA$qualitypublicgreen),],
                                     control = ctree_control(testtype = "Univariate",
                                                             mincriterion = 0.95))

Variable_importance_qualitypublicgreen = varimp(cforest_qualitypublicgreen)
```

Variable importance plot

```
colors_qualitypublicgreen = c(rep('cornflowerblue',
                                  times = sum(Variable_importance_qualitypublicgreen <
                                        abs(min(Variable_importance_qualitypublicgreen)))),
                              rep('blue3',
                                  times = sum(Variable_importance_qualitypublicgreen >
                                        abs(min(Variable_importance_qualitypublicgreen)))))

par(mar=c(5,55,4,1)+.1)
barplot(sort(Variable_importance_qualitypublicgreen), space = 0.75,
        col = colors_qualitypublicgreen,
```

```
    names.arg= rownames(Variable_importance_qualitypublicgreen),
    horiz = TRUE, cex.names = 3.5, cex = 0.45,cex.axis=4, las = 1)
```

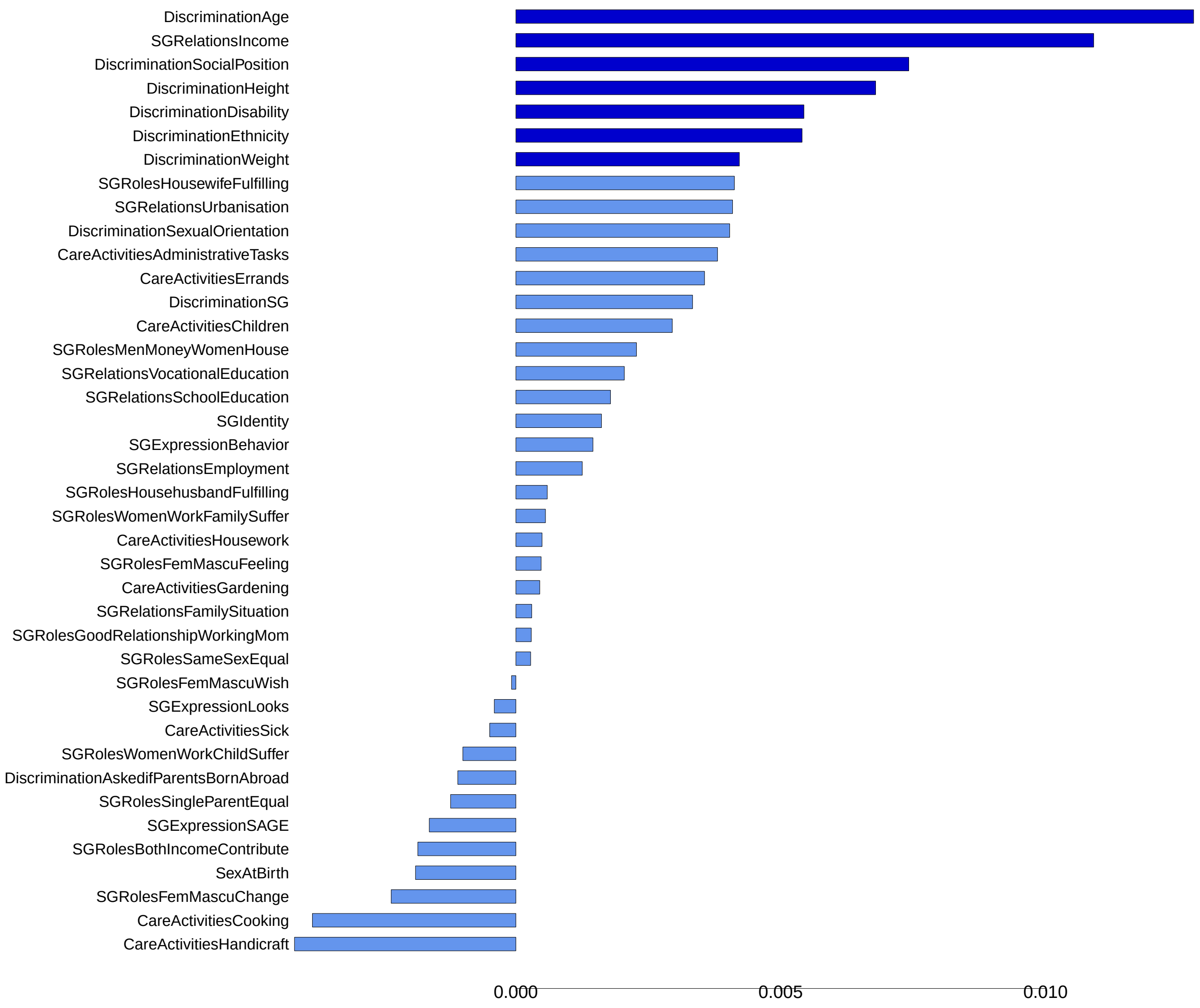

Analysis of exposure variable "greenness"

Barplot

```
barplot(table(KORA$greenness), ylim=c(0,3000))
```

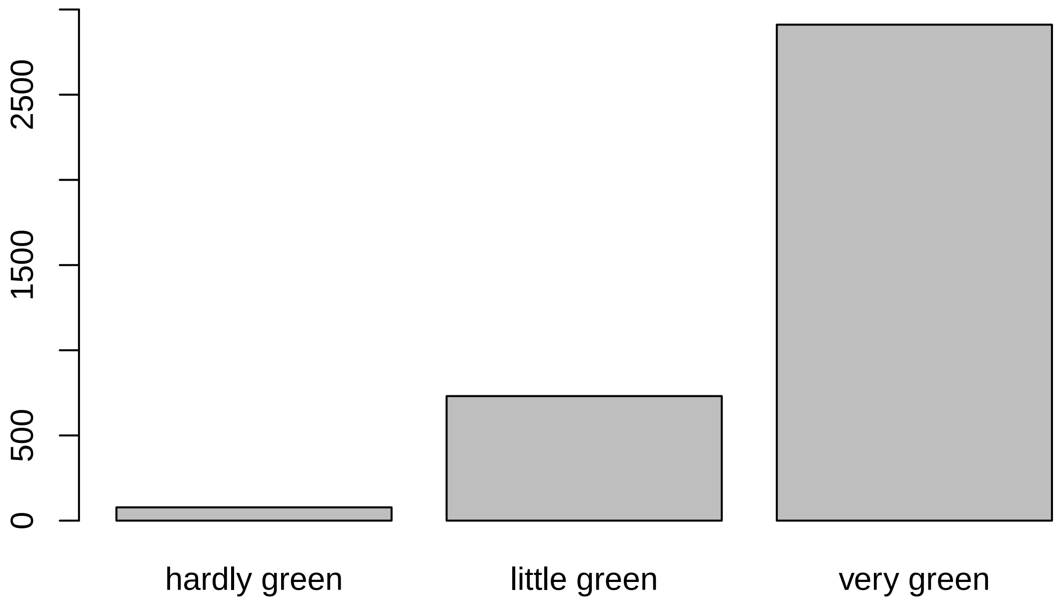

CART

```
set.seed(7890)

greenness_formula = greenness ~ SexAtBirth +  SGIdentity +   SGExpressionLooks +
  SGExpressionBehavior + SGExpressionSAGE +
  SGRolesFemMascuFeeling +   SGRolesFemMascuWish +
  SGRolesFemMascuChange +   SGRolesBothIncomeContribute + SGRolesMenMoneyWomenHouse +
  SGRolesGoodRelationshipWorkingMom + SGRolesWomenWorkChildSuffer +
  SGRolesWomenWorkFamilySuffer + SGRolesHousewifeFulfilling +
  SGRolesHousehusbandFulfilling + SGRolesSingleParentEqual +
  SGRolesSameSexEqual + CareActivitiesChildren +
  CareActivitiesSick + CareActivitiesCooking +
  CareActivitiesHousework + CareActivitiesErrands +
  CareActivitiesAdministrativeTasks +
  CareActivitiesHandicraft + CareActivitiesGardening +
  DiscriminationSocialPosition + DiscriminationAge +
  DiscriminationHeight + DiscriminationWeight +
  DiscriminationDisability + DiscriminationEthnicity +
```

```r
  DiscriminationSG + DiscriminationSexualOrientation +
  DiscriminationAskedifParentsBornAbroad + SGRelationsIncome +
  SGRelationsEmployment + SGRelationsSchoolEducation +
  SGRelationsVocationalEducation + SGRelationsFamilySituation +
  SGRelationsUrbanisation

tree_greenness = rpart(formula = greenness_formula, method = 'class',
                       data = KORA, parms = list(split='Gini'), cp = 0.001,
                       xval = 10, usesurrogate = 2,
                       minbucket = 50, maxdepth = 4)

printcp(tree_greenness)
```

```
##
## Classification tree:
## rpart(formula = greenness_formula, data = KORA, method = "class",
##     parms = list(split = "Gini"), cp = 0.001, xval = 10, usesurrogate = 2,
##     minbucket = 50, maxdepth = 4)
##
## Variables actually used in tree construction:
## [1] CareActivitiesGardening    CareActivitiesSick
## [3] SGRelationsIncome          SGRolesHousewifeFulfilling
##
## Root node error: 809/3720 = 0.21747
##
## n=3720 (22 observations deleted due to missingness)
##
##          CP nsplit rel error xerror     xstd
## 1 0.0061805      0   1.00000 1.0000 0.031101
## 2 0.0010000      4   0.97281 1.0222 0.031348
```

Pruning

```r
tree_greenness = prune(tree_greenness,cp=0.01)

plot(as.party(tree_greenness),main='CART: greenness',
     ep_args = list(justmin = 15),gp = gpar(fontsize = 10))
```

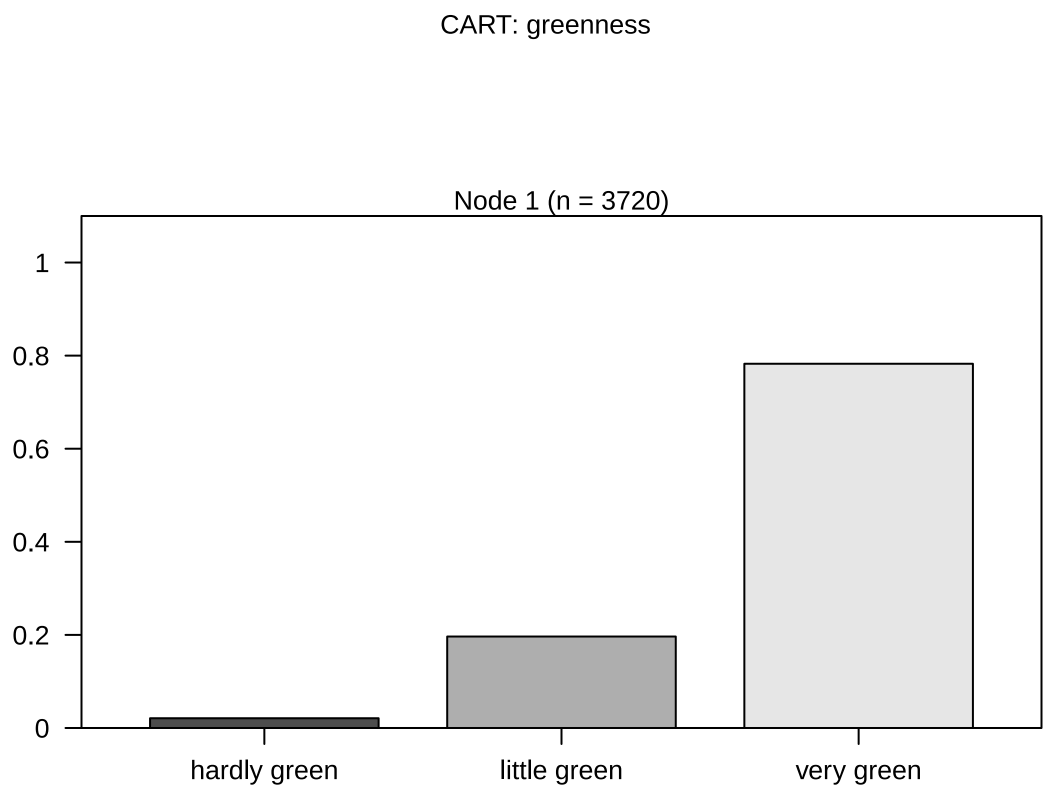

Summary

```
summary(tree_greenness)
```

```
## Call:
## rpart(formula = greenness_formula, data = KORA, method = "class",
##     parms = list(split = "Gini"), cp = 0.001, xval = 10, usesurrogate = 2,
##     minbucket = 50, maxdepth = 4)
##   n=3720 (22 observations deleted due to missingness)
##
##     CP nsplit rel error xerror       xstd
## 1 0.01      0         1      1 0.03110107
##
## Node number 1: 3720 observations
##   predicted class=very green  expected loss=0.2174731  P(node) =1
##     class counts:    78   731  2911
##    probabilities: 0.021 0.197 0.783
```

CIT

```
set.seed(8901)

ctree_greenness = ctree(formula = greenness_formula,
                        data = KORA[!is.na(KORA$greenness),],
                        control = ctree_control(maxsurrogate = 5,
```

```
                                      minbucket = 50,
                                      alpha = 0.05,
                                      testtype = "Bonferroni",
                                      maxdepth = 4))

plot(ctree_greenness,main='CIT: greenness',
     ep_args = list(justmin = 15),gp = gpar(fontsize = 10))
```

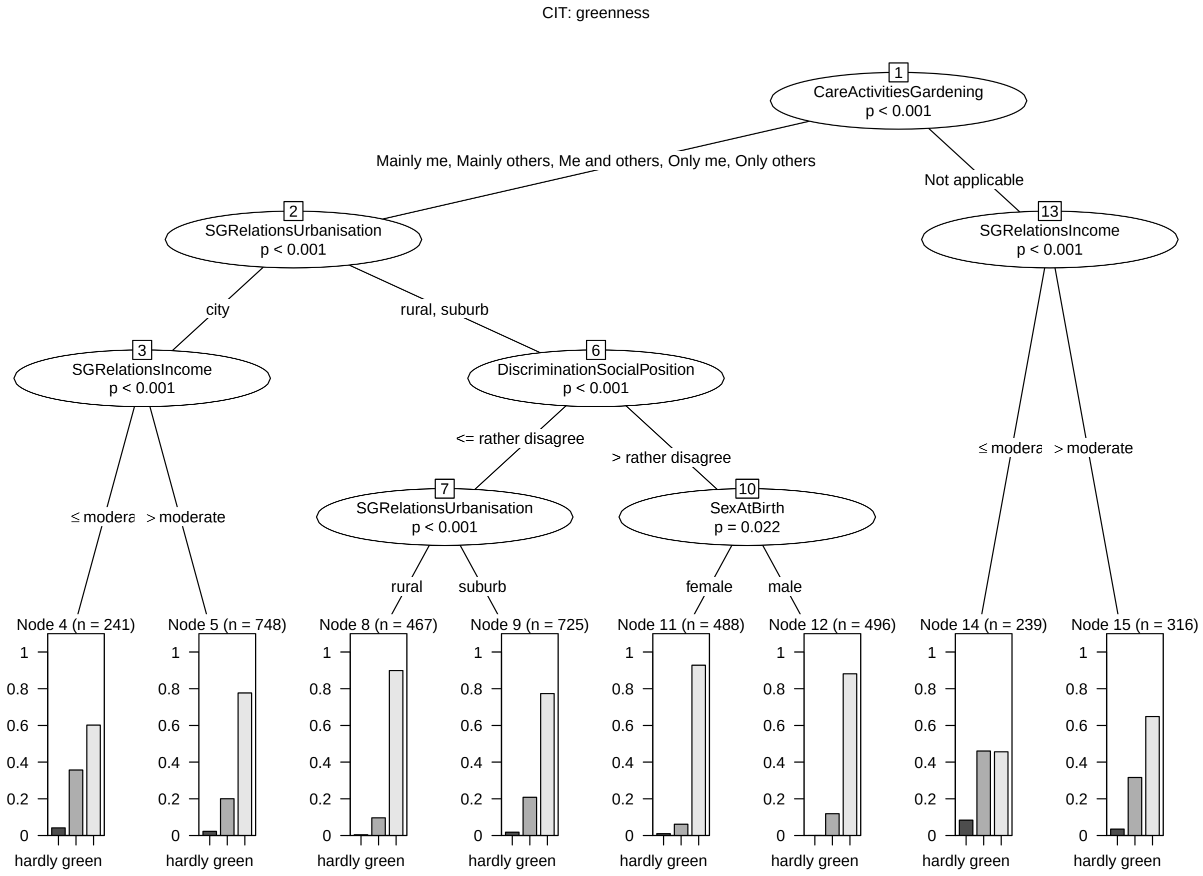

Variable importance via random forests

```
set.seed(9012)

cforest_greenness = cforest(formula = greenness_formula,
                            data=KORA[!is.na(KORA$greenness),],
                            control = ctree_control(testtype = "Univariate",
                                                    mincriterion = 0.95))
Variable_importance_greenness = varimp(cforest_greenness)
```

Variable importance plot

```
colors_greenness = c(rep('cornflowerblue',
                         times = sum(Variable_importance_greenness <
```

```
                                       abs(min(Variable_importance_greenness)))),
                          rep('blue3',
                              times = sum(Variable_importance_greenness >
                                       abs(min(Variable_importance_greenness)))))

par(mar=c(5,55,4,1)+.1)
barplot(sort(Variable_importance_greenness), space = 0.75,
        names.arg= rownames(Variable_importance_greenness),
        col = colors_greenness,
        horiz = TRUE, cex.names = 3.5, cex = 0.45,cex.axis=4, las = 1)
```

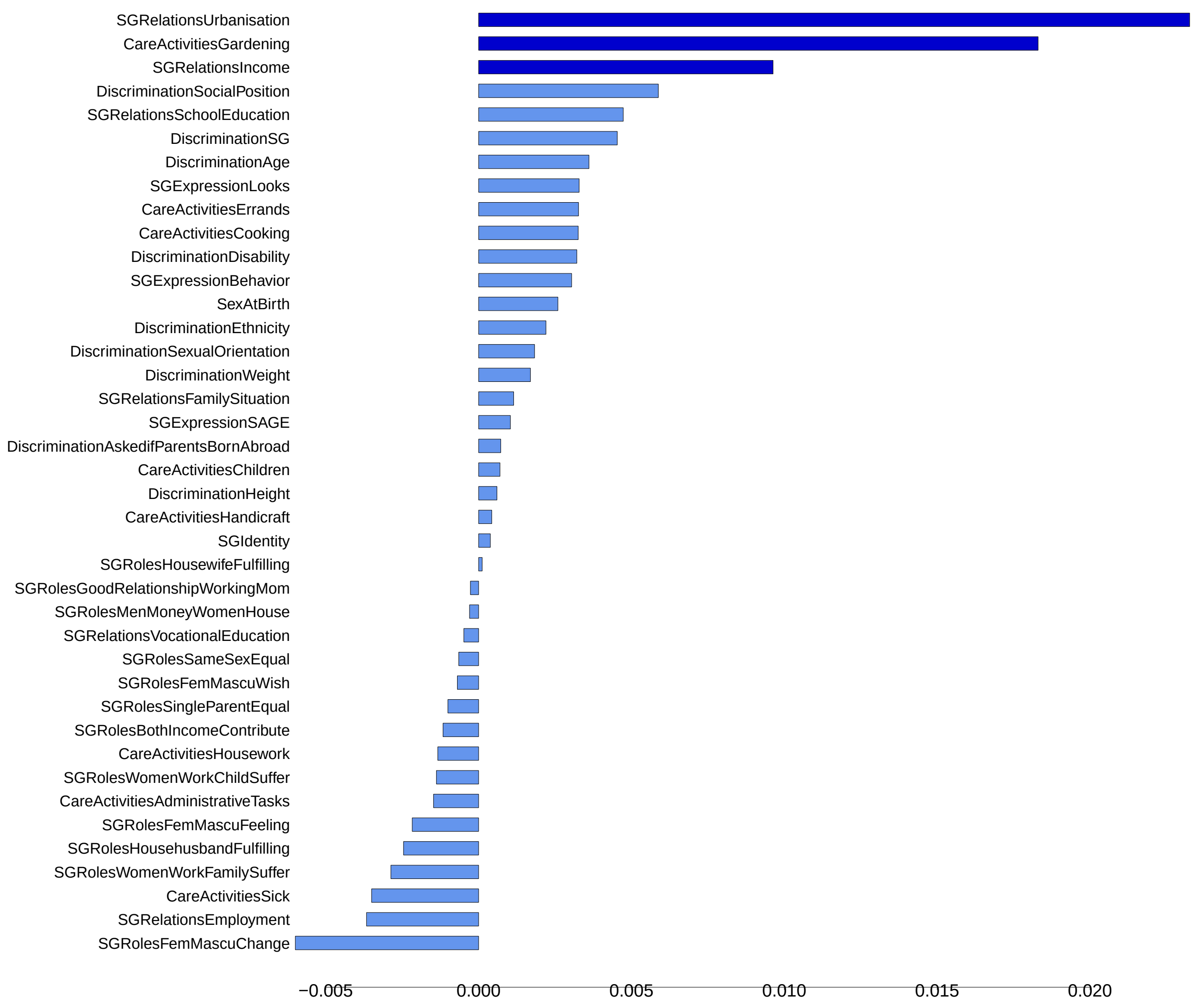

Analysis of exposure variable "NDVI_mean_300_19"

Density plot

```
ggdensity(KORA$NDVI_mean_300_19, xlab='NDVI_mean_300_19')
```

```
## Warning: Removed 11 rows containing non-finite values (stat_density).
```

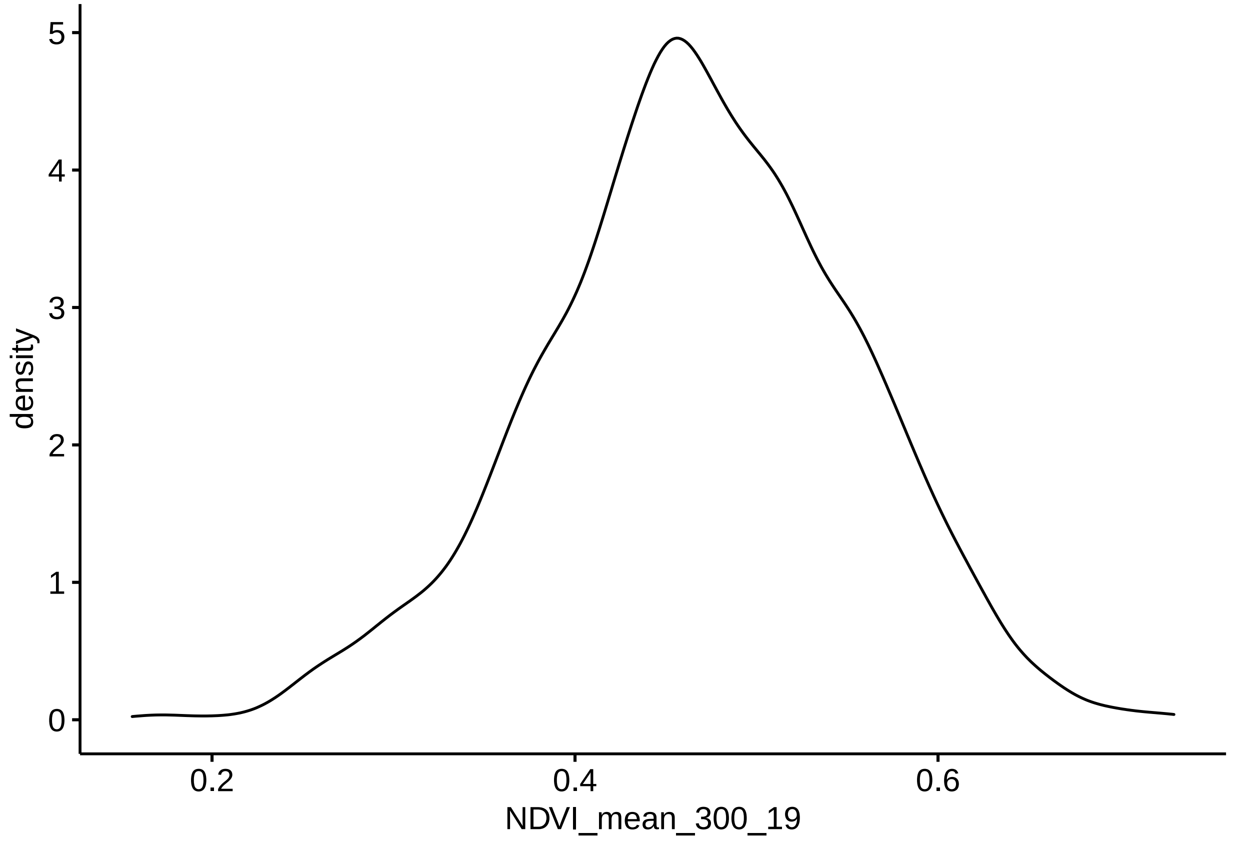

CART

```
set.seed(12345)

NDVI_mean_300_19_formula = NDVI_mean_300_19 ~ SexAtBirth +  SGIdentity +
  SGExpressionLooks + SGExpressionBehavior + SGExpressionSAGE +
  SGRolesFemMascuFeeling +   SGRolesFemMascuWish +
  SGRolesFemMascuChange +   SGRolesBothIncomeContribute + SGRolesMenMoneyWomenHouse +
  SGRolesGoodRelationshipWorkingMom + SGRolesWomenWorkChildSuffer +
  SGRolesWomenWorkFamilySuffer + SGRolesHousewifeFulfilling +
  SGRolesHousehusbandFulfilling + SGRolesSingleParentEqual +
  SGRolesSameSexEqual + CareActivitiesChildren +
  CareActivitiesSick + CareActivitiesCooking +
  CareActivitiesHousework + CareActivitiesErrands +
  CareActivitiesAdministrativeTasks +
  CareActivitiesHandicraft + CareActivitiesGardening +
  DiscriminationSocialPosition + DiscriminationAge +
  DiscriminationHeight + DiscriminationWeight +
```

```r
  DiscriminationDisability + DiscriminationEthnicity +
  DiscriminationSG + DiscriminationSexualOrientation +
  DiscriminationAskedifParentsBornAbroad + SGRelationsIncome +
  SGRelationsEmployment + SGRelationsSchoolEducation +
  SGRelationsVocationalEducation + SGRelationsFamilySituation +
  SGRelationsUrbanisation

tree_NDVI_mean_300_19 = rpart(formula = NDVI_mean_300_19_formula,
                              data = KORA, method = 'anova', cp = 0.001,
                              xval = 10, usesurrogate = 2,
                              minbucket = 50, maxdepth = 4)

printcp(tree_NDVI_mean_300_19)
```

```
##
## Regression tree:
## rpart(formula = NDVI_mean_300_19_formula, data = KORA, method = "anova",
##     cp = 0.001, xval = 10, usesurrogate = 2, minbucket = 50,
##     maxdepth = 4)
##
## Variables actually used in tree construction:
## [1] CareActivitiesGardening
## [2] CareActivitiesSick
## [3] DiscriminationAskedifParentsBornAbroad
## [4] DiscriminationSexualOrientation
## [5] SGRelationsUrbanisation
## [6] SGRolesSameSexEqual
## [7] SGRolesSingleParentEqual
##
## Root node error: 27.343/3731 = 0.0073286
##
## n=3731 (11 observations deleted due to missingness)
##
##          CP nsplit rel error  xerror     xstd
## 1 0.1906601      0   1.00000 1.00059 0.023117
## 2 0.0690629      1   0.80934 0.80995 0.018953
## 3 0.0057850      2   0.74028 0.74137 0.017901
## 4 0.0041598      3   0.73449 0.73957 0.017920
## 5 0.0024499      4   0.73033 0.73343 0.017873
## 6 0.0021619      5   0.72788 0.73698 0.017934
## 7 0.0019251      7   0.72356 0.73825 0.018072
## 8 0.0014922      8   0.72163 0.73846 0.018098
## 9 0.0010000      9   0.72014 0.73759 0.018093
```

Pruning

```r
tree_NDVI_mean_300_19 = prune(tree_NDVI_mean_300_19,cp=0.0025)

plot(as.party(tree_NDVI_mean_300_19),main='CART: NDVI_mean_300_19',
     ep_args = list(justmin = 15),gp = gpar(fontsize = 10))
```

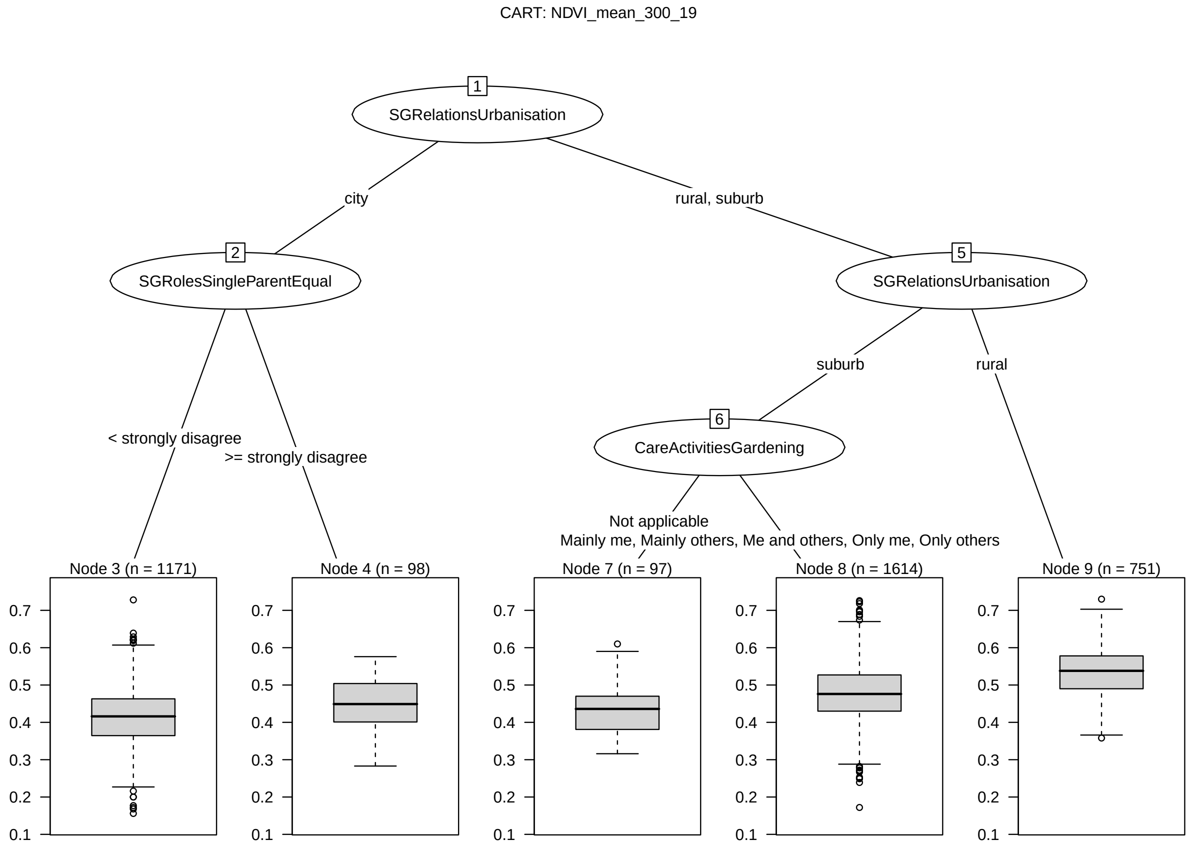

Summary

```r
summary(tree_NDVI_mean_300_19)
```

```
## Call:
## rpart(formula = NDVI_mean_300_19_formula, data = KORA, method = "anova",
##     cp = 0.001, xval = 10, usesurrogate = 2, minbucket = 50,
##     maxdepth = 4)
##   n=3731 (11 observations deleted due to missingness)
##
##            CP nsplit rel error    xerror       xstd
## 1 0.190660138      0 1.0000000 1.0005925 0.02311657
## 2 0.069062908      1 0.8093399 0.8099540 0.01895320
## 3 0.005784976      2 0.7402770 0.7413708 0.01790062
## 4 0.004159820      3 0.7344920 0.7395678 0.01791950
## 5 0.002500000      4 0.7303322 0.7334306 0.01787315
##
## Variable importance
##  SGRelationsUrbanisation  CareActivitiesGardening SGRolesSingleParentEqual
##                       88                       11                        1
##
## Node number 1: 3731 observations,    complexity param=0.1906601
##   mean=0.4672581, MSE=0.00732857
##   left son=2 (1269 obs) right son=3 (2462 obs)
```

```
##   Primary splits:
##       SGRelationsUrbanisation    splits as  LRR,    improve=0.199299200, (449 missing)
##       CareActivitiesGardening    splits as  RRRLRR, improve=0.051650980, (42 missing)
##       CareActivitiesChildren     splits as  RRRLLL, improve=0.014283030, (48 missing)
##       SGRelationsFamilySituation splits as  LR,     improve=0.014107380, (107 missing)
##       CareActivitiesErrands      splits as  LRRRR,  improve=0.008154075, (68 missing)
##   Surrogate splits:
##       CareActivitiesGardening splits as  RRRLRR, agree=0.696, adj=0.124, (440 split)
##
## Node number 2: 1269 observations,    complexity param=0.00415982
##   mean=0.4151923, MSE=0.006087616
##   left son=4 (1171 obs) right son=5 (98 obs)
##   Primary splits:
##       SGRolesSingleParentEqual     splits as  LLLLR,  improve=0.014767770, (15 missing)
##       SGRolesSameSexEqual          splits as  LLLRR,  improve=0.011944160, (22 missing)
##       SGRolesBothIncomeContribute  splits as  LLRRR,  improve=0.006713226, (14 missing)
##       SGRolesWomenWorkFamilySuffer splits as  RRRLL,  improve=0.004803301, (18 missing)
##       CareActivitiesSick           splits as  RLLLLR, improve=0.004720899, (19 missing)
##
## Node number 3: 2462 observations,    complexity param=0.06906291
##   mean=0.4940946, MSE=0.005850736
##   left son=6 (1711 obs) right son=7 (751 obs)
##   Primary splits:
##       SGRelationsUrbanisation    splits as  -RL,    improve=0.104212600, (319 missing)
##       CareActivitiesGardening    splits as  RRRLRR, improve=0.013360630, (29 missing)
##       SGRelationsFamilySituation splits as  LR,     improve=0.005865857, (75 missing)
##       CareActivitiesChildren     splits as  RRRLLL, improve=0.005529691, (35 missing)
##       SGRolesMenMoneyWomenHouse  splits as  RRRLL,  improve=0.004575855, (40 missing)
##
## Node number 4: 1171 observations
##   mean=0.4124535, MSE=0.006101556
##
## Node number 5: 98 observations
##   mean=0.4479184, MSE=0.004760422
##
## Node number 6: 1711 observations,    complexity param=0.005784976
##   mean=0.4757463, MSE=0.00564766
##   left son=12 (97 obs) right son=13 (1614 obs)
##   Primary splits:
##       CareActivitiesGardening         splits as  RRRLRR, improve=0.016621850, (25 missing)
##       DiscriminationSexualOrientation splits as  LLLLR,  improve=0.007730524, (32 missing)
##       SGRelationsFamilySituation      splits as  LR,     improve=0.007231585, (48 missing)
##       CareActivitiesChildren          splits as  RRRRLL, improve=0.006186090, (28 missing)
##       DiscriminationSG                splits as  LLLLR,  improve=0.005491426, (18 missing)
##
## Node number 7: 751 observations
##   mean=0.5358975, MSE=0.003798915
##
## Node number 12: 97 observations
##   mean=0.4365258, MSE=0.004311961
##
## Node number 13: 1614 observations
##   mean=0.4781035, MSE=0.00562993
```

CIT

```
set.seed(23456)

ctree_NDVI_mean_300_19 = ctree(formula = NDVI_mean_300_19_formula,
                               data = KORA[!is.na(KORA$NDVI_mean_300_19),],
                               control = ctree_control(maxsurrogate = 5,
                                                       alpha = 0.05,
                                                       minbucket=50,
                                                       testtype = "Bonferroni",
                                                       maxdepth = 4))

plot(ctree_NDVI_mean_300_19, main='CIT: NDVI_mean_300_19',
     ep_args = list(justmin = 15),gp = gpar(fontsize = 10))
```

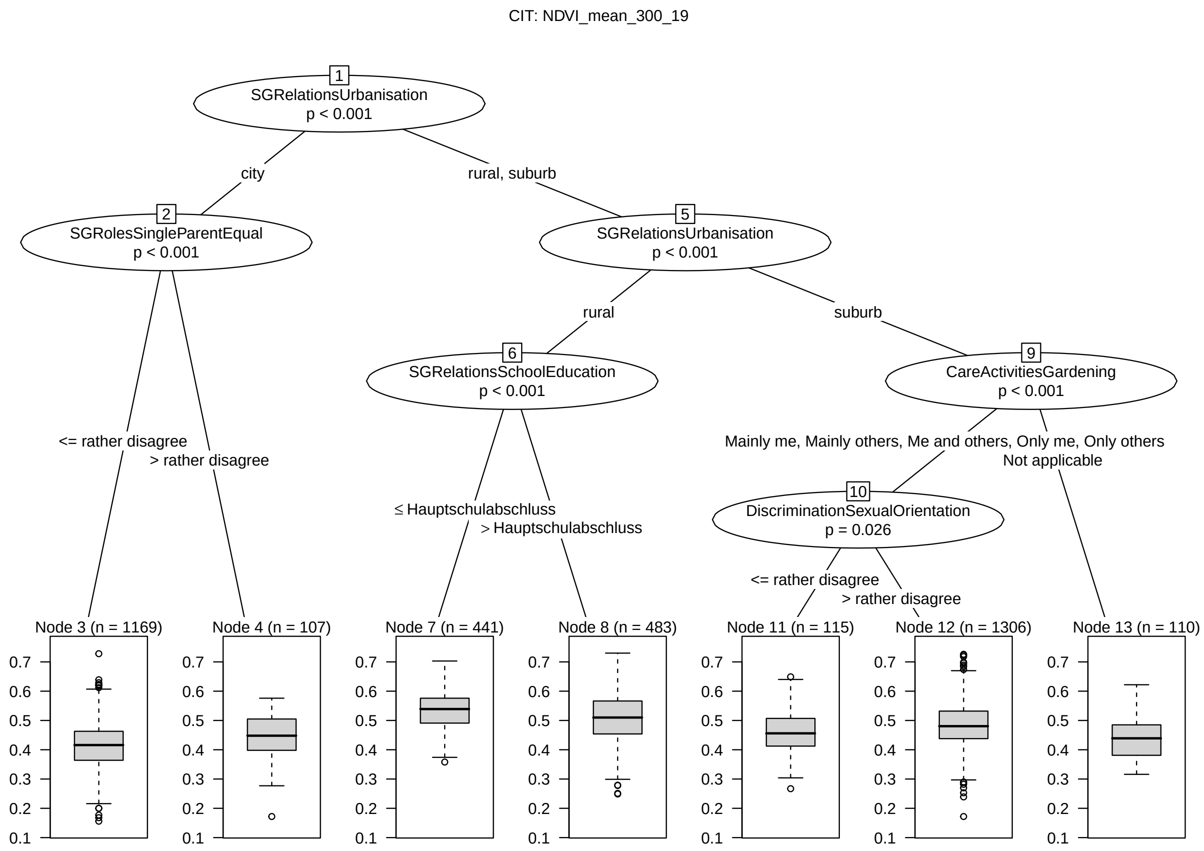

Variable importance via random forests

```
set.seed(34567)

cforest_NDVI_mean_300_19 = cforest(formula = NDVI_mean_300_19_formula,
                                   data=KORA[!is.na(KORA$NDVI_mean_300_19),],
                                   control = ctree_control(testtype = "Univariate",
                                                           mincriterion = 0.95))
```

```
Variable_importance_NDVI_mean_300_19 = varimp(cforest_NDVI_mean_300_19)
```

Variable importance plot

```
colors_NDVI_mean_300_19 = c(rep('cornflowerblue',
                                times = sum(Variable_importance_NDVI_mean_300_19 <
                                            abs(min(Variable_importance_NDVI_mean_300_19)))),
                            rep('blue3',
                                times = sum(Variable_importance_NDVI_mean_300_19 >
                                            abs(min(Variable_importance_NDVI_mean_300_19)))))

par(mar=c(5,55,4,1)+.1)
barplot(sort(Variable_importance_NDVI_mean_300_19), space = 0.75,
        col = colors_NDVI_mean_300_19,
        names.arg= rownames(Variable_importance_NDVI_mean_300_19),
        horiz = TRUE, cex.names = 3.5, cex = 0.45,cex.axis=4, las = 1)
```

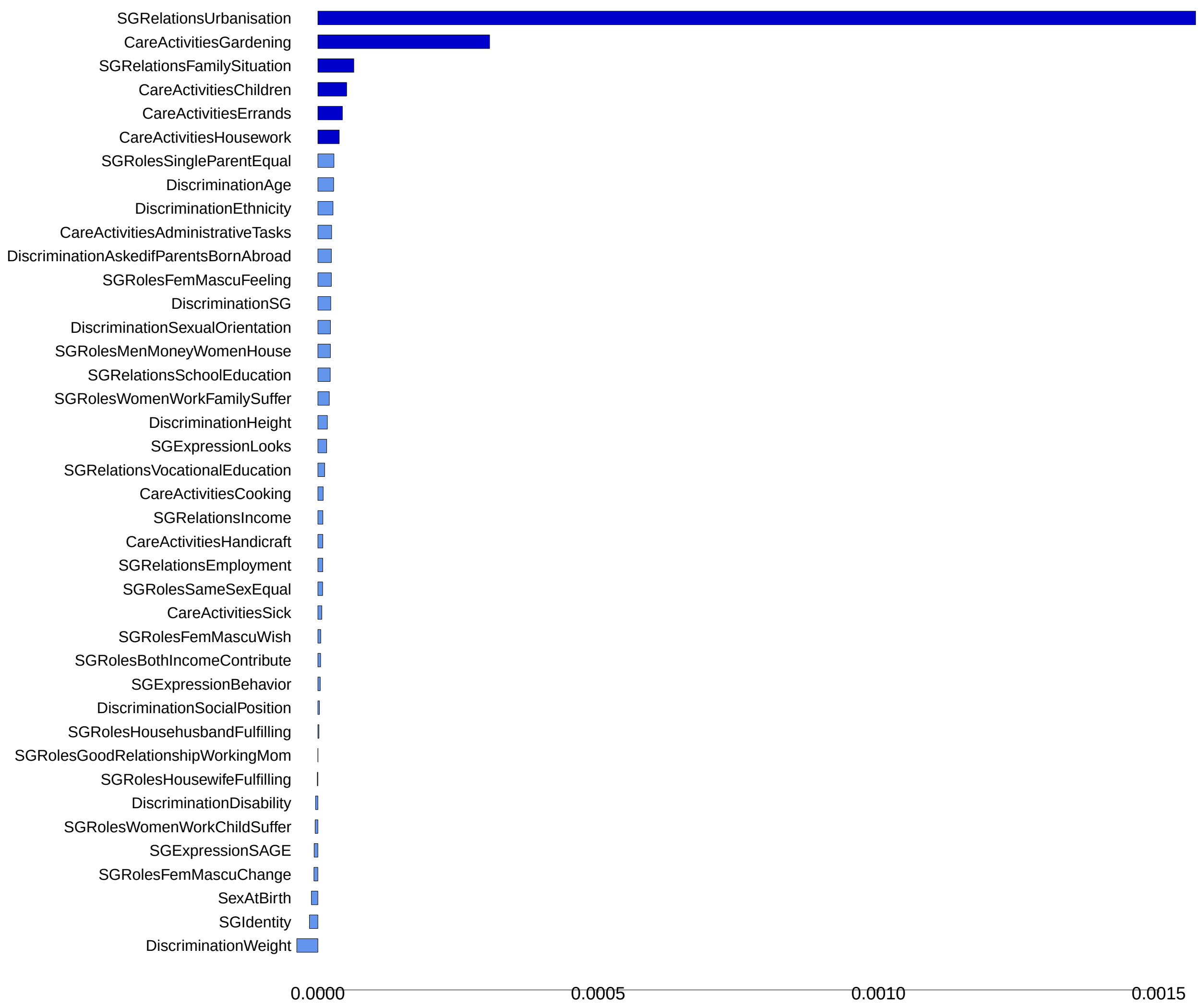

Analysis of exposure variable "NDVI_mean_1000_19"

Density plot

```
ggdensity(KORA$NDVI_mean_1000_19, xlab='NDVI_mean_1000_19')
```

```
## Warning: Removed 11 rows containing non-finite values (stat_density).
```

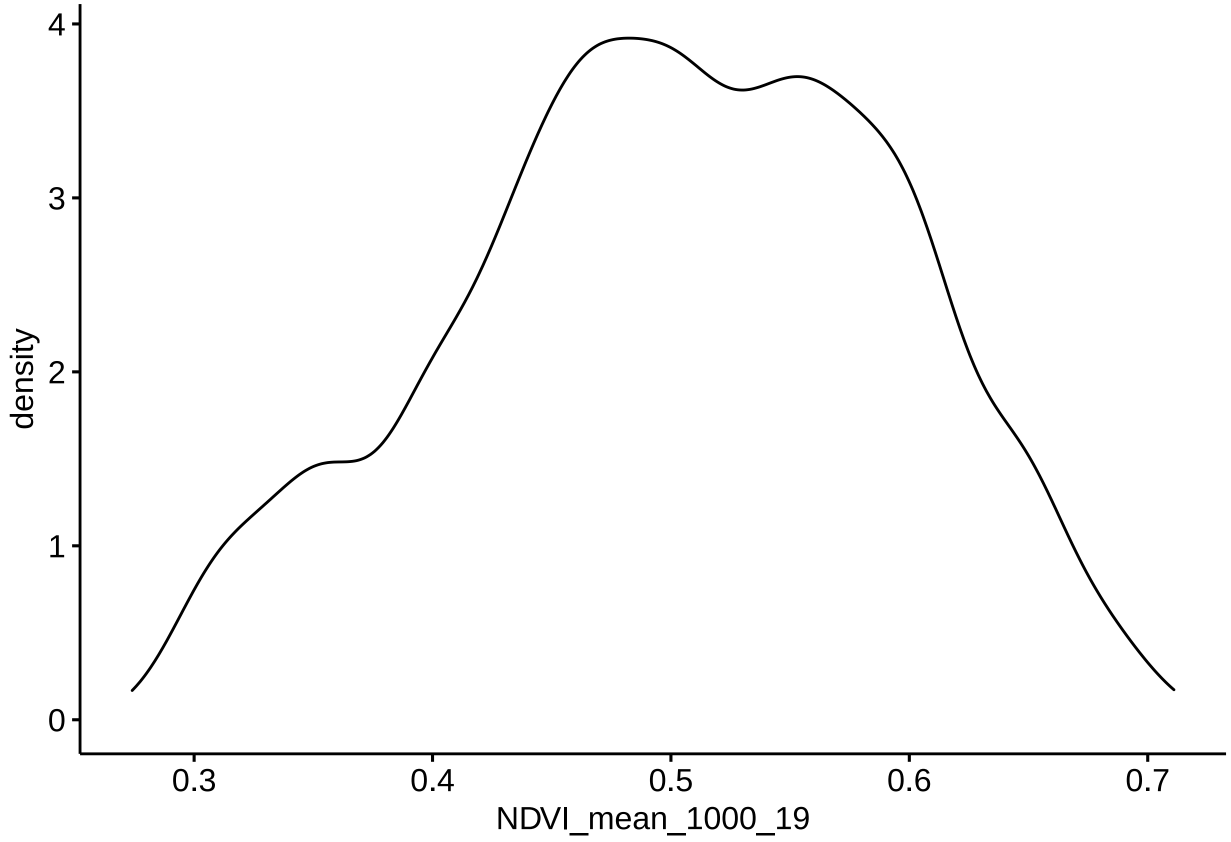

CART

```
set.seed(45678)

NDVI_mean_1000_19_formula = NDVI_mean_1000_19 ~ SexAtBirth +  SGIdentity +
  SGExpressionLooks +   SGExpressionBehavior + SGExpressionSAGE +
  SGRolesFemMascuFeeling +   SGRolesFemMascuWish +
  SGRolesFemMascuChange +   SGRolesBothIncomeContribute + SGRolesMenMoneyWomenHouse +
  SGRolesGoodRelationshipWorkingMom + SGRolesWomenWorkChildSuffer +
  SGRolesWomenWorkFamilySuffer + SGRolesHousewifeFulfilling +
  SGRolesHousehusbandFulfilling + SGRolesSingleParentEqual +
  SGRolesSameSexEqual + CareActivitiesChildren +
  CareActivitiesSick + CareActivitiesCooking +
  CareActivitiesHousework + CareActivitiesErrands +
  CareActivitiesAdministrativeTasks +
  CareActivitiesHandicraft + CareActivitiesGardening +
  DiscriminationSocialPosition + DiscriminationAge +
  DiscriminationHeight + DiscriminationWeight +
```

```r
  DiscriminationDisability + DiscriminationEthnicity +
  DiscriminationSG + DiscriminationSexualOrientation +
  DiscriminationAskedifParentsBornAbroad + SGRelationsIncome +
  SGRelationsEmployment + SGRelationsSchoolEducation +
  SGRelationsVocationalEducation + SGRelationsFamilySituation +
  SGRelationsUrbanisation

tree_NDVI_mean_1000_19 = rpart(formula = NDVI_mean_1000_19_formula,
                               data = KORA, method = 'anova', cp = 0.001,
                               xval = 10, usesurrogate = 2,
                               minbucket = 50, maxdepth = 4)

printcp(tree_NDVI_mean_1000_19)
```

```
##
## Regression tree:
## rpart(formula = NDVI_mean_1000_19_formula, data = KORA, method = "anova",
##     cp = 0.001, xval = 10, usesurrogate = 2, minbucket = 50,
##     maxdepth = 4)
##
## Variables actually used in tree construction:
## [1] CareActivitiesGardening         DiscriminationSexualOrientation
## [3] SGExpressionSAGE                SGRelationsUrbanisation
## [5] SGRolesSameSexEqual
##
## Root node error: 31.759/3731 = 0.0085122
##
## n=3731 (11 observations deleted due to missingness)
##
##          CP nsplit rel error  xerror     xstd
## 1 0.2858880      0   1.00000 1.00028 0.019309
## 2 0.0803427      1   0.71411 0.71457 0.015171
## 3 0.0059200      2   0.63377 0.63441 0.014238
## 4 0.0031707      3   0.62785 0.63165 0.014402
## 5 0.0027295      4   0.62468 0.63219 0.014377
## 6 0.0017698      5   0.62195 0.63277 0.014403
## 7 0.0015803      6   0.62018 0.63249 0.014496
## 8 0.0014385      7   0.61860 0.63308 0.014520
## 9 0.0010000      9   0.61572 0.63415 0.014494
```

Pruning

```r
tree_NDVI_mean_1000_19 = prune(tree_NDVI_mean_1000_19,cp=0.004)

plot(as.party(tree_NDVI_mean_1000_19),main='CART: NDVI_mean_1000_19',
     ep_args = list(justmin = 15),gp = gpar(fontsize = 10))
```

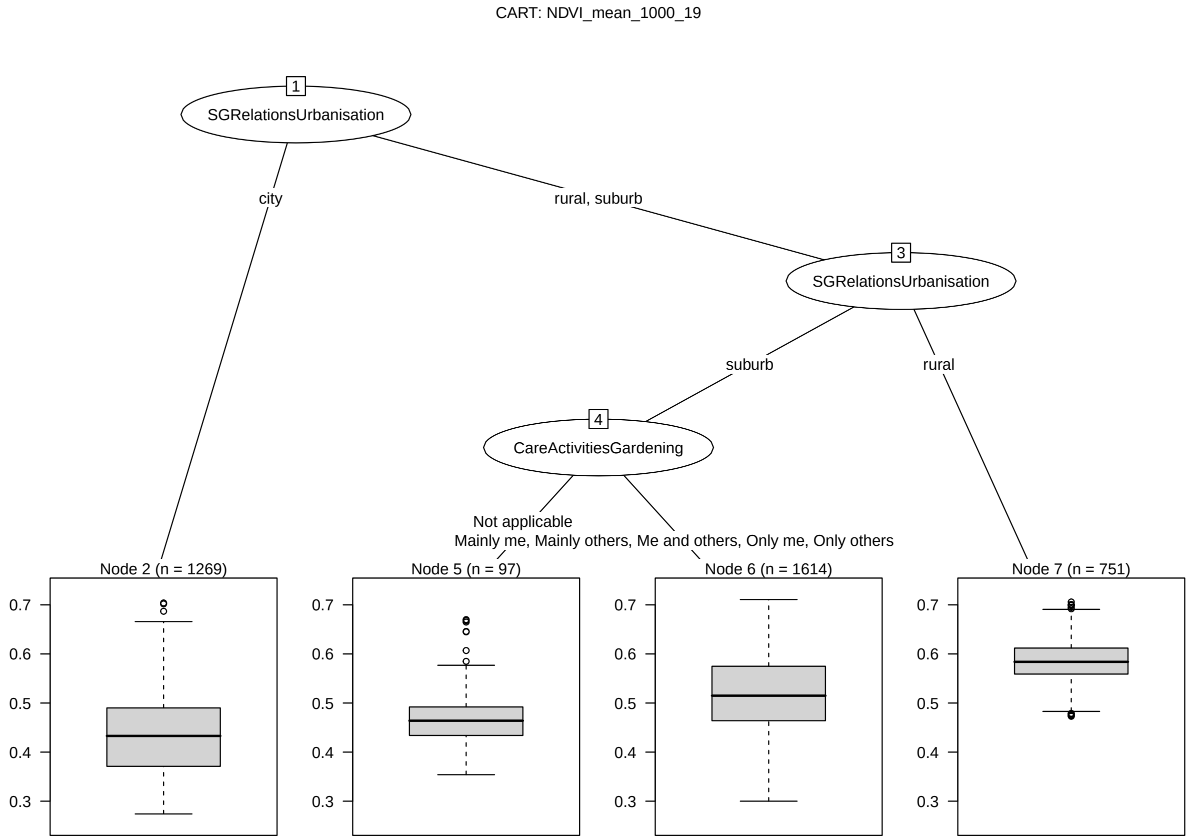

Summary

```
summary(tree_NDVI_mean_1000_19)
```

```
## Call:
## rpart(formula = NDVI_mean_1000_19_formula, data = KORA, method = "anova",
##     cp = 0.001, xval = 10, usesurrogate = 2, minbucket = 50,
##     maxdepth = 4)
##   n=3731 (11 observations deleted due to missingness)
##
##            CP nsplit rel error    xerror       xstd
## 1 0.285888042      0 1.0000000 1.0002797 0.01930874
## 2 0.080342699      1 0.7141120 0.7145706 0.01517138
## 3 0.005920019      2 0.6337693 0.6344081 0.01423767
## 4 0.004000000      3 0.6278492 0.6316461 0.01440234
##
## Variable importance
## SGRelationsUrbanisation CareActivitiesGardening
##                      89                      11
##
## Node number 1: 3731 observations,    complexity param=0.285888
##   mean=0.5016717, MSE=0.008512184
##   left son=2 (1269 obs) right son=3 (2462 obs)
##   Primary splits:
```

```
##       SGRelationsUrbanisation    splits as  LRR,    improve=0.300402900, (449 missing)
##       CareActivitiesGardening    splits as  RRRLRR, improve=0.073012010, (42 missing)
##       SGRelationsFamilySituation splits as  LR,     improve=0.017742450, (107 missing)
##       CareActivitiesChildren     splits as  RRRLLL, improve=0.016301140, (48 missing)
##       SGRolesMenMoneyWomenHouse  splits as  RRRLL,  improve=0.009484892, (55 missing)
##   Surrogate splits:
##       CareActivitiesGardening splits as  RRRLRR, agree=0.696, adj=0.124, (440 split)
##
## Node number 2: 1269 observations
##   mean=0.4329598, MSE=0.006197708
##
## Node number 3: 2462 observations,    complexity param=0.0803427
##   mean=0.5370881, MSE=0.006017287
##   left son=6 (1711 obs) right son=7 (751 obs)
##   Primary splits:
##       SGRelationsUrbanisation    splits as  -RL,    improve=0.137940800, (319 missing)
##       CareActivitiesGardening    splits as  RRRLRR, improve=0.014279870, (29 missing)
##       SGRelationsSchoolEducation splits as  RRL,    improve=0.007951953, (0 missing)
##       SGRolesMenMoneyWomenHouse  splits as  RRRLL,  improve=0.007107882, (40 missing)
##       SGRelationsFamilySituation splits as  LR,     improve=0.005439507, (75 missing)
##
## Node number 6: 1711 observations,    complexity param=0.005920019
##   mean=0.5157598, MSE=0.006205953
##   left son=12 (97 obs) right son=13 (1614 obs)
##   Primary splits:
##       CareActivitiesGardening          splits as  RRRLRR, improve=0.017945630, (25 missing)
##       DiscriminationSexualOrientation splits as  LLLLR,  improve=0.007785832, (32 missing)
##       SGRelationsFamilySituation      splits as  LR,     improve=0.005905132, (48 missing)
##       DiscriminationSG                splits as  LLLLR,  improve=0.005478688, (18 missing)
##       DiscriminationEthnicity         splits as  LLLLR,  improve=0.004245468, (22 missing)
##
## Node number 7: 751 observations
##   mean=0.5856804, MSE=0.002189845
##
## Node number 12: 97 observations
##   mean=0.473, MSE=0.004063773
##
## Node number 13: 1614 observations
##   mean=0.5183296, MSE=0.006218207
```

CIT

```
set.seed(56789)

ctree_NDVI_mean_1000_19 = ctree(formula = NDVI_mean_1000_19_formula,
                                data = KORA[!is.na(KORA$NDVI_mean_1000_19),],
                                control = ctree_control(maxsurrogate = 5,
                                                        alpha = 0.05,
                                                        minbucket=50,
                                                        testtype = "Bonferroni",
                                                        maxdepth = 4))

plot(ctree_NDVI_mean_1000_19,main='CIT: NDVI_mean_1000_19',
```

```
    ep_args = list(justmin = 15),gp = gpar(fontsize = 10))
```

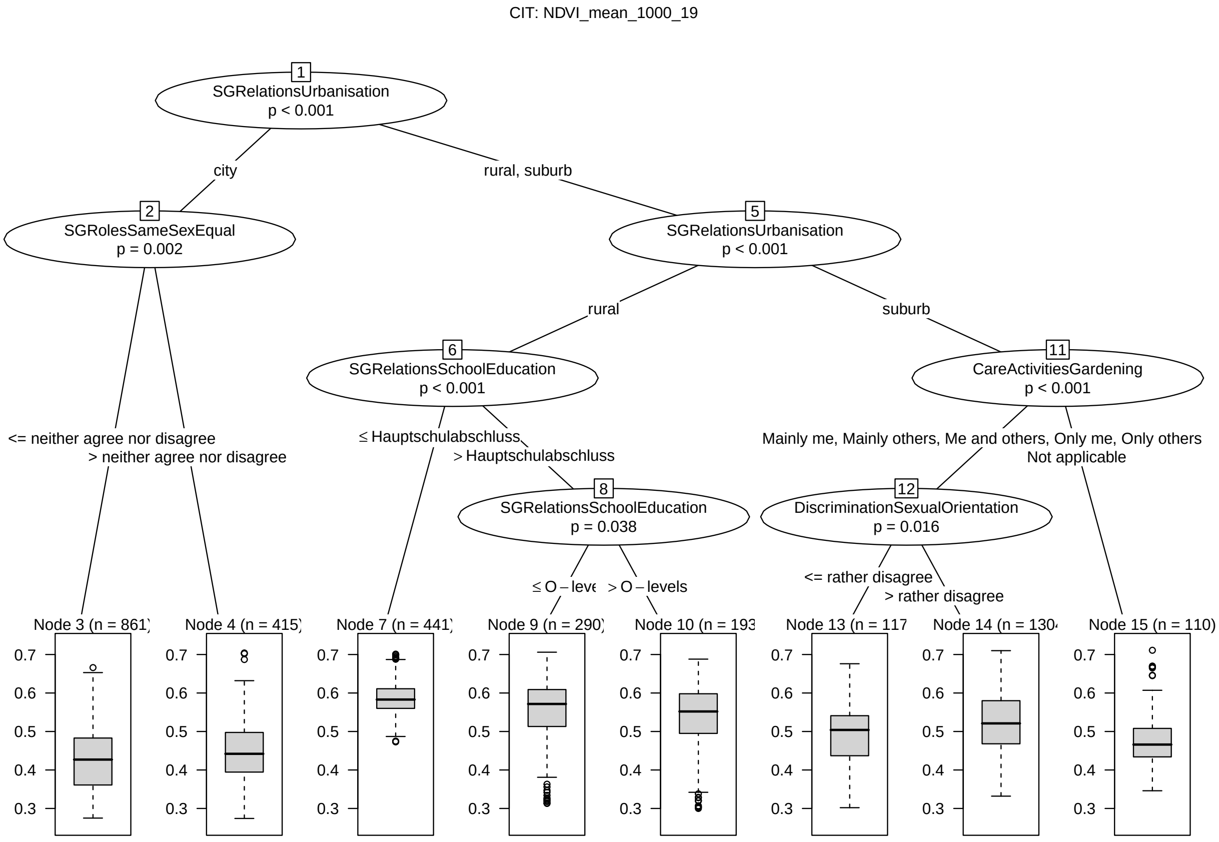

Variable importance via random forests

```
set.seed(67890)

cforest_NDVI_mean_1000_19 = cforest(formula = NDVI_mean_1000_19_formula,
                                     data=KORA[!is.na(KORA$NDVI_mean_1000_19),],
                                    control = ctree_control(testtype = "Univariate",
                                                            mincriterion = 0.95))

Variable_importance_NDVI_mean_1000_19 = varimp(cforest_NDVI_mean_1000_19)
```

Variable importance plot

```
colors_NDVI_mean_1000_19 = c(rep('cornflowerblue',
                                   times = sum(Variable_importance_NDVI_mean_1000_19 <
                                          abs(min(Variable_importance_NDVI_mean_1000_19)))),
                              rep('blue3',
                                   times = sum(Variable_importance_NDVI_mean_1000_19 >
                                          abs(min(Variable_importance_NDVI_mean_1000_19)))))
```

```r
par(mar=c(5,55,4,1)+.1)
barplot(sort(Variable_importance_NDVI_mean_1000_19), space = 0.75,
        col = colors_NDVI_mean_1000_19,
        names.arg= rownames(Variable_importance_NDVI_mean_1000_19),
        horiz = TRUE, cex.names = 3.5, cex = 0.45,cex.axis=4, las = 1)
```

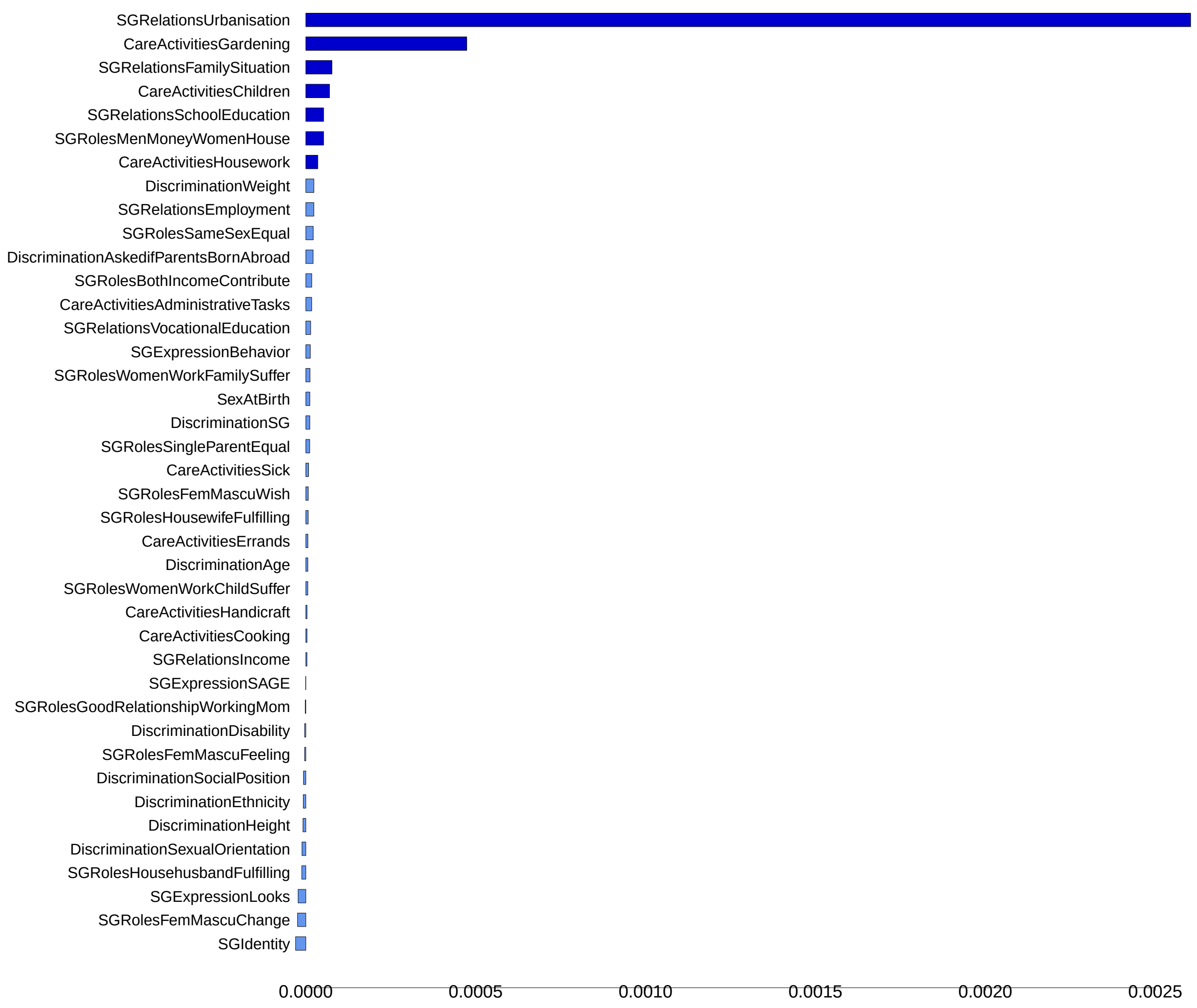

Supplement: Supplementary file 1 [file ijerph-19-07476-s001.zip › ijerph-1725340-supplementary/Supplementary Materials/Supplementary Materials S1_ Results_40Covariates.pdf]
