# Supplementary material for: Decision Tree Analyses to Explore the Relevance of Multiple Sex/Gender Dimensions for the Exposure to Green Spaces: Results from the KORA INGER Study
Source: Int J Environ Res Public Health. 2022 Jun 18;19(12):7476. doi: 10.3390/ijerph19127476 (PMC9224469; doi:10.3390/ijerph19127476)

KORA FIT Analyses

Load required packages and data

```
rm(list = ls())
setwd("D:/Projekte/INGER/KORA_Analysen")

library(rpart)
library(partykit)
```

```
## Warning: package 'partykit' was built under R version 4.0.3
## Loading required package: grid
## Loading required package: libcoin
## Warning: package 'libcoin' was built under R version 4.0.3
## Loading required package: mvtnorm
```

```
library(ggpubr)
```

```
## Warning: package 'ggpubr' was built under R version 4.0.3
## Loading required package: ggplot2
## Warning: package 'ggplot2' was built under R version 4.0.3
```

```
load("KORA_FIT_Analyses.Rda")

KORA = KORA_FIT_Analyses
```

Analysis of exposure variable "publicgreen"

Barplot

```
barplot(table(KORA$publicgreen), ylim=c(0,2500))
```

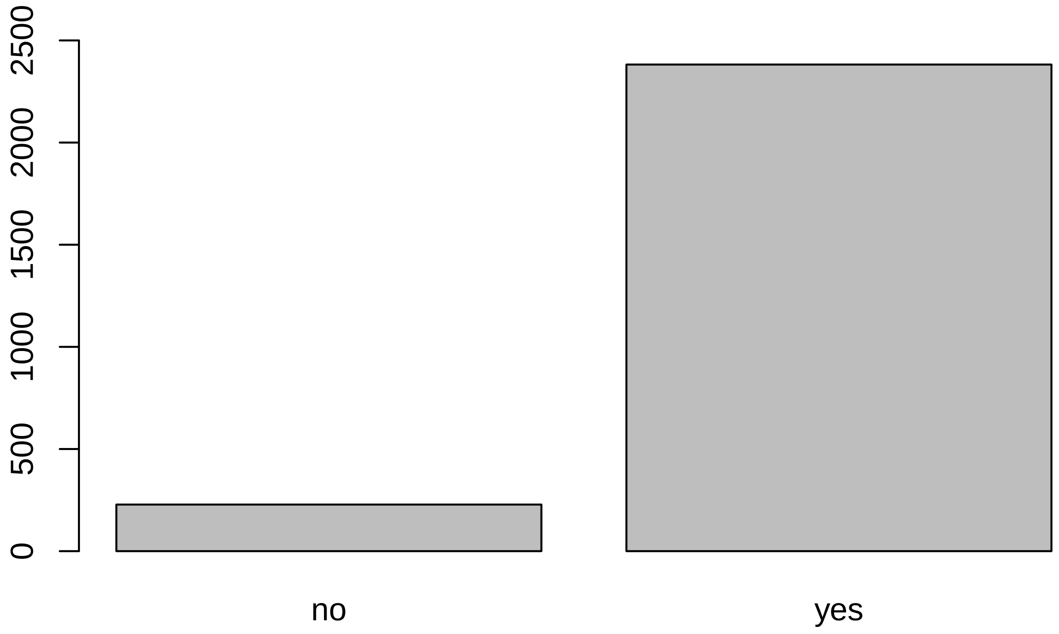

CART

```
set.seed(1234)

publicgreen_formula = publicgreen ~ SexAtBirth +  SGIdentity +
  SGExpressionLooks + SGExpressionSAGE +
  SGExpressionBehavior + SGRolesFemMascuFeeling +
  SGRolesFemMascuWish + SGRolesFemMascuChange +
  SGRolesBothIncomeContribute + SGRolesMenMoneyWomenHouse +
  SGRolesGoodRelationshipWorkingMom + SGRolesWomenWorkChildSuffer +
  SGRolesWomenWorkFamilySuffer + SGRolesHousewifeFulfilling +
  SGRolesHousehusbandFulfilling + SGRolesSingleParentEqual +
  SGRolesSameSexEqual + CareActivitiesChildren +
  CareActivitiesSick + CareActivitiesCooking +
  CareActivitiesHousework + CareActivitiesErrands +
  CareActivitiesAdministrativeTasks +
  CareActivitiesHandicraft + CareActivitiesGardening +
  DiscriminationSocialPosition + DiscriminationAge +
  DiscriminationHeight + DiscriminationWeight +
  DiscriminationDisability + DiscriminationEthnicity +
  DiscriminationSG + DiscriminationSexualOrientation +
  DiscriminationAskedifParentsBornAbroad + SGRelationsIncome +
  SGRelationsEmployment + SGRelationsSchoolEducation +
  SGRelationsVocationalEducation + SGRelationsFamilySituation +
  SGRelationsOccupation + SGRelationsEmploymentCategories +
```

```r
  SGRelationsIncomeEuro + SGRelationsDisability +
  SGRelationsMobility + SGRelationsHouseholdMembers +
  SGRelationsUrbanisation +
  HealthBehaviorAlcohol + HealthBehaviorSmoking +
  HealthBehaviorPhysicalActivity + LifeSatisfaction +
  PerceivedStress + SelfEfficacy + OptimismPessimism

tree_publicgreen = rpart(formula = publicgreen_formula, method = 'class',
                         data = KORA, parms = list(split='Gini'), cp = 0.001,
                         xval = 10, usesurrogate = 2,
                         minbucket = 50, maxdepth = 4)

printcp(tree_publicgreen)
```

```
##
## Classification tree:
## rpart(formula = publicgreen_formula, data = KORA, method = "class",
##     parms = list(split = "Gini"), cp = 0.001, xval = 10, usesurrogate = 2,
##     minbucket = 50, maxdepth = 4)
##
## Variables actually used in tree construction:
## character(0)
##
## Root node error: 228/2610 = 0.087356
##
## n=2610 (14 observations deleted due to missingness)
##
##   CP nsplit rel error xerror xstd
## 1  0      0         1      0    0
```

Pruning

```r
plot(as.party(tree_publicgreen),main='CART: publicgreen',
     ep_args = list(justmin = 15),gp = gpar(fontsize = 10),
     terminal_panel = node_barplot, tp_args = list(beside=TRUE))
```

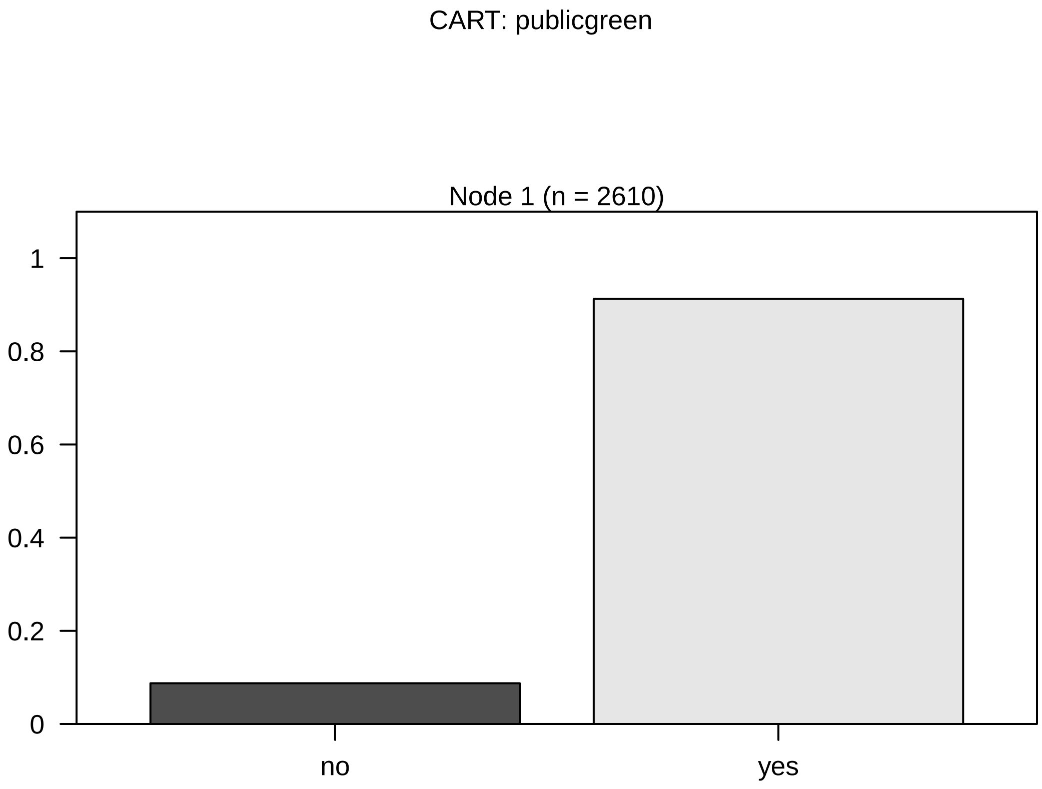

Summary

```r
summary(tree_publicgreen)
```

```
## Call:
## rpart(formula = publicgreen_formula, data = KORA, method = "class",
##     parms = list(split = "Gini"), cp = 0.001, xval = 10, usesurrogate = 2,
##     minbucket = 50, maxdepth = 4)
##   n=2610 (14 observations deleted due to missingness)
##
##   CP nsplit rel error xerror xstd
## 1  0      0         1      0    0
##
## Node number 1: 2610 observations
##   predicted class=yes  expected loss=0.08735632  P(node) =1
##     class counts:   228  2382
##    probabilities: 0.087 0.913
```

CIT

```r
set.seed(2345)

ctree_publicgreen = ctree(formula = publicgreen_formula,
                          data = KORA[!is.na(KORA$publicgreen),],
                          control = ctree_control(maxsurrogate = 5,
```

```
                                                    minbucket=50,
                                                    alpha = 0.05,
                                                    testtype = "Bonferroni",
                                                    maxdepth = 4))

plot(ctree_publicgreen,main='CIT: publicgreen',
     ep_args = list(justmin = 15),gp = gpar(fontsize = 10),
     terminal_panel = node_barplot, tp_args = list(beside=TRUE))
```

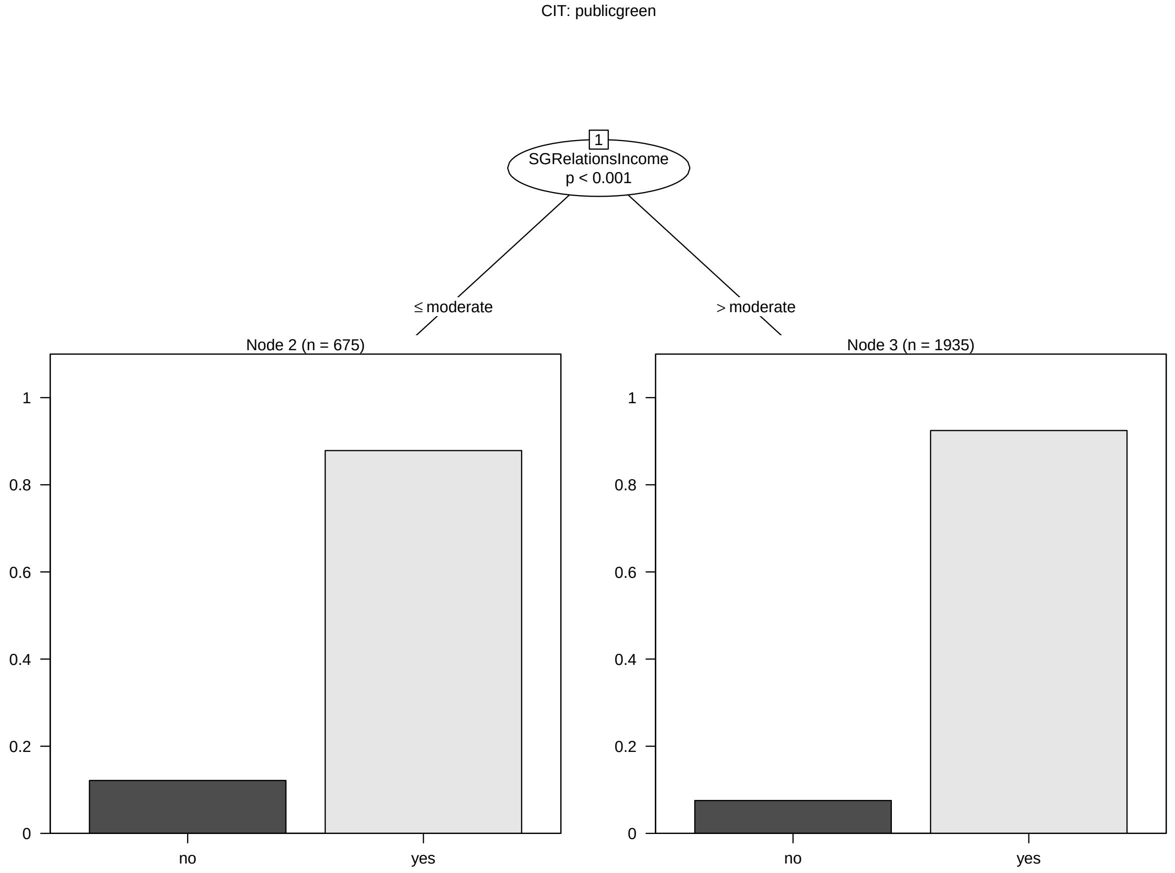

Variable importance via random forests

```
set.seed(3456)

cforest_publicgreen = cforest(formula = publicgreen_formula,
                              data=KORA[!is.na(KORA$publicgreen),],
                              control = ctree_control(testtype = "Univariate",
                                                      mincriterion = 0.95))

Variable_importance_publicgreen = varimp(cforest_publicgreen)
```

Variable importance plot

```r
colors_publicgreen = c(rep('cornflowerblue',
                           times = sum(Variable_importance_publicgreen <
                                       abs(min(Variable_importance_publicgreen)))),
                       rep('blue3',
                           times = sum(Variable_importance_publicgreen >
                                       abs(min(Variable_importance_publicgreen)))))

par(mar=c(5,55,4,1)+.1)
barplot(sort(Variable_importance_publicgreen), space = 0.75,
        names.arg= rownames(Variable_importance_publicgreen),
        col = colors_publicgreen,
        horiz = TRUE, cex.names = 3.5, cex = 0.45,cex.axis=4, las = 1)
```

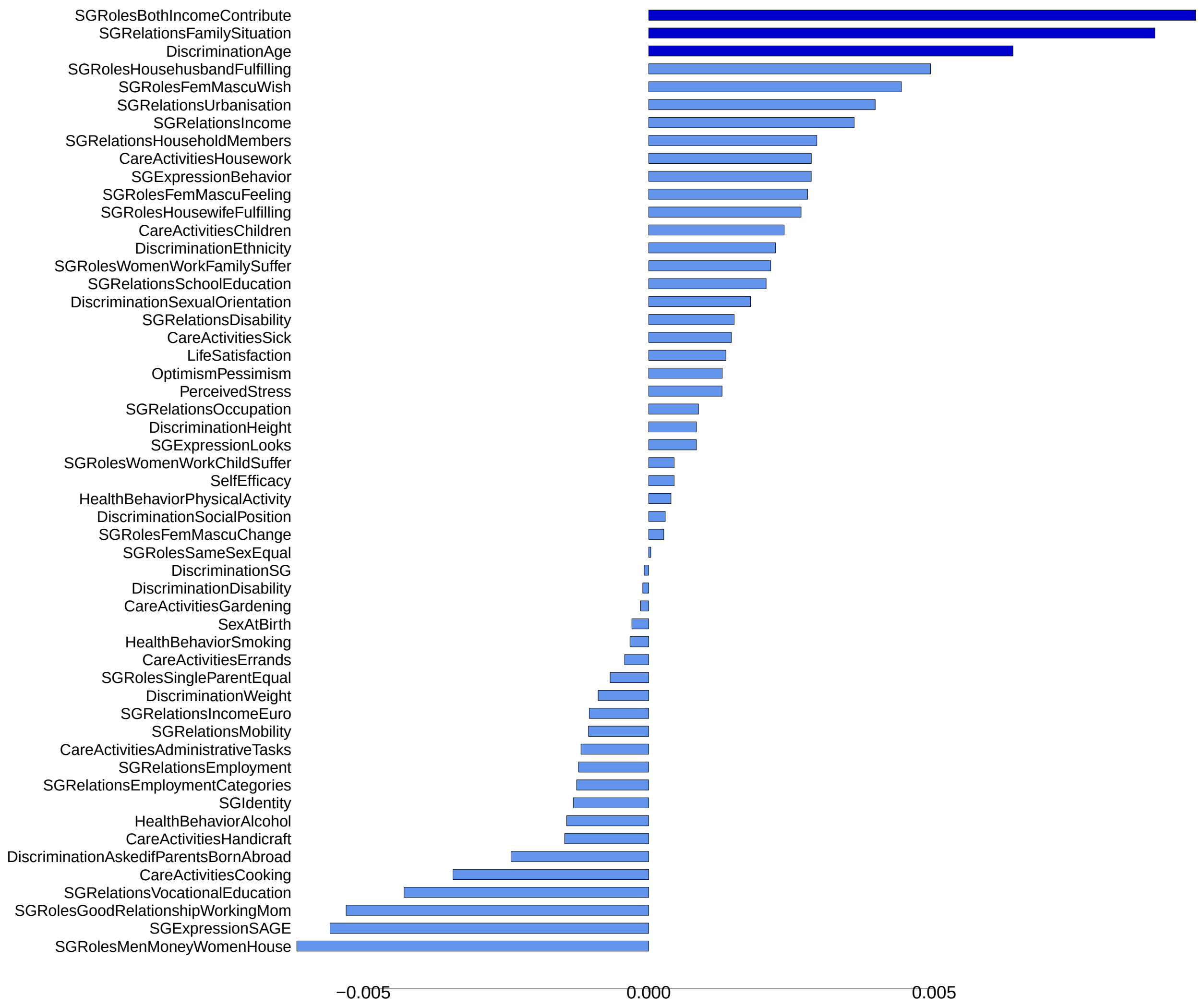

Analysis of exposure variable "qualitypublicgreen"

Barplot

```r
barplot(table(KORA$qualitypublicgreen), ylim=c(0,2000))
```

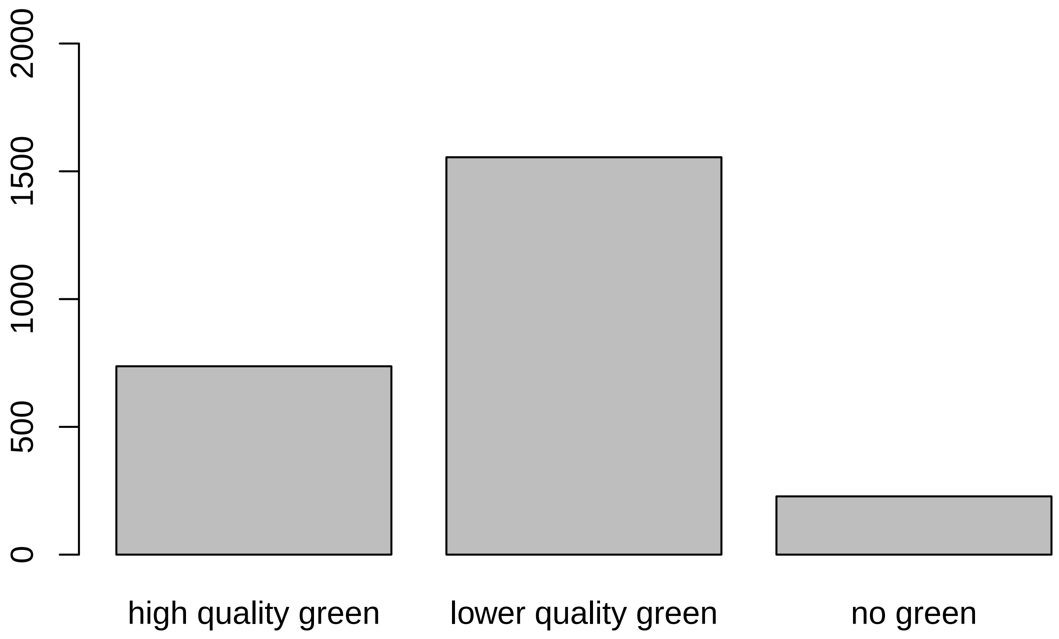

CART

```r
set.seed(4567)

qualitypublicgreen_formula = qualitypublicgreen ~ SexAtBirth +  SGIdentity +
  SGExpressionLooks + SGExpressionSAGE +
  SGExpressionBehavior + SGRolesFemMascuFeeling +
  SGRolesFemMascuWish + SGRolesFemMascuChange +
  SGRolesBothIncomeContribute + SGRolesMenMoneyWomenHouse +
  SGRolesGoodRelationshipWorkingMom + SGRolesWomenWorkChildSuffer +
  SGRolesWomenWorkFamilySuffer + SGRolesHousewifeFulfilling +
  SGRolesHousehusbandFulfilling + SGRolesSingleParentEqual +
  SGRolesSameSexEqual + CareActivitiesChildren +
  CareActivitiesSick + CareActivitiesCooking +
  CareActivitiesHousework + CareActivitiesErrands +
  CareActivitiesAdministrativeTasks +
  CareActivitiesHandicraft + CareActivitiesGardening +
  DiscriminationSocialPosition + DiscriminationAge +
  DiscriminationHeight + DiscriminationWeight +
```

```r
  DiscriminationDisability + DiscriminationEthnicity +
  DiscriminationSG + DiscriminationSexualOrientation +
  DiscriminationAskedifParentsBornAbroad + SGRelationsIncome +
  SGRelationsEmployment + SGRelationsSchoolEducation +
  SGRelationsVocationalEducation + SGRelationsFamilySituation +
  SGRelationsOccupation + SGRelationsEmploymentCategories +
  SGRelationsIncomeEuro + SGRelationsDisability +
  SGRelationsMobility + SGRelationsHouseholdMembers +
  SGRelationsUrbanisation +
  HealthBehaviorAlcohol + HealthBehaviorSmoking +
  HealthBehaviorPhysicalActivity + LifeSatisfaction +
  PerceivedStress + SelfEfficacy + OptimismPessimism

tree_qualitypublicgreen = rpart(formula = qualitypublicgreen_formula, method = 'class',
                                data = KORA, parms = list(split='Gini'), cp = 0.001,
                                xval = 10, usesurrogate = 2,
                                minbucket = 50, maxdepth = 4)
printcp(tree_qualitypublicgreen)
```

```
##
## Classification tree:
## rpart(formula = qualitypublicgreen_formula, data = KORA, method = "class",
##     parms = list(split = "Gini"), cp = 0.001, xval = 10, usesurrogate = 2,
##     minbucket = 50, maxdepth = 4)
##
## Variables actually used in tree construction:
## [1] CareActivitiesHandicraft DiscriminationAge        HealthBehaviorAlcohol
## [4] SGRelationsIncome
##
## Root node error: 965/2520 = 0.38294
##
## n=2520 (104 observations deleted due to missingness)
##
##          CP nsplit rel error  xerror     xstd
## 1 0.0082902      0   1.00000 1.00000 0.025287
## 2 0.0062176      3   0.97306 1.00933 0.025331
## 3 0.0010000      4   0.96684 0.99689 0.025272
```

Pruning

```r
tree_qualitypublicgreen = prune(tree_qualitypublicgreen,cp=0.005)

plot(as.party(tree_qualitypublicgreen),main='CART: qualitypublicgreen',
     ep_args = list(justmin = 15),gp = gpar(fontsize = 10))
```

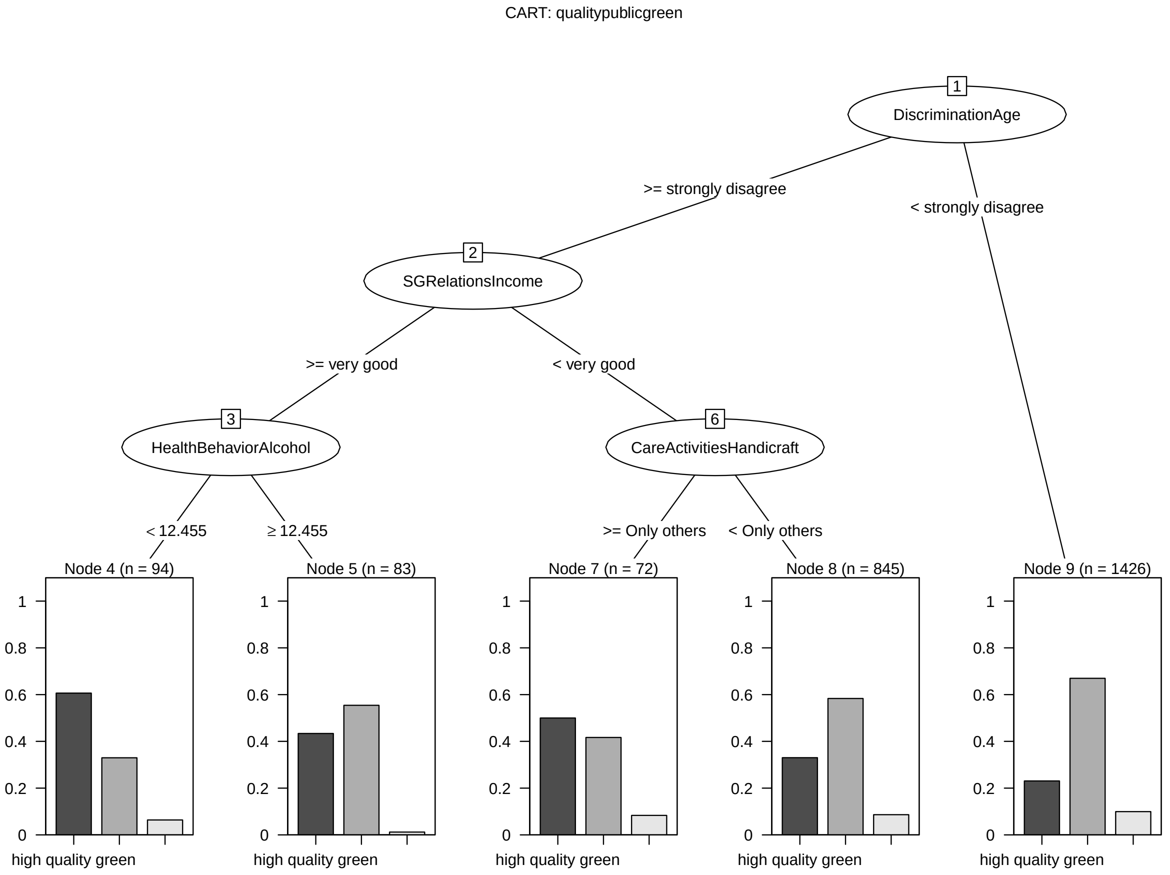

Summary

```
summary(tree_qualitypublicgreen)
```

```
## Call:
## rpart(formula = qualitypublicgreen_formula, data = KORA, method = "class",
##     parms = list(split = "Gini"), cp = 0.001, xval = 10, usesurrogate = 2,
##     minbucket = 50, maxdepth = 4)
##   n=2520 (104 observations deleted due to missingness)
##
##            CP nsplit rel error    xerror       xstd
## 1 0.008290155      0 1.0000000 1.0000000 0.02528723
## 2 0.006217617      3 0.9730570 1.0093264 0.02533125
## 3 0.001000000      4 0.9668394 0.9968912 0.02527224
##
## Variable importance
##             DiscriminationAge   DiscriminationSocialPosition
##                            30                             18
##             SGRelationsIncome          DiscriminationHeight
##                            11                             11
##          DiscriminationWeight      DiscriminationDisability
##                            10                              5
##      CareActivitiesHandicraft          HealthBehaviorAlcohol
##                             5                              5
```

```
##   SGRolesWomenWorkFamilySuffer          CareActivitiesCooking
##                              2                              1
##         CareActivitiesChildren                    SexAtBirth
##                              1                              1
##                     SGIdentity SGRelationsVocationalEducation
##                              1                              1
##
## Node number 1: 2520 observations,    complexity param=0.008290155
##   predicted class=lower quality green  expected loss=0.3829365  P(node) =1
##     class counts:   737  1555   228
##    probabilities: 0.292 0.617 0.090
##   left son=2 (1094 obs) right son=3 (1426 obs)
##   Primary splits:
##       DiscriminationAge            splits as  RRRRL, improve=22.32172, (18 missing)
##       DiscriminationSocialPosition splits as  RRRRL, improve=17.87688, (23 missing)
##       SGRelationsIncome            splits as  RRRL,  improve=15.29660, (17 missing)
##       DiscriminationWeight         splits as  RRRRL, improve=12.90878, (15 missing)
##       DiscriminationHeight         splits as  RRRRL, improve=12.59342, (15 missing)
##   Surrogate splits:
##       DiscriminationSocialPosition splits as  RRRRL, agree=0.824, adj=0.595, (3 split)
##       DiscriminationHeight         splits as  RRRRL, agree=0.721, adj=0.360, (1 split)
##       DiscriminationWeight         splits as  RRRRL, agree=0.715, adj=0.347, (0 split)
##       DiscriminationDisability     splits as  RRRRL, agree=0.643, adj=0.182, (0 split)
##       SGRolesWomenWorkFamilySuffer splits as  RRRRL, agree=0.594, adj=0.068, (12 split)
##
## Node number 2: 1094 observations,    complexity param=0.008290155
##   predicted class=lower quality green  expected loss=0.4515539  P(node) =0.434127
##     class counts:   408   600    86
##    probabilities: 0.373 0.548 0.079
##   left son=4 (177 obs) right son=5 (917 obs)
##   Primary splits:
##       SGRelationsIncome     splits as  RRRL,         improve=8.190997, (7 missing)
##       SGRelationsDisability splits as  LRRR,         improve=4.420203, (2 missing)
##       OptimismPessimism     < 7.5      to the left,  improve=3.738314, (94 missing)
##       SelfEfficacy          < 4.166667 to the right, improve=3.589856, (32 missing)
##       SGRelationsIncomeEuro < 3507.5   to the right, improve=3.042620, (80 missing)
##
## Node number 3: 1426 observations
##   predicted class=lower quality green  expected loss=0.3302945  P(node) =0.565873
##     class counts:   329   955   142
##    probabilities: 0.231 0.670 0.100
##
## Node number 4: 177 observations,    complexity param=0.008290155
##   predicted class=high quality green   expected loss=0.4745763  P(node) =0.0702381
##     class counts:    93    77     7
##    probabilities: 0.525 0.435 0.040
##   left son=8 (94 obs) right son=9 (83 obs)
##   Primary splits:
##       HealthBehaviorAlcohol           < 12.455   to the left,  improve=3.652277, (0 missing)
##       SGRelationsEmploymentCategories splits as  RLRRL,        improve=2.874881, (0 missing)
##       SGRelationsEmployment           splits as  RRLLLLLL,     improve=2.534897, (4 missing)
##       SGRolesFemMascuFeeling          splits as  LLLLLRR,      improve=2.254258, (2 missing)
##       SGRolesFemMascuWish             splits as  LLLLRRR,      improve=2.075534, (4 missing)
##   Surrogate splits:
```

```
##       CareActivitiesCooking          splits as  LLRRR,    agree=0.605, adj=0.157, (0 split)
##       SexAtBirth                     splits as  LR,       agree=0.599, adj=0.145, (0 split)
##       SGIdentity                     splits as  -L-R----, agree=0.599, adj=0.145, (0 split)
##       CareActivitiesChildren         splits as  RLRLRR,   agree=0.599, adj=0.145, (0 split)
##       SGRelationsVocationalEducation splits as  LLLLR,    agree=0.599, adj=0.145, (0 split)
##
## Node number 5: 917 observations,    complexity param=0.006217617
##   predicted class=lower quality green  expected loss=0.4296619  P(node) =0.3638889
##     class counts:   315   523    79
##    probabilities: 0.344 0.570 0.086
##   left son=10 (72 obs) right son=11 (845 obs)
##   Primary splits:
##       CareActivitiesHandicraft           splits as  RRRRL,   improve=3.742272, (10 missing)
##       SGRelationsDisability              splits as  LRRR,    improve=3.457988, (2 missing)
##       HealthBehaviorPhysicalActivity     splits as  LLLR,    improve=3.262684, (0 missing)
##       SGExpressionLooks                  splits as  RRRLLLL, improve=3.035728, (43 missing)
##       CareActivitiesAdministrativeTasks splits as  RRRLL,   improve=2.589814, (10 missing)
##
## Node number 8: 94 observations
##   predicted class=high quality green   expected loss=0.393617  P(node) =0.03730159
##     class counts:    57    31     6
##    probabilities: 0.606 0.330 0.064
##
## Node number 9: 83 observations
##   predicted class=lower quality green  expected loss=0.4457831  P(node) =0.03293651
##     class counts:    36    46     1
##    probabilities: 0.434 0.554 0.012
##
## Node number 10: 72 observations
##   predicted class=high quality green   expected loss=0.5  P(node) =0.02857143
##     class counts:    36    30     6
##    probabilities: 0.500 0.417 0.083
##
## Node number 11: 845 observations
##   predicted class=lower quality green  expected loss=0.416568  P(node) =0.3353175
##     class counts:   279   493    73
##    probabilities: 0.330 0.583 0.086
```

CIT

```r
set.seed(5678)

ctree_qualitypublicgreen = ctree(formula = qualitypublicgreen_formula,
                                 data = KORA[!is.na(KORA$qualitypublicgreen),],
                                 control = ctree_control(maxsurrogate = 5,
                                                         minbucket=50,
                                                         alpha = 0.05,
                                                         testtype = "Bonferroni",
                                                         maxdepth = 4))

plot(ctree_qualitypublicgreen,main='CIT: qualitypublicgreen',
     ep_args = list(justmin = 15),gp = gpar(fontsize = 10))
```

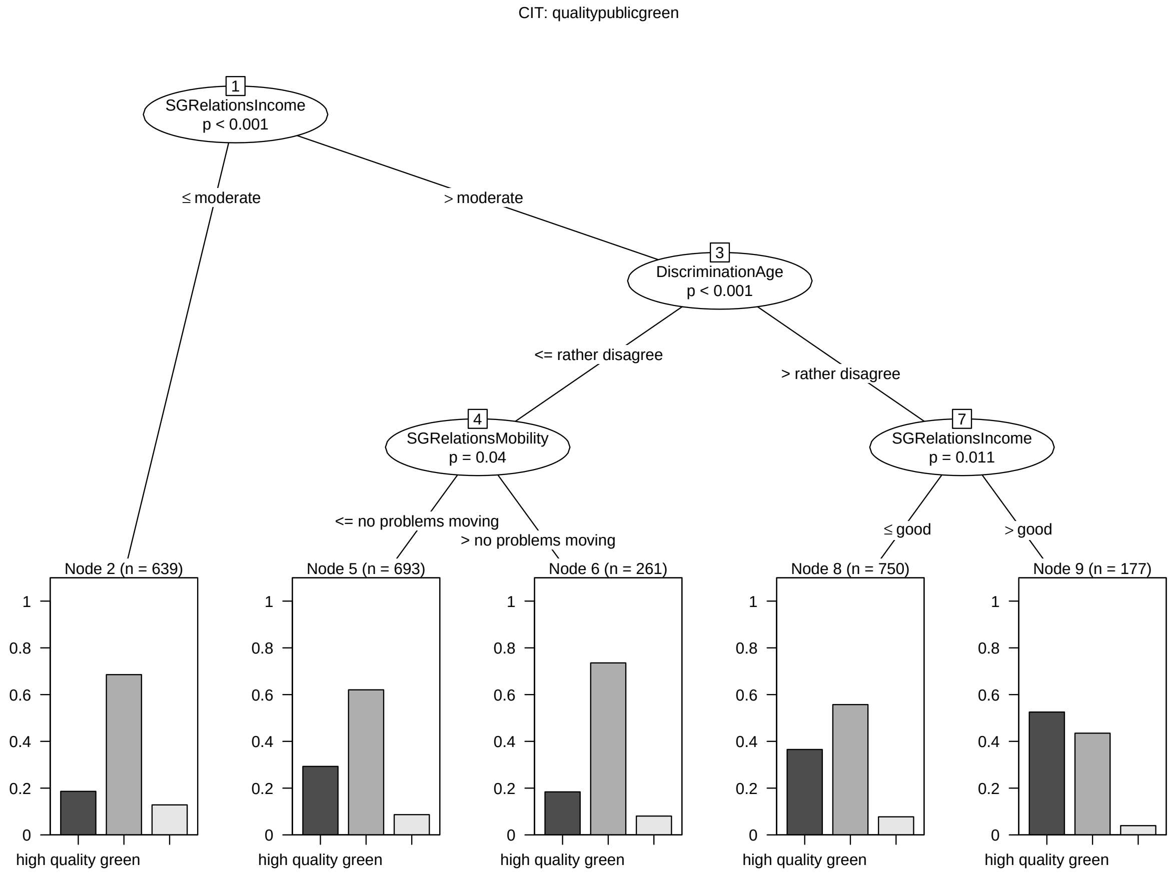

Variable importance via random forests

```
set.seed(6789)

cforest_qualitypublicgreen = cforest(formula = qualitypublicgreen_formula,
                                     data=KORA[!is.na(KORA$qualitypublicgreen),],
                                     control = ctree_control(testtype = "Univariate",
                                                             mincriterion = 0.95))

Variable_importance_qualitypublicgreen = varimp(cforest_qualitypublicgreen)
```

Variable importance plot

```
colors_qualitypublicgreen = c(rep('cornflowerblue',
                                  times = sum(Variable_importance_qualitypublicgreen <
                                        abs(min(Variable_importance_qualitypublicgreen)))),
                              rep('blue3',
                                  times = sum(Variable_importance_qualitypublicgreen >
                                        abs(min(Variable_importance_qualitypublicgreen)))))

par(mar=c(5,55,4,1)+.1)
barplot(sort(Variable_importance_qualitypublicgreen), space = 0.75,
        col = colors_qualitypublicgreen,
```

```
names.arg= rownames(Variable_importance_qualitypublicgreen),
horiz = TRUE, cex.names = 3.5, cex = 0.45,cex.axis=4, las = 1)
```

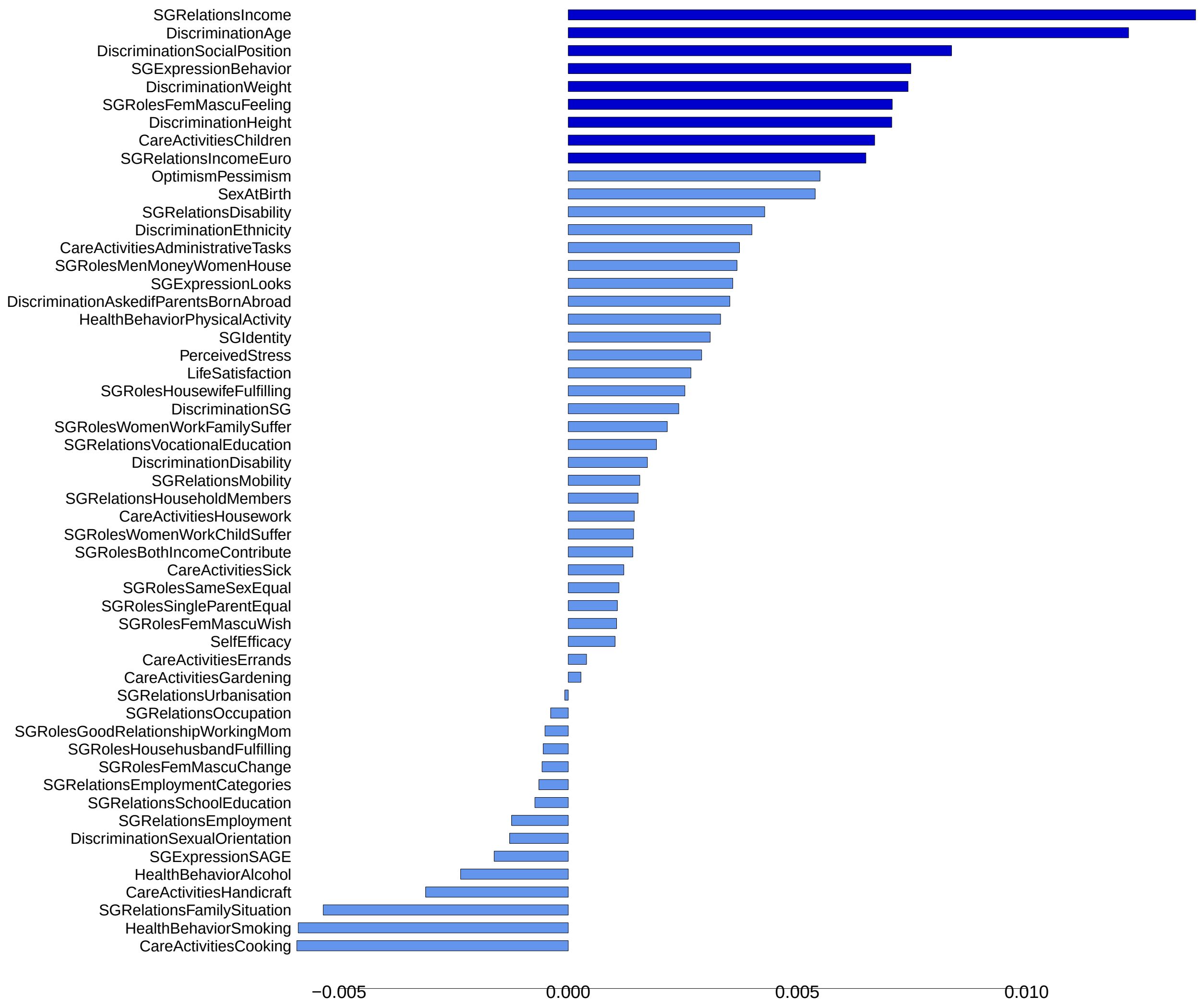

Analysis of exposure variable "greenness"

Barplot

```r
barplot(table(KORA$greenness), ylim=c(0,2500))
```

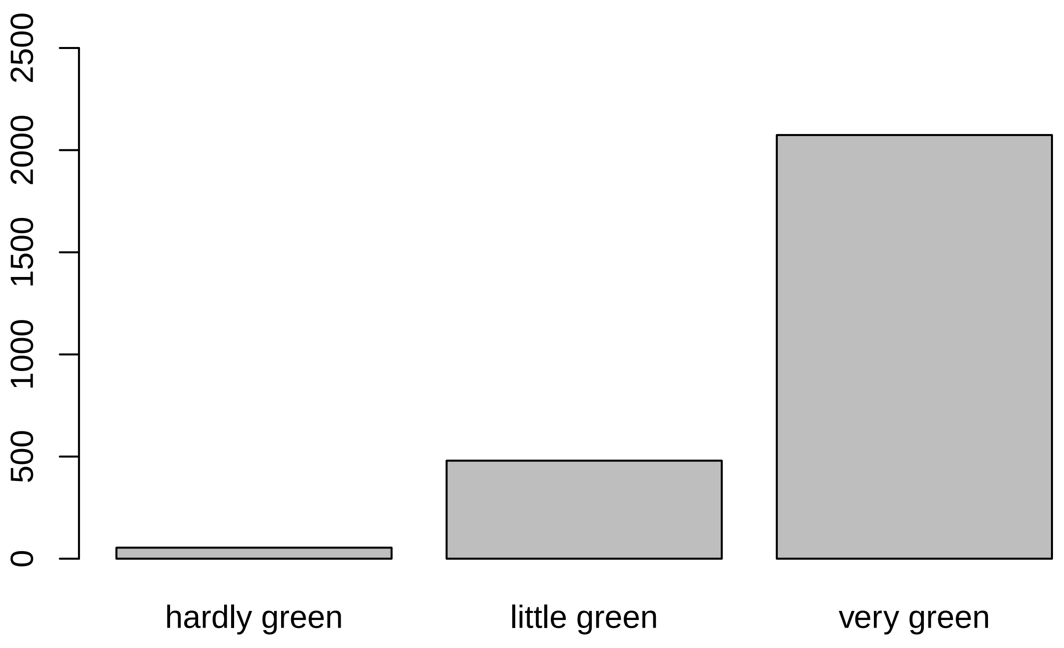

CART

```r
set.seed(7890)

greenness_formula = greenness ~ SexAtBirth +  SGIdentity +
  SGExpressionLooks + SGExpressionSAGE +
  SGExpressionBehavior + SGRolesFemMascuFeeling +
  SGRolesFemMascuWish + SGRolesFemMascuChange +
  SGRolesBothIncomeContribute + SGRolesMenMoneyWomenHouse +
  SGRolesGoodRelationshipWorkingMom + SGRolesWomenWorkChildSuffer +
  SGRolesWomenWorkFamilySuffer + SGRolesHousewifeFulfilling +
  SGRolesHousehusbandFulfilling + SGRolesSingleParentEqual +
  SGRolesSameSexEqual + CareActivitiesChildren +
  CareActivitiesSick + CareActivitiesCooking +
  CareActivitiesHousework + CareActivitiesErrands +
  CareActivitiesAdministrativeTasks +
  CareActivitiesHandicraft + CareActivitiesGardening +
  DiscriminationSocialPosition + DiscriminationAge +
  DiscriminationHeight + DiscriminationWeight +
```

```r
  DiscriminationDisability + DiscriminationEthnicity +
  DiscriminationSG + DiscriminationSexualOrientation +
  DiscriminationAskedifParentsBornAbroad + SGRelationsIncome +
  SGRelationsEmployment + SGRelationsSchoolEducation +
  SGRelationsVocationalEducation + SGRelationsFamilySituation +
  SGRelationsOccupation + SGRelationsEmploymentCategories +
  SGRelationsIncomeEuro + SGRelationsDisability +
  SGRelationsMobility + SGRelationsHouseholdMembers +
  SGRelationsUrbanisation +
  HealthBehaviorAlcohol + HealthBehaviorSmoking +
  HealthBehaviorPhysicalActivity + LifeSatisfaction +
  PerceivedStress + SelfEfficacy + OptimismPessimism

tree_greenness = rpart(formula = greenness_formula, method = 'class',
                       data = KORA, parms = list(split='Gini'), cp = 0.001,
                       xval = 10, usesurrogate = 2,
                       minbucket = 50, maxdepth = 4)

printcp(tree_greenness)
```

```
##
## Classification tree:
## rpart(formula = greenness_formula, data = KORA, method = "class",
##     parms = list(split = "Gini"), cp = 0.001, xval = 10, usesurrogate = 2,
##     minbucket = 50, maxdepth = 4)
##
## Variables actually used in tree construction:
## [1] CareActivitiesGardening      DiscriminationSocialPosition
## [3] SelfEfficacy                 SGRolesHousewifeFulfilling
##
## Root node error: 534/2608 = 0.20475
##
## n=2608 (16 observations deleted due to missingness)
##
##         CP nsplit rel error xerror     xstd
## 1 0.005618      0   1.00000 1.0000 0.038590
## 2 0.001000      4   0.97191 1.0506 0.039296
```

Pruning

```r
tree_greenness = prune(tree_greenness,cp=0.01)

plot(as.party(tree_greenness),main='CART: greenness',
     ep_args = list(justmin = 15),gp = gpar(fontsize = 10))
```

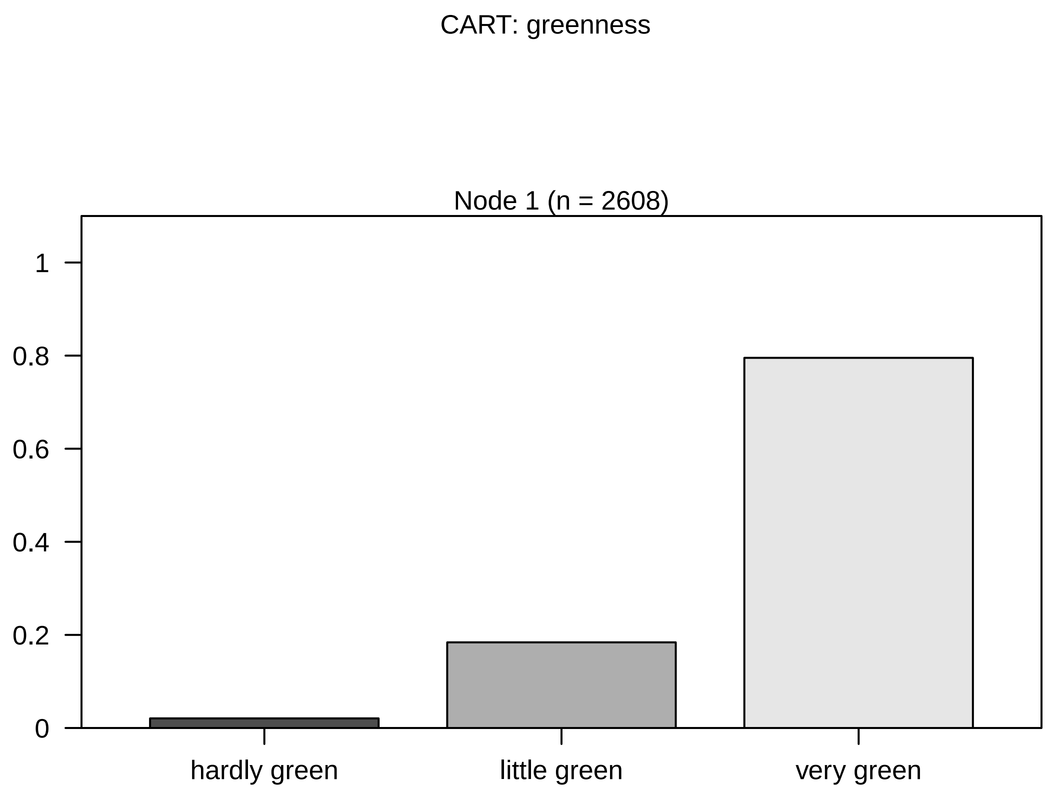

Summary

```
summary(tree_greenness)
```

```
## Call:
## rpart(formula = greenness_formula, data = KORA, method = "class",
##     parms = list(split = "Gini"), cp = 0.001, xval = 10, usesurrogate = 2,
##     minbucket = 50, maxdepth = 4)
##   n=2608 (16 observations deleted due to missingness)
##
##     CP nsplit rel error xerror       xstd
## 1 0.01      0         1      1 0.03859046
##
## Node number 1: 2608 observations
##   predicted class=very green  expected loss=0.2047546  P(node) =1
##     class counts:    54   480  2074
##    probabilities: 0.021 0.184 0.795
```

CIT

```
set.seed(8901)

ctree_greenness = ctree(formula = greenness_formula,
                        data = KORA[!is.na(KORA$greenness),],
                        control = ctree_control(maxsurrogate = 5,
```

```
                                                    minbucket = 50,
                                                    alpha = 0.05,
                                                    testtype = "Bonferroni",
                                                    maxdepth = 4))

plot(ctree_greenness,main='CIT: greenness',
     ep_args = list(justmin = 15),gp = gpar(fontsize = 10))
```

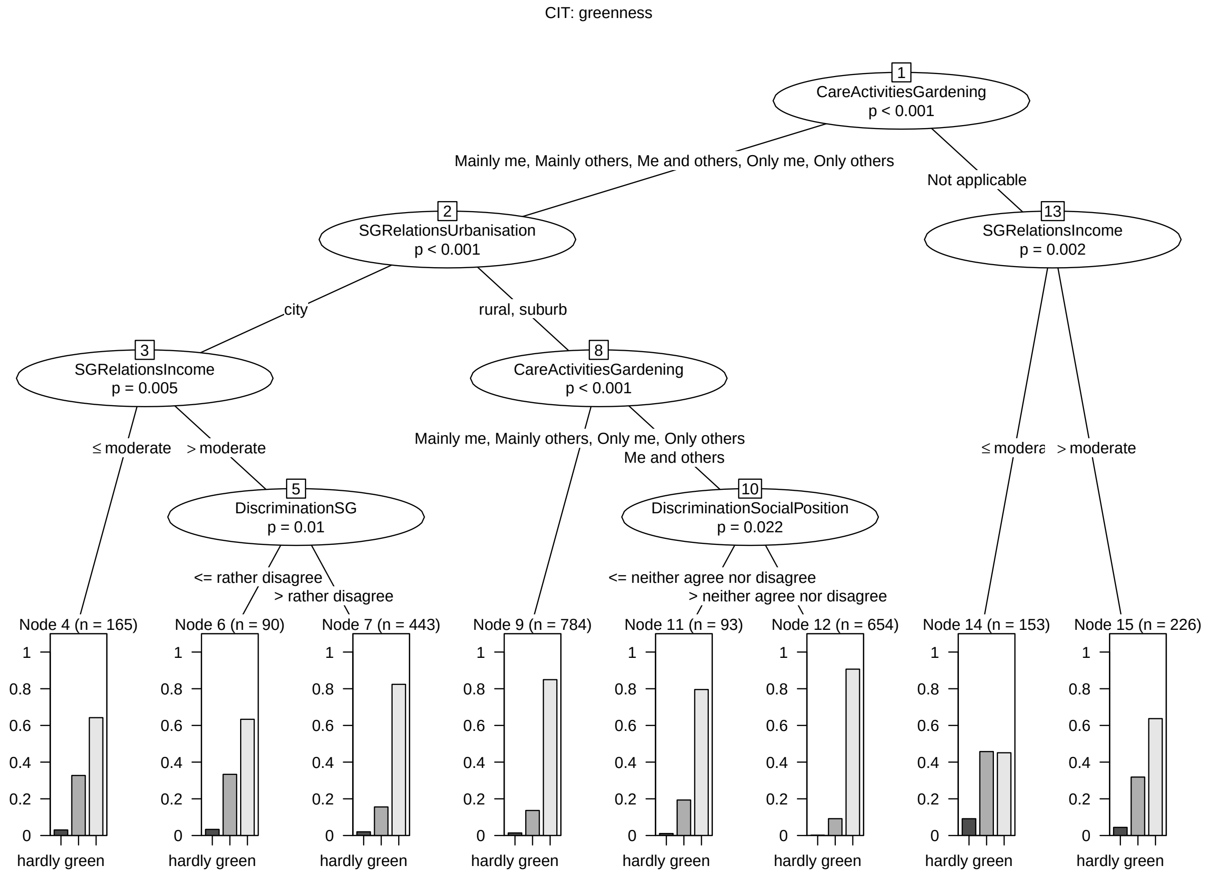

Variable importance via random forests

```
set.seed(9012)

cforest_greenness = cforest(formula = greenness_formula,
                            data=KORA[!is.na(KORA$greenness),],
                            control = ctree_control(testtype = "Univariate",
                                                    mincriterion = 0.95))
Variable_importance_greenness = varimp(cforest_greenness)
```

Variable importance plot

```
colors_greenness = c(rep('cornflowerblue',
                         times = sum(Variable_importance_greenness <
```

```r
                                    abs(min(Variable_importance_greenness)))),
                     rep('blue3',
                         times = sum(Variable_importance_greenness >
                                    abs(min(Variable_importance_greenness)))))

par(mar=c(5,55,4,1)+.1)
barplot(sort(Variable_importance_greenness), space = 0.75,
        names.arg= rownames(Variable_importance_greenness),
        col = colors_greenness,
        horiz = TRUE, cex.names = 3.5, cex = 0.45,cex.axis=4, las = 1)
```

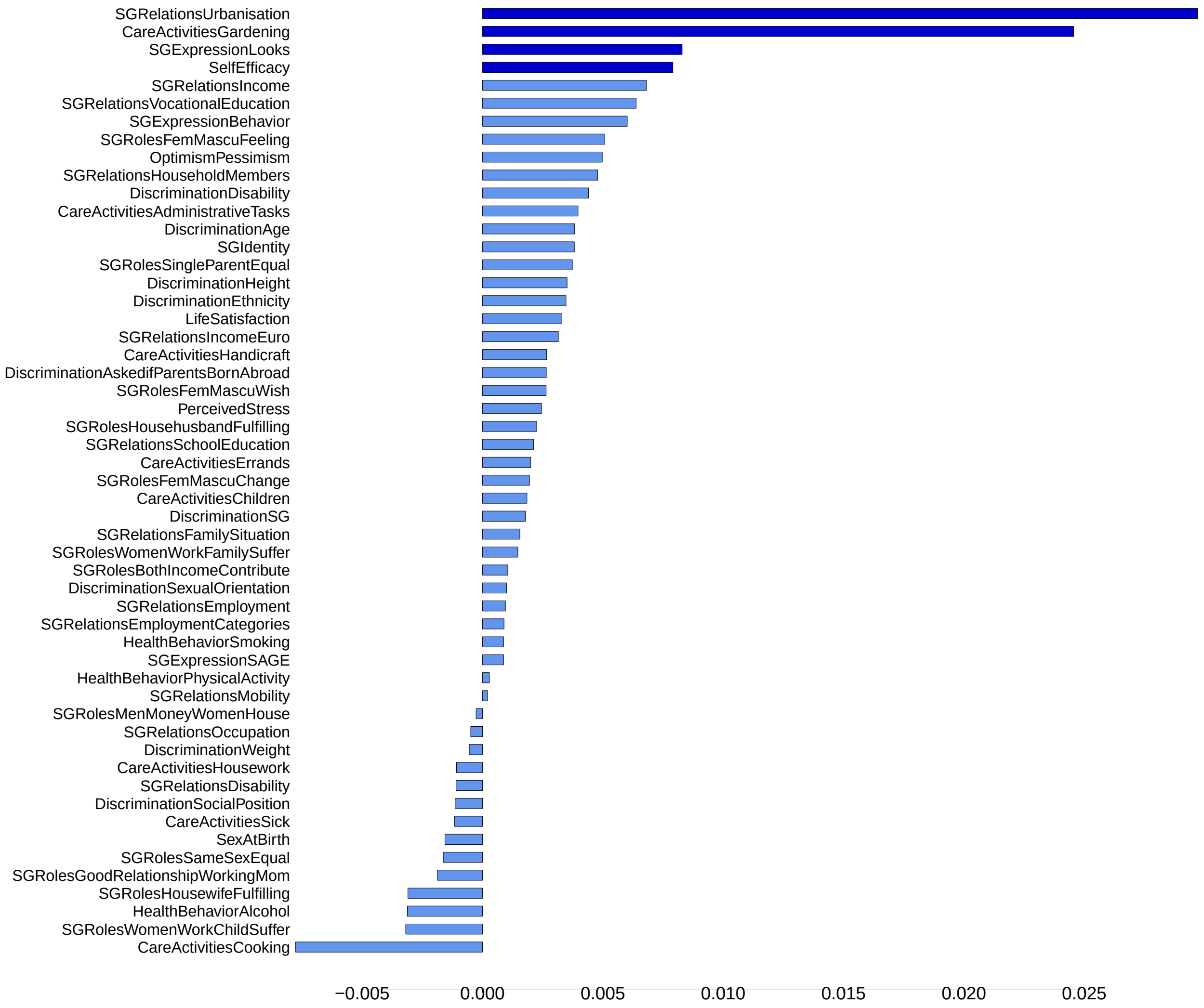

Analysis of exposure variable "NDVI_mean_300_19"

Density plot

```
ggdensity(KORA$NDVI_mean_300_19, xlab='NDVI_mean_300_19')
```

```
## Warning: Removed 10 rows containing non-finite values (stat_density).
```

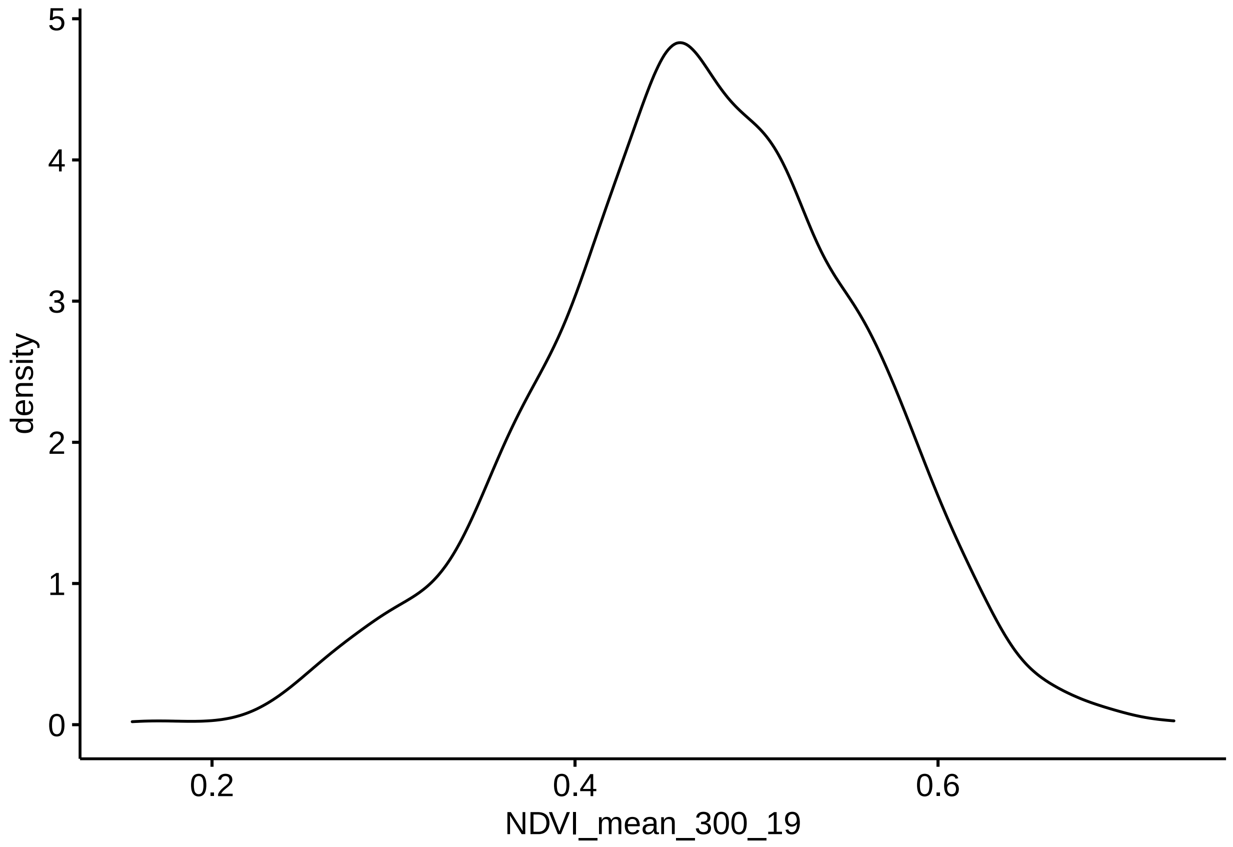

CART

```
set.seed(12345)

NDVI_mean_300_19_formula = NDVI_mean_300_19 ~ SexAtBirth +  SGIdentity +
  SGExpressionLooks + SGExpressionSAGE +
  SGExpressionBehavior + SGRolesFemMascuFeeling +
  SGRolesFemMascuWish + SGRolesFemMascuChange +
  SGRolesBothIncomeContribute + SGRolesMenMoneyWomenHouse +
  SGRolesGoodRelationshipWorkingMom + SGRolesWomenWorkChildSuffer +
  SGRolesWomenWorkFamilySuffer + SGRolesHousewifeFulfilling +
  SGRolesHousehusbandFulfilling + SGRolesSingleParentEqual +
  SGRolesSameSexEqual + CareActivitiesChildren +
  CareActivitiesSick + CareActivitiesCooking +
  CareActivitiesHousework + CareActivitiesErrands +
  CareActivitiesAdministrativeTasks +
  CareActivitiesHandicraft + CareActivitiesGardening +
  DiscriminationSocialPosition + DiscriminationAge +
```

```r
  DiscriminationHeight + DiscriminationWeight +
  DiscriminationDisability + DiscriminationEthnicity +
  DiscriminationSG + DiscriminationSexualOrientation +
  DiscriminationAskedifParentsBornAbroad + SGRelationsIncome +
  SGRelationsEmployment + SGRelationsSchoolEducation +
  SGRelationsVocationalEducation + SGRelationsFamilySituation +
  SGRelationsOccupation + SGRelationsEmploymentCategories +
  SGRelationsIncomeEuro + SGRelationsDisability +
  SGRelationsMobility + SGRelationsHouseholdMembers +
  SGRelationsUrbanisation +
  HealthBehaviorAlcohol + HealthBehaviorSmoking +
  HealthBehaviorPhysicalActivity + LifeSatisfaction +
  PerceivedStress + SelfEfficacy + OptimismPessimism

tree_NDVI_mean_300_19 = rpart(formula = NDVI_mean_300_19_formula,
                              data = KORA, method = 'anova', cp = 0.001,
                              xval = 10, usesurrogate = 2,
                              minbucket = 50, maxdepth = 4)

printcp(tree_NDVI_mean_300_19)
```

```
##
## Regression tree:
## rpart(formula = NDVI_mean_300_19_formula, data = KORA, method = "anova",
##     cp = 0.001, xval = 10, usesurrogate = 2, minbucket = 50,
##     maxdepth = 4)
##
## Variables actually used in tree construction:
## [1] CareActivitiesGardening     OptimismPessimism
## [3] SGRelationsIncomeEuro       SGRelationsUrbanisation
## [5] SGRolesSingleParentEqual    SGRolesWomenWorkChildSuffer
##
## Root node error: 19.494/2614 = 0.0074573
##
## n=2614 (10 observations deleted due to missingness)
##
##          CP nsplit rel error  xerror     xstd
## 1 0.2459865      0   1.00000 1.00055 0.027406
## 2 0.0660772      1   0.75401 0.75498 0.020571
## 3 0.0140831      2   0.68794 0.68909 0.019391
## 4 0.0042616      3   0.67385 0.67543 0.019236
## 5 0.0028020      4   0.66959 0.67773 0.019289
## 6 0.0024646      5   0.66679 0.68260 0.019464
## 7 0.0019334      6   0.66432 0.68602 0.019508
## 8 0.0017906      7   0.66239 0.68683 0.019529
## 9 0.0010000      8   0.66060 0.69134 0.019703
```

Pruning

```r
tree_NDVI_mean_300_19 = prune(tree_NDVI_mean_300_19,cp=0.005)

plot(as.party(tree_NDVI_mean_300_19),main='CART: NDVI_mean_300_19',
     ep_args = list(justmin = 15),gp = gpar(fontsize = 10))
```

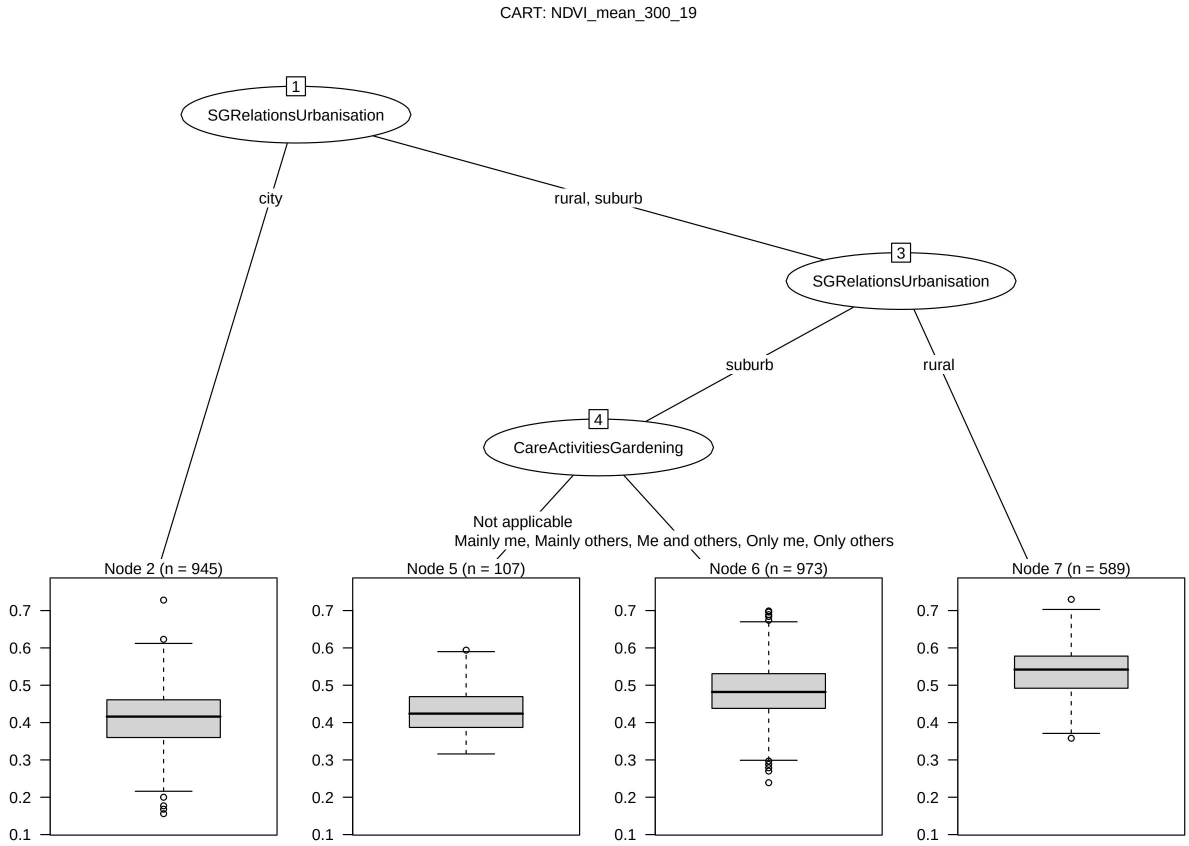

Summary

```
summary(tree_NDVI_mean_300_19)
```

```
## Call:
## rpart(formula = NDVI_mean_300_19_formula, data = KORA, method = "anova",
##     cp = 0.001, xval = 10, usesurrogate = 2, minbucket = 50,
##     maxdepth = 4)
##   n=2614 (10 observations deleted due to missingness)
##
##           CP nsplit rel error    xerror       xstd
## 1 0.24598654      0 1.0000000 1.0005505 0.02740587
## 2 0.06607718      1 0.7540135 0.7549815 0.02057066
## 3 0.01408309      2 0.6879363 0.6890869 0.01939129
## 4 0.00500000      3 0.6738532 0.6754270 0.01923623
##
## Variable importance
##     SGRelationsUrbanisation     CareActivitiesGardening
##                          87                          12
## SGRelationsHouseholdMembers
##                           1
##
## Node number 1: 2614 observations,    complexity param=0.2459865
```

```
##   mean=0.4677395, MSE=0.007457347
##   left son=2 (945 obs) right son=3 (1669 obs)
##   Primary splits:
##       SGRelationsUrbanisation     splits as  LRR,        improve=0.24598650, (0 missing)
##       CareActivitiesGardening     splits as  RRRLRR,     improve=0.06436882, (24 missing)
##       SGRelationsHouseholdMembers < 2.5    to the left,  improve=0.01939952, (2 missing)
##       SGRelationsFamilySituation  splits as  LR,         improve=0.01582301, (0 missing)
##       CareActivitiesChildren      splits as  RRRLLL,     improve=0.01565761, (20 missing)
##   Surrogate splits:
##       CareActivitiesGardening     splits as  RRRLRR,     agree=0.679, adj=0.111, (0 split)
##       SGRelationsHouseholdMembers < 1.5    to the left,  agree=0.642, adj=0.008, (0 split)
##       HealthBehaviorAlcohol       < 101.43 to the right, agree=0.640, adj=0.004, (0 split)
##
## Node number 2: 945 observations
##   mean=0.4108201, MSE=0.006017654
##
## Node number 3: 1669 observations,    complexity param=0.06607718
##   mean=0.4999676, MSE=0.00539945
##   left son=6 (1080 obs) right son=7 (589 obs)
##   Primary splits:
##       SGRelationsUrbanisation     splits as  -RL,        improve=0.142934000, (0 missing)
##       CareActivitiesGardening     splits as  RRRLRR,     improve=0.042836200, (14 missing)
##       SGRelationsHouseholdMembers < 2.5    to the left,  improve=0.010372460, (1 missing)
##       SGRelationsFamilySituation  splits as  LR,         improve=0.006794333, (0 missing)
##       CareActivitiesChildren      splits as  RRRLLL,     improve=0.006389323, (13 missing)
##   Surrogate splits:
##       SGRelationsHouseholdMembers < 4.5    to the left,  agree=0.655, adj=0.024, (0 split)
##       HealthBehaviorAlcohol       < 92.215 to the left,  agree=0.649, adj=0.007, (0 split)
##       HealthBehaviorSmoking       splits as  LRLL,       agree=0.649, adj=0.007, (0 split)
##       DiscriminationWeight        splits as  RRLLL,      agree=0.649, adj=0.005, (0 split)
##
## Node number 6: 1080 observations,    complexity param=0.01408309
##   mean=0.4794519, MSE=0.005167703
##   left son=12 (107 obs) right son=13 (973 obs)
##   Primary splits:
##       CareActivitiesGardening    splits as  RRRLRR,     improve=0.049286950, (12 missing)
##       SGRelationsIncomeEuro      < 1259.5 to the left,  improve=0.014245380, (71 missing)
##       SGRelationsIncome          splits as  LLLR,       improve=0.008697215, (11 missing)
##       SGRelationsFamilySituation splits as  LR,         improve=0.008078304, (0 missing)
##       SGRelationsDisability      splits as  RLLL,       improve=0.007708916, (1 missing)
##
## Node number 7: 589 observations
##   mean=0.5375857, MSE=0.003637499
##
## Node number 12: 107 observations
##   mean=0.4313738, MSE=0.003459692
##
## Node number 13: 973 observations
##   mean=0.484739, MSE=0.005073385
```

CIT

```
set.seed(23456)

ctree_NDVI_mean_300_19 = ctree(formula = NDVI_mean_300_19_formula,
                               data = KORA[!is.na(KORA$NDVI_mean_300_19),],
                               control = ctree_control(maxsurrogate = 5,
                                                       alpha = 0.05,
                                                       minbucket=50,
                                                       testtype = "Bonferroni",
                                                       maxdepth = 4))

plot(ctree_NDVI_mean_300_19, main='CIT: NDVI_mean_300_19',
     ep_args = list(justmin = 15),gp = gpar(fontsize = 10))
```

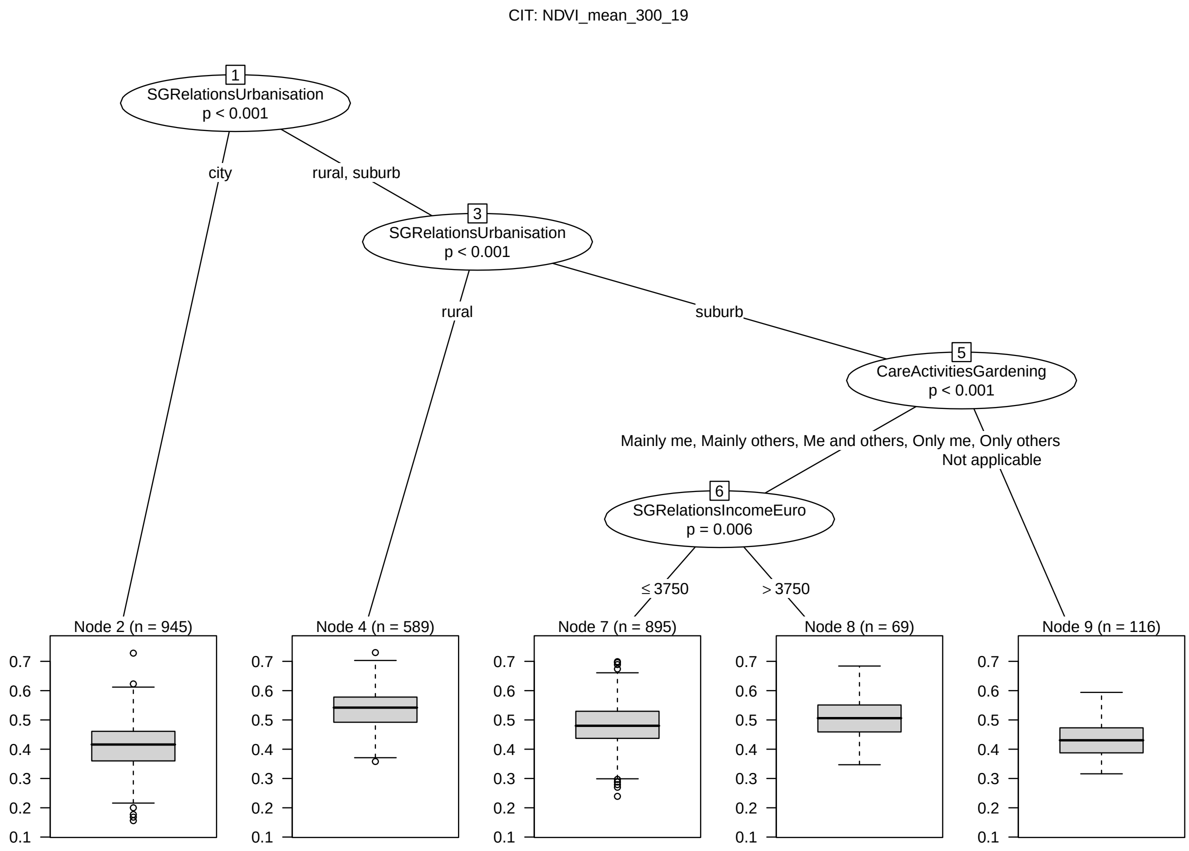

Variable importance via random forests

```
set.seed(34567)

cforest_NDVI_mean_300_19 = cforest(formula = NDVI_mean_300_19_formula,
                                    data=KORA[!is.na(KORA$NDVI_mean_300_19),],
                                   control = ctree_control(testtype = "Univariate",
                                                           mincriterion = 0.95))

Variable_importance_NDVI_mean_300_19 = varimp(cforest_NDVI_mean_300_19)
```

Variable importance plot

```
colors_NDVI_mean_300_19 = c(rep('cornflowerblue',
                               times = sum(Variable_importance_NDVI_mean_300_19 <
                                       abs(min(Variable_importance_NDVI_mean_300_19)))),
                            rep('blue3',
                               times = sum(Variable_importance_NDVI_mean_300_19 >
                                       abs(min(Variable_importance_NDVI_mean_300_19)))))

par(mar=c(5,55,4,1)+.1)
barplot(sort(Variable_importance_NDVI_mean_300_19), space = 0.75,
        col = colors_NDVI_mean_300_19,
        names.arg= rownames(Variable_importance_NDVI_mean_300_19),
        horiz = TRUE, cex.names = 3.5, cex = 0.45,cex.axis=4, las = 1)
```

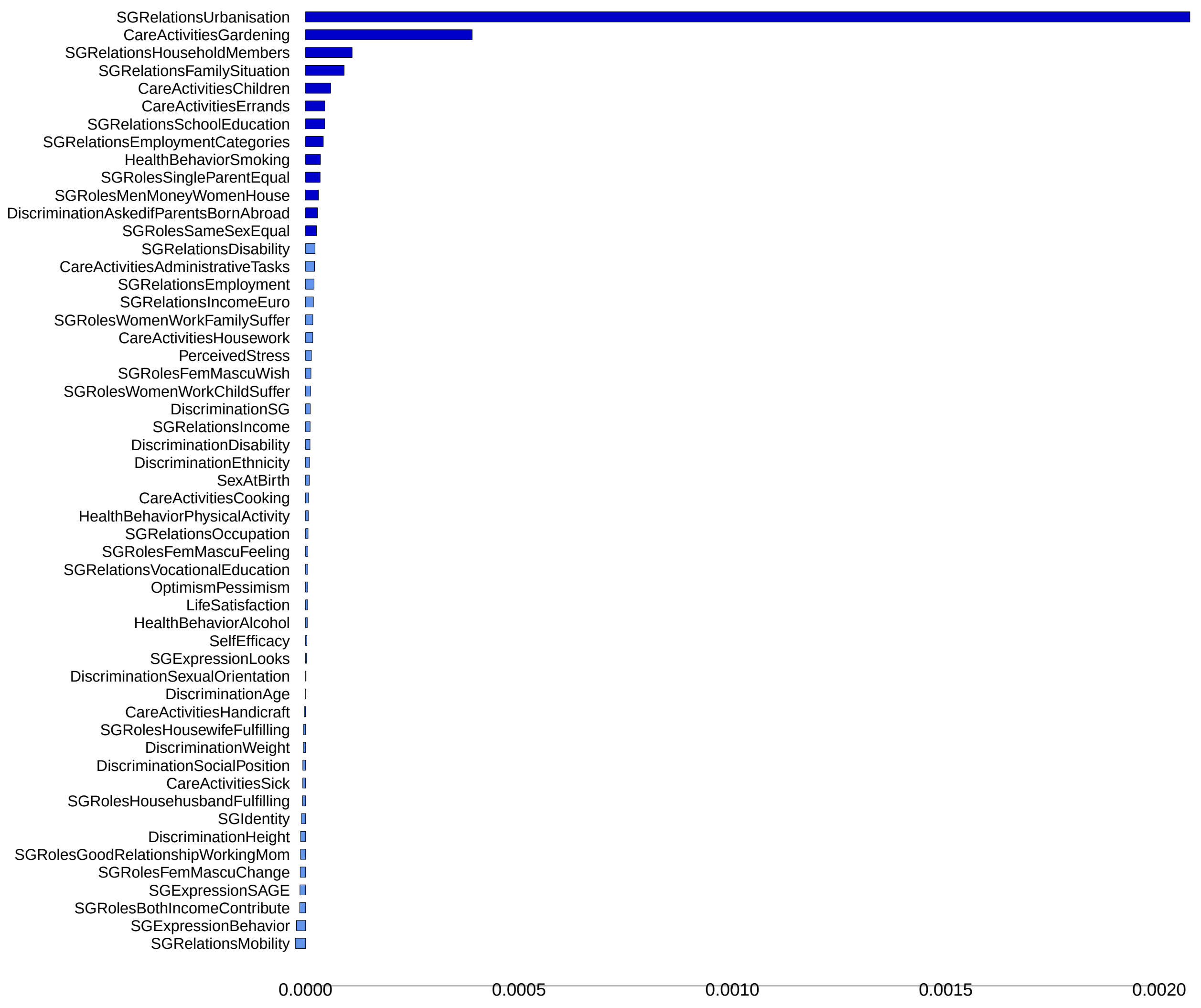

Analysis of exposure variable "NDVI_mean_1000_19"

Density plot

```
ggdensity(KORA$NDVI_mean_1000_19, xlab='NDVI_mean_1000_19')
```

```
## Warning: Removed 10 rows containing non-finite values (stat_density).
```

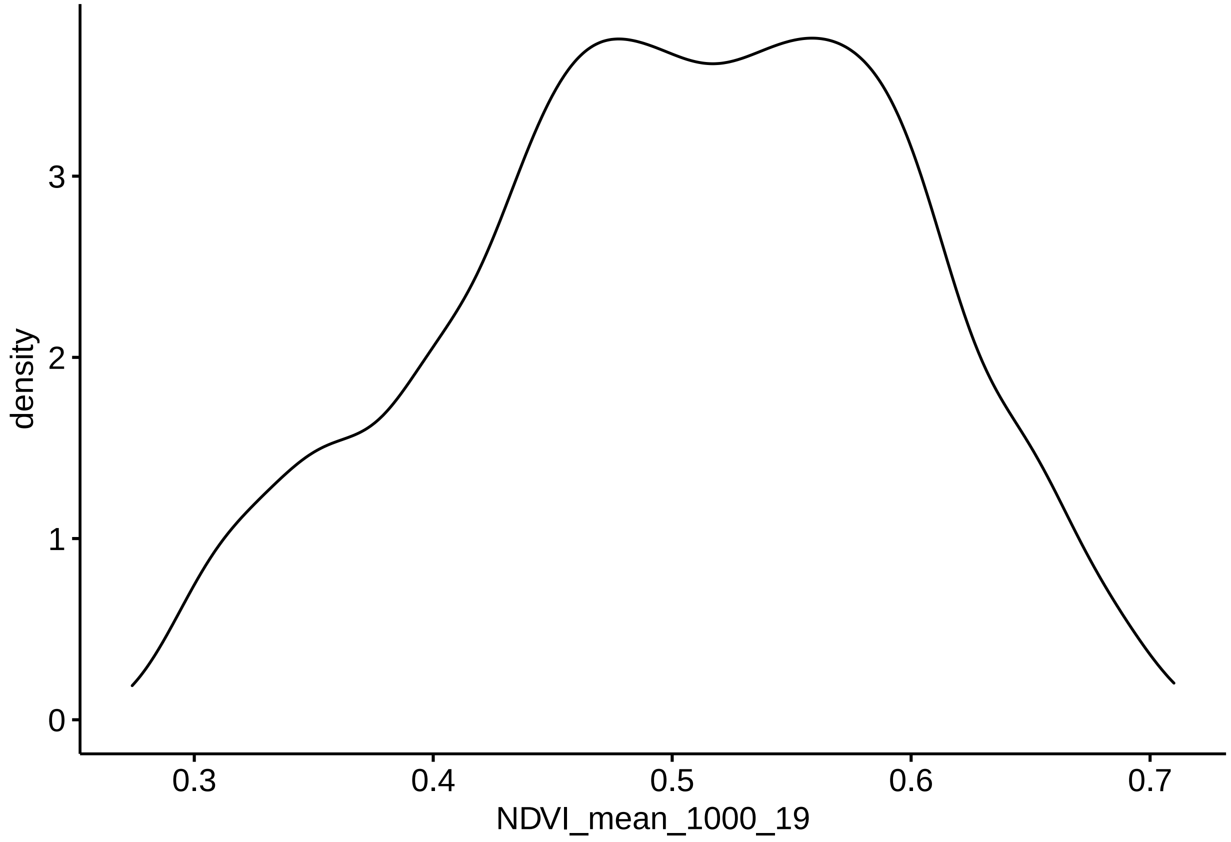

CART

```
set.seed(45678)

NDVI_mean_1000_19_formula = NDVI_mean_1000_19 ~ SexAtBirth +  SGIdentity +
  SGExpressionLooks + SGExpressionSAGE +
  SGExpressionBehavior + SGRolesFemMascuFeeling +
  SGRolesFemMascuWish + SGRolesFemMascuChange +
  SGRolesBothIncomeContribute + SGRolesMenMoneyWomenHouse +
  SGRolesGoodRelationshipWorkingMom + SGRolesWomenWorkChildSuffer +
  SGRolesWomenWorkFamilySuffer + SGRolesHousewifeFulfilling +
  SGRolesHousehusbandFulfilling + SGRolesSingleParentEqual +
  SGRolesSameSexEqual + CareActivitiesChildren +
  CareActivitiesSick + CareActivitiesCooking +
  CareActivitiesHousework + CareActivitiesErrands +
  CareActivitiesAdministrativeTasks +
  CareActivitiesHandicraft + CareActivitiesGardening +
  DiscriminationSocialPosition + DiscriminationAge +
```

```
  DiscriminationHeight + DiscriminationWeight +
  DiscriminationDisability + DiscriminationEthnicity +
  DiscriminationSG + DiscriminationSexualOrientation +
  DiscriminationAskedifParentsBornAbroad + SGRelationsIncome +
  SGRelationsEmployment + SGRelationsSchoolEducation +
  SGRelationsVocationalEducation + SGRelationsFamilySituation +
  SGRelationsOccupation + SGRelationsEmploymentCategories +
  SGRelationsIncomeEuro + SGRelationsDisability +
  SGRelationsMobility + SGRelationsHouseholdMembers +
  SGRelationsUrbanisation +
  HealthBehaviorAlcohol + HealthBehaviorSmoking +
  HealthBehaviorPhysicalActivity + LifeSatisfaction +
  PerceivedStress + SelfEfficacy + OptimismPessimism

tree_NDVI_mean_1000_19 = rpart(formula = NDVI_mean_1000_19_formula,
                               data = KORA, method = 'anova', cp = 0.001,
                               xval = 10, usesurrogate = 2,
                               minbucket = 50, maxdepth = 4)

printcp(tree_NDVI_mean_1000_19)
```

```
##
## Regression tree:
## rpart(formula = NDVI_mean_1000_19_formula, data = KORA, method = "anova",
##     cp = 0.001, xval = 10, usesurrogate = 2, minbucket = 50,
##     maxdepth = 4)
##
## Variables actually used in tree construction:
## [1] CareActivitiesGardening       SGRelationsUrbanisation
## [3] SGRolesHousehusbandFulfilling SGRolesSameSexEqual
## [5] SGRolesSingleParentEqual      SGRolesWomenWorkChildSuffer
##
## Root node error: 22.615/2614 = 0.0086516
##
## n=2614 (10 observations deleted due to missingness)
##
##          CP nsplit rel error  xerror     xstd
## 1 0.3644926      0   1.00000 1.00114 0.022872
## 2 0.0732556      1   0.63551 0.63729 0.015271
## 3 0.0123146      2   0.56225 0.56391 0.014378
## 4 0.0055100      3   0.54994 0.55361 0.014273
## 5 0.0025819      4   0.54443 0.55000 0.014184
## 6 0.0023891      5   0.54185 0.55672 0.014418
## 7 0.0018748      6   0.53946 0.56197 0.014597
## 8 0.0017234      7   0.53758 0.56123 0.014599
## 9 0.0010000      8   0.53586 0.56148 0.014642
```

Pruning

```
tree_NDVI_mean_1000_19 = prune(tree_NDVI_mean_1000_19,cp=0.004)

plot(as.party(tree_NDVI_mean_1000_19),main='CART: NDVI_mean_1000_19',
     ep_args = list(justmin = 15),gp = gpar(fontsize = 10))
```

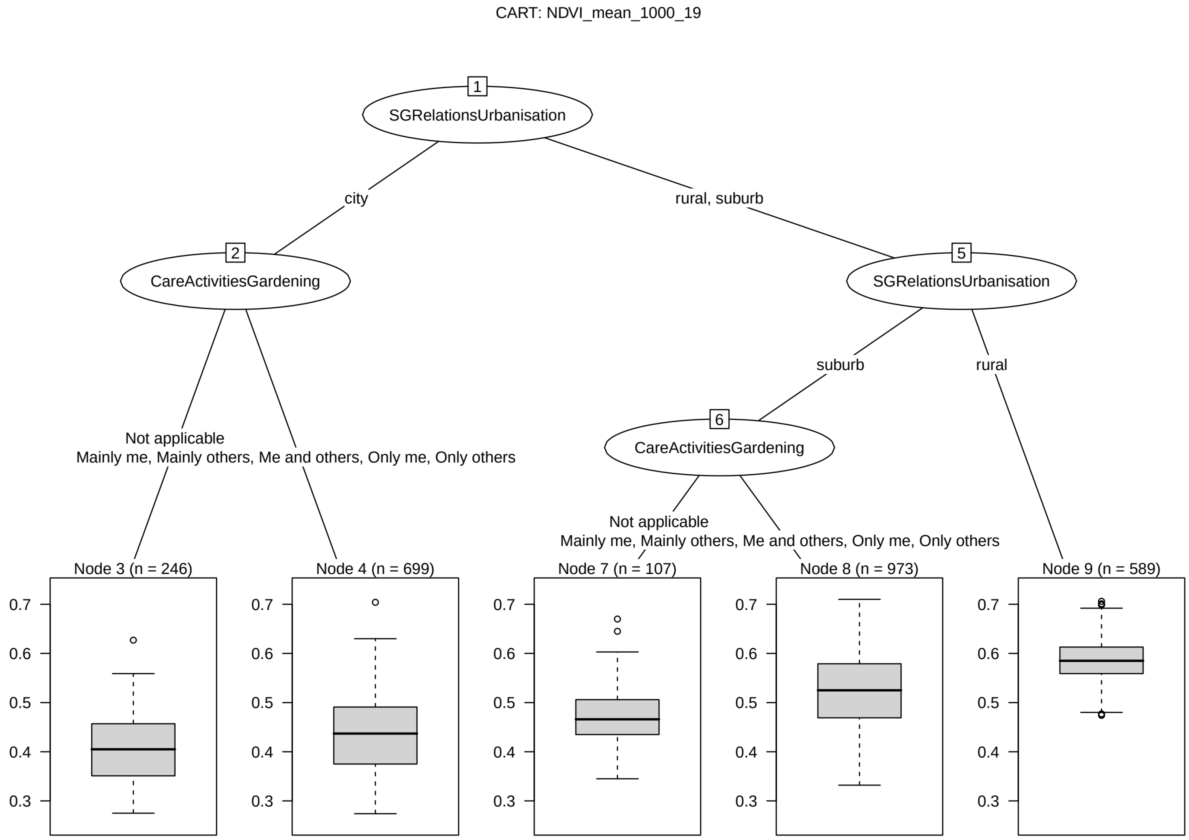

Summary

```
summary(tree_NDVI_mean_1000_19)
```

```
## Call:
## rpart(formula = NDVI_mean_1000_19_formula, data = KORA, method = "anova",
##     cp = 0.001, xval = 10, usesurrogate = 2, minbucket = 50,
##     maxdepth = 4)
##   n=2614 (10 observations deleted due to missingness)
##
##            CP nsplit rel error    xerror       xstd
## 1 0.364492609      0 1.0000000 1.0011431 0.02287164
## 2 0.073255609      1 0.6355074 0.6372880 0.01527082
## 3 0.012314624      2 0.5622518 0.5639098 0.01437780
## 4 0.005510017      3 0.5499372 0.5536053 0.01427264
## 5 0.004000000      4 0.5444271 0.5500034 0.01418383
##
## Variable importance
##     SGRelationsUrbanisation     CareActivitiesGardening
##                          87                          12
## SGRelationsHouseholdMembers
##                           1
##
```

```
## Node number 1: 2614 observations,    complexity param=0.3644926
##   mean=0.5025015, MSE=0.008651598
##   left son=2 (945 obs) right son=3 (1669 obs)
##   Primary splits:
##       SGRelationsUrbanisation     splits as  LRR,        improve=0.36449260, (0 missing)
##       CareActivitiesGardening     splits as  RRRLRR,     improve=0.08475616, (24 missing)
##       SGRelationsHouseholdMembers < 1.5    to the left,  improve=0.02436468, (2 missing)
##       SGRelationsFamilySituation  splits as  LR,         improve=0.02117868, (0 missing)
##       CareActivitiesChildren      splits as  RRRLLL,     improve=0.01959434, (20 missing)
##   Surrogate splits:
##       CareActivitiesGardening     splits as  RRRLRR,     agree=0.679, adj=0.111, (0 split)
##       SGRelationsHouseholdMembers < 1.5    to the left,  agree=0.642, adj=0.008, (0 split)
##       HealthBehaviorAlcohol       < 101.43 to the right, agree=0.640, adj=0.004, (0 split)
##
## Node number 2: 945 observations,    complexity param=0.005510017
##   mean=0.427873, MSE=0.005866469
##   left son=4 (246 obs) right son=5 (699 obs)
##   Primary splits:
##       CareActivitiesGardening     splits as  RRRLRR,     improve=0.02443696, (10 missing)
##       SGRolesSingleParentEqual    splits as  LLLLR,      improve=0.02009378, (7 missing)
##       SGRelationsHouseholdMembers < 1.5    to the left,  improve=0.01334523, (1 missing)
##       SGRolesSameSexEqual         splits as  LLLRR,      improve=0.01267848, (10 missing)
##       SGRelationsIncome           splits as  LLRR,       improve=0.01253188, (12 missing)
##   Surrogate splits:
##       CareActivitiesErrands   splits as  RRRRL, agree=0.74, adj=0.008, (2 split)
##       DiscriminationEthnicity splits as  LLRRR, agree=0.74, adj=0.008, (7 split)
##
## Node number 3: 1669 observations,    complexity param=0.07325561
##   mean=0.5447567, MSE=0.005289612
##   left son=6 (1080 obs) right son=7 (589 obs)
##   Primary splits:
##       SGRelationsUrbanisation     splits as  -RL,        improve=0.187656100, (0 missing)
##       CareActivitiesGardening     splits as  RRRLRR,     improve=0.044903760, (14 missing)
##       SGRelationsHouseholdMembers < 2.5    to the left,  improve=0.008953224, (1 missing)
##       HealthBehaviorSmoking       splits as  LLRL,       improve=0.007131696, (1 missing)
##       SGRolesWomenWorkChildSuffer splits as  RLLLL,      improve=0.007123915, (16 missing)
##   Surrogate splits:
##       SGRelationsHouseholdMembers < 4.5    to the left,  agree=0.655, adj=0.024, (0 split)
##       HealthBehaviorAlcohol       < 92.215 to the left,  agree=0.649, adj=0.007, (0 split)
##       HealthBehaviorSmoking       splits as  LRLL,       agree=0.649, adj=0.007, (0 split)
##       DiscriminationWeight        splits as  RRLLL,      agree=0.649, adj=0.005, (0 split)
##
## Node number 4: 246 observations
##   mean=0.4085163, MSE=0.004873209
##
## Node number 5: 699 observations
##   mean=0.4346853, MSE=0.006037758
##
## Node number 6: 1080 observations,    complexity param=0.01231462
##   mean=0.5214898, MSE=0.005454563
##   left son=12 (107 obs) right son=13 (973 obs)
##   Primary splits:
##       CareActivitiesGardening         splits as  RRRLRR, improve=0.047409200, (12 missing)
##       SGRelationsDisability           splits as  RLLL,   improve=0.011278340, (1 missing)
```

```
##       SGRolesHousehusbandFulfilling   splits as  LLLRR,  improve=0.007937513, (19 missing)
##       HealthBehaviorSmoking           splits as  LLRR,   improve=0.007157206, (1 missing)
##       DiscriminationSexualOrientation splits as  LLLLR,  improve=0.006191136, (15 missing)
##
## Node number 7: 589 observations
##   mean=0.5874194, MSE=0.00217443
##
## Node number 12: 107 observations
##   mean=0.4730654, MSE=0.003709762
##
## Node number 13: 973 observations
##   mean=0.526815, MSE=0.00536021
```

CIT

```
set.seed(56789)

ctree_NDVI_mean_1000_19 = ctree(formula = NDVI_mean_1000_19_formula,
                                data = KORA[!is.na(KORA$NDVI_mean_1000_19),],
                                control = ctree_control(maxsurrogate = 5,
                                                        alpha = 0.05,
                                                        minbucket=50,
                                                        testtype = "Bonferroni",
                                                        maxdepth = 4))

plot(ctree_NDVI_mean_1000_19,main='CIT: NDVI_mean_1000_19',
     ep_args = list(justmin = 15),gp = gpar(fontsize = 10))
```

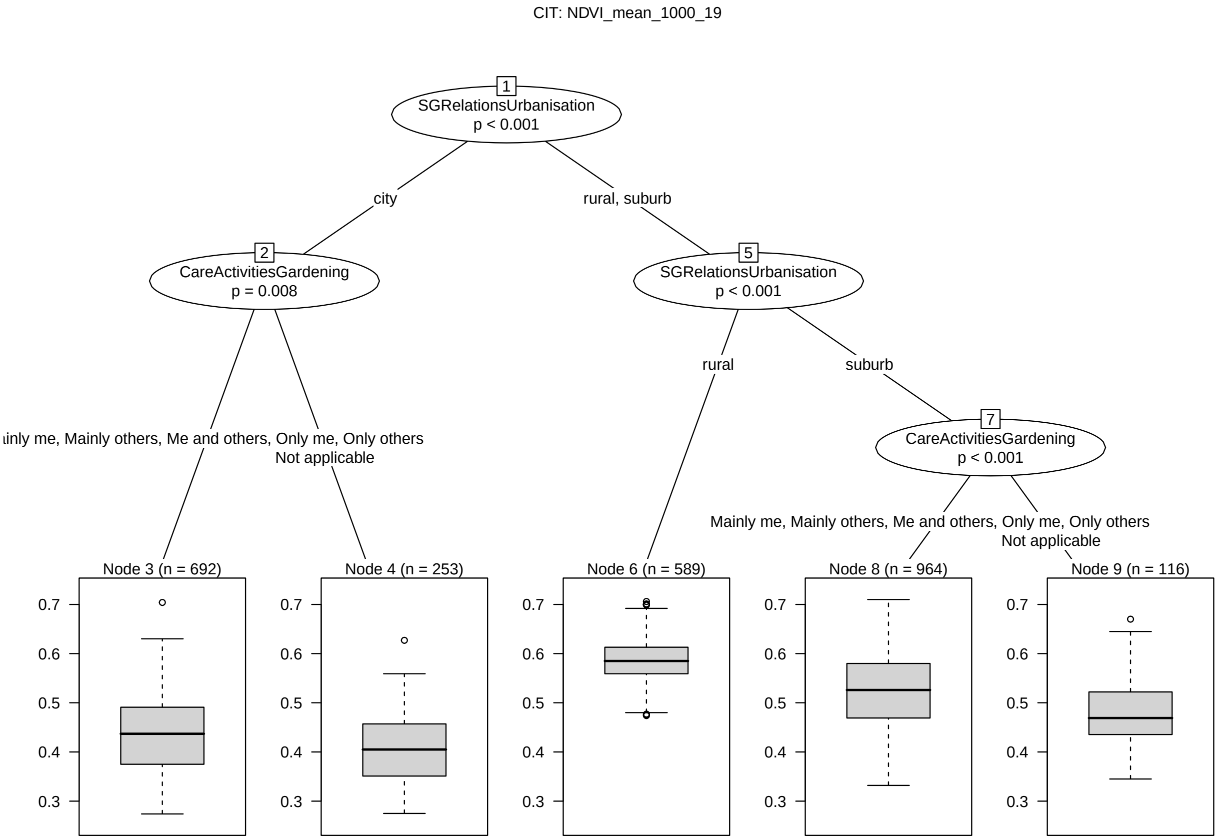

Variable importance via random forests

```
set.seed(67890)

cforest_NDVI_mean_1000_19 = cforest(formula = NDVI_mean_1000_19_formula,
                                    data=KORA[!is.na(KORA$NDVI_mean_1000_19),],
                                   control = ctree_control(testtype = "Univariate",
                                                           mincriterion = 0.95))

Variable_importance_NDVI_mean_1000_19 = varimp(cforest_NDVI_mean_1000_19)
```

Variable importance plot

```
colors_NDVI_mean_1000_19 = c(rep('cornflowerblue',
                                times = sum(Variable_importance_NDVI_mean_1000_19 <
                                       abs(min(Variable_importance_NDVI_mean_1000_19)))),
                            rep('blue3',
                                times = sum(Variable_importance_NDVI_mean_1000_19 >
                                       abs(min(Variable_importance_NDVI_mean_1000_19)))))

par(mar=c(5,55,4,1)+.1)
barplot(sort(Variable_importance_NDVI_mean_1000_19), space = 0.75,
        col = colors_NDVI_mean_1000_19,
```

```
        names.arg= rownames(Variable_importance_NDVI_mean_1000_19),
        horiz = TRUE, cex.names = 3.5, cex = 0.45,cex.axis=4, las = 1)
```

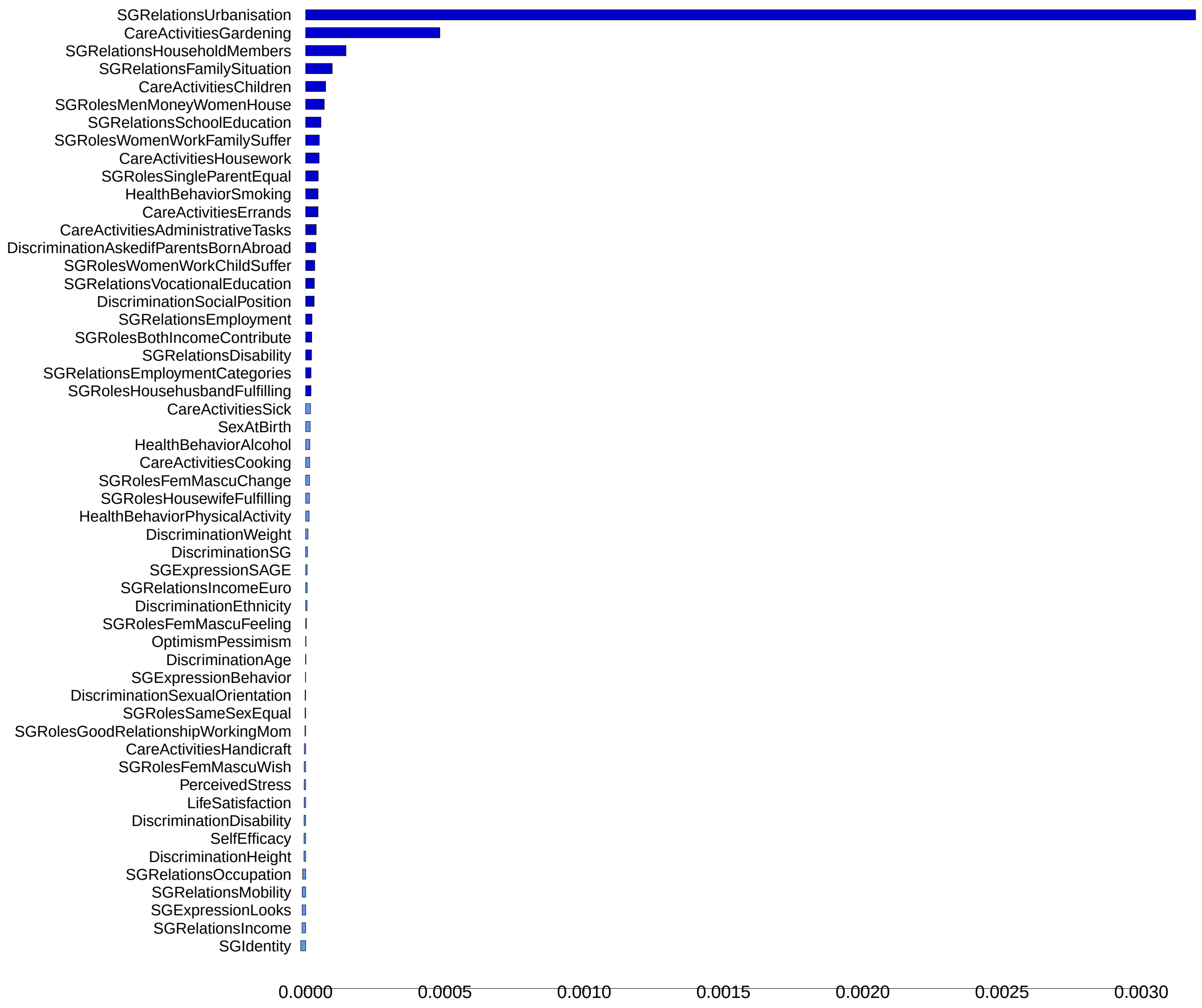

Supplement: Supplementary file 1 [file ijerph-19-07476-s001.zip › ijerph-1725340-supplementary/Supplementary Materials/Supplementary Materials S2_ Results_53Covariates.pdf]
