# Supplementary material for: Decision Tree Analyses to Explore the Relevance of Multiple Sex/Gender Dimensions for the Exposure to Green Spaces: Results from the KORA INGER Study
Source: Int J Environ Res Public Health. 2022 Jun 18;19(12):7476. doi: 10.3390/ijerph19127476 (PMC9224469; doi:10.3390/ijerph19127476)
Supplement: Supplementary file 1 [file ijerph-19-07476-s001.zip › ijerph-1725340-supplementary/Supplementary Materials/Supplementary Materials S3_Supplementary_Tables_S1_S3.pdf]

**Supplementary Table S1: The 53 Sex/gender covariates used in the analyses within the INGER KORA FIT sample**

| Covariate name and question in INGER KORA survey or KORA FIT survey (Original questions were asked in German) | Answer categories and distribution in the INGER KORA FIT sample; *N* = 2624 (100 %) | |
|---|---|---|
| <u>**Individual sex/gender self-concept**</u> | | |
| **Sex assigned at birth** | | |
| | 1426 (54.3) | = female |
| **SexAtBirth** | 1177 (44.9) | = male |
| What sex were you assigned at birth? | 0 (0.0) | = diverse / intersexual |
| | 21 (0.8) | = missing |
| **Current sex/gender identity** | | |
| | 1415 (53.9) | = female |
| **SGIdentity** | 1162 (44.3) | = male |
| What is your current sex/gender identity? | 0 (0.0) | = intersexual |
| (Multiple answers possible) | 1 (0.04) | = trans, trans man, trans woman |
| | 2 (0.1) | = an identity not mentioned here |
| | 7 (0.3) | = I do not want to classify as any sex/gender category |
| | 2 (0.1) | = female AND I do not want to classify as any sex/gender category |
| | 2 (0.1) | = male AND an identity not mentioned here |
| | 3 (0.1) | = male AND I do not want to classify as any sex/gender category |
| | 30 (1.1) | = missing |
| **Internalized sex/gender roles** | | |
| | 295 (11.2) | = very masculine |
| **SGRolesFemMascuFeeling** | 602 (22.9) | = mainly masculine |
| I mostly perceive myself as … | 214 (8.2) | = a little masculine |
| | 320 (12.2) | = just as feminine as masculine |
| | 123 (4.7) | = a little feminine |
| | 681 (26.0) | = mainly feminine |
| | 294 (11.2) | = very feminine |
| | 95 (3.6) | = missing |
| | 352 (13.4) | = very masculine |
| **SGRolesFemMascuWish** | 595 (22.7) | = mainly masculine |
| Ideally, I would like to be ... | 138 (5.3) | = a little masculine |
| | 288 (11.0) | = just as feminine as masculine |
| | 112 (4.3) | = a little feminine |
| | 623 (23.7) | = mainly feminine |
| | 377 (14.4) | = very feminine |
| | 139 (5.3) | = missing |
| | 263 (10.0) | = yes |
| **SGRolesFemMascuChange** | 2278 (86.8) | = no |
| Has your assessment of what is feminine or masculine changed in recent years? | 83 (3.2) | = missing |
| | 1229 (46.8) | = strongly agree |
| **SGRolesBothIncomeContribute** | 722 (27.5) | = rather agree |

| | | |
|---|---|---|
| Both the men and women should contribute to the household income. | 558 (21.3) | = neither agree nor disagree |
| | 67 (2.6) | = rather disagree |
| | 20 (0.8) | = strongly disagree |
| | 28 (1.1) | = missing |
| | 47 (1.8) | = strongly agree |
| **SGRolesMenMoneyWomenHouse** | 193 (7.4) | = rather agree |
| The man's job is to earn money; a woman's job is to look after the home and family. | 608 (23.2) | = neither agree nor disagree |
| | 752 (28.7) | = rather disagree |
| | 998 (38.0) | = strongly disagree |
| | 26 (1.0) | = missing |
| | 1296 (49.4) | = strongly agree |
| **SGRolesGoodRelationshipWorkingMom** | 694 (26.5) | = rather agree |
| A working mother can establish just as warm and secure a relationship with her children as a mother who does not work. | 431 (16.4) | = neither agree nor disagree |
| | 147 (5.6) | = rather disagree |
| | 34 (1.3) | = strongly disagree |
| | 22 (0.8) | = missing |
| | 255 (9.7) | = strongly agree |
| **SGRolesWomenWorkChildSuffer** | 704 (26.8) | = rather agree |
| A pre-school child is likely to suffer if his or her mother works. | 797 (30.4) | = neither agree nor disagree |
| | 550 (21.0) | = rather disagree |
| | 293 (11.2) | = strongly disagree |
| | 25 (1.0) | = missing |
| | 90 (3.4) | = strongly agree |
| **SGRolesWomenWorkFamilySuffer** | 374 (14.3) | = rather agree |
| All in all, family life suffers when the woman is working. | 971 (37.0) | = neither agree nor disagree |
| | 745 (28.4) | = rather disagree |
| | 422 (16.1) | = strongly disagree |
| | 22 (0.8) | = missing |
| | 349 (13.3) | = strongly agree |
| **SGRolesHousewifeFulfilling** | 441 (16.8) | = rather agree |
| Being a housewife is just as fulfilling as working for pay. | 791 (30.1) | = neither agree nor disagree |
| | 721 (27.5) | = rather disagree |
| | 292 (11.1) | = strongly disagree |
| | 30 (1.1) | = missing |
| | 218 (8.3) | = strongly agree |
| **SGRolesHousehusbandFulfilling** | 334 (12.7) | = rather agree |
| Being a househusband is just as fulfilling as working for pay. | 751 (28.6) | = neither agree nor disagree |
| | 866 (33.0) | = rather disagree |
| | 389 (14.8) | = strongly disagree |
| | 66 (2.5) | = missing |
| | 261 (10.0) | = strongly agree |
| **SGRolesSingleParentEqual** | 432 (16.5) | = rather agree |
| One parent can raise a child as well as two parents together. | 846 (32.2) | = neither agree nor disagree |
| | 823 (31.4) | = rather disagree |
| | 242 (9.2) | = strongly disagree |
| | 20 (0.8) | = missing |
| | 446 (17.0) | = strongly agree |
| **SGRolesSameSexEqual** | 603 (23.0) | = rather agree |
| A same-sex couple can raise a child as well as a male-female couple. | 645 (24.6) | = neither agree nor disagree |
| | 506 (19.3) | = rather disagree |
| | 399 (15.2) | = strongly disagree |
| | 25 (1.0) | = missing |

| | | |
|---|---|---|
| **Externalized sex/gender Expressions** | | |
| | 301 (11.5) | = very masculine |
| **SGExpressionLooks** | 621 (23.7) | = mainly masculine |
| How would other people generally | 177 (6.8) | = a little masculine |
| describe you based on your appearance, | 244 (9.3) | = just as feminine as masculine |
| clothing style and other visual | 194 (7.4) | = a little feminine |
| characteristics? | 714 (27.2) | = mainly feminine |
| | 254 (9.7) | = very feminine |
| | 119 (4.5) | = missing |
| | 280 (10.7) | = very masculine |
| **SGExpressionBehavior** | 613 (23.4) | = mainly masculine |
| How would other people generally | 210 (8.0) | = a little masculine |
| describe you based on your behaviors? | 341 (13.0) | = just as feminine as masculine |
| | 163 (6.2) | = a little feminine |
| | 671 (25.6) | = mainly feminine |
| | 228 (8.7) | = very feminine |
| | 118 (4.5) | = missing |
| | 611 (23.3) | = 1.0 – 1.5 |
| **SGExpressionSAGE** | 1245 (47.5) | = 2.0 – 2.5 |
| Combination of SexAtBirth, | 366 (14.0) | = 3.0 – 3.5 |
| SGExpressionLooks and | 227 (8.7) | = 4.0 – 4.5 |
| SGExpressionBehavior | 33 (1.3) | = 5.0 – 5.5 |
| SAGE-Score values: 1.0 – 7.0 | 4 (0.2) | = 6.0 – 6.5 |
| low values = high socially assigned gender conformity | 4 (0.1) | = 7.0 |
| high values = low socially assigned gender conformity | 135 (5.1) | = missing |
| **<u>Items contributing to explain structural sex/gender relations</u>** | | |
| **Experience of discrimination** | | |
| | 21 (0.8) | = strongly agree |
| **DiscriminationSocialPosition** | 133 (5.1) | = rather agree |
| | 228 (8.7) | = neither agree nor disagree |
| I have the feeling to be disadvantaged | 1004 (38.3) | = rather disagree |
| because of my position in society. | 1212 (46.2) | = strongly disagree |
| | 26 (1.0) | = missing |
| | 23 (0.9) | = strongly agree |
| **DiscriminationAge** | 172 (6.6) | = rather agree |
| I have the feeling to be disadvantaged | 292 (11.1) | = neither agree nor disagree |
| because of my age. | 983 (37.5) | = rather disagree |
| | 1133 (43.2) | = strongly disagree |
| | 21 (0.8) | = missing |
| | 12 (0.5) | = strongly agree |
| **DiscriminationHeight** | 51 (1.9) | = rather agree |
| I have the feeling to be disadvantaged | 102 (3.9) | = neither agree nor disagree |
| because of my height. | 662 (25.2) | = rather disagree |
| | 1780 (67.8) | = strongly disagree |
| | 17 (0.7) | = missing |
| | 17 (0.7) | = strongly agree |
| **DiscriminationWeight** | 51 (1.9) | = rather agree |
| | 128 (4.9) | = neither agree nor disagree |

| | | |
|---|---|---|
| I have the feeling to be disadvantaged because of my weight. | 644 (24.5) | = rather disagree |
| | 1767 (67.3) | = strongly disagree |
| | 17 (0.7) | = missing |
| **DiscriminationDisability** | 24 (0.9) | = strongly agree |
| I have the feeling to be disadvantaged because of my physical impairment. | 66 (2.5) | = rather agree |
| | 148 (5.6) | = neither agree nor disagree |
| | 457 (17.4) | = rather disagree |
| | 1903 (72.5) | = strongly disagree |
| | 26 (1.0) | = missing |
| **DiscriminationEthnicity** | 7 (0.3) | = strongly agree |
| I have the feeling to be disadvantaged because of my ethnic/cultural affiliation. | 16 (0.6) | = rather agree |
| | 40 (1.5) | = neither agree nor disagree |
| | 279 (10.6) | = rather disagree |
| | 2254 (85.9) | = strongly disagree |
| | 28 (1.1) | = missing |
| **DiscriminationSG** | 10 (0.4) | = strongly agree |
| I have the feeling to be disadvantaged because of my sex/gender. | 24 (0.9) | = rather agree |
| | 88 (3.4) | = neither agree nor disagree |
| | 340 (13.0) | = rather disagree |
| | 2143 (81.7) | = strongly disagree |
| | 19 (0.7) | = missing |
| **DiscriminationSexualOrientation** | 7 (0.3) | = strongly agree |
| I have the feeling to be disadvantaged because of my sexual orientation. | 2 (0.1) | = rather agree |
| | 13 (0.5) | = neither agree nor disagree |
| | 163 (6.2) | = rather disagree |
| | 2393 (91.2) | = strongly disagree |
| | 46 (1.8) | = missing |
| **DiscriminationAskedifParentsBornAbroad** | 289 (11.0) | = yes |
| Have you ever been asked in Germany whether you or your parents were born abroad? | 2307 (87.9) | = no |
| | 28 (1.1) | = missing |
| **Care activities** | | |
| **CareActivitiesChildren** | 93 (3.5) | = only me |
| Who is currently taking primary responsibility for the following tasks? ("Other people" also includes your partner) | 205 (7.8) | = mainly me |
| | 857 (32.7) | = me and other people |
| | 313 (11.9) | = mainly other people |
| | 46 (1.8) | = only other people |
| | 1090 (41.5) | = not applicable |
| | 20 (0.8) | = missing |
| Care and/or upbringing of your children/grandchildren, driving services for your children/grandchildren | | |
| **CareActivitiesSick** | 115 (4.4) | = only me |
| Who is currently taking primary responsibility for the following tasks? ("Other people" also includes your partner) | 203 (7.7) | = mainly me |
| | 425 (16.2) | = me and other people |
| | 169 (6.4) | = mainly other people |
| | 50 (1.9) | = only other people |
| | 1632 (62.2) | = not applicable |
| | 30 (1.1) | = missing |

| | | |
|---|---|---|
| Care for disabled, chronically ill or in need of care family members, neighbors or friends | | |
| **CareActivitiesCooking**<br>Who is currently taking primary responsibility for the following tasks? ("Other people" also includes your partner)<br><br>Cooking | 739 (28.2)<br>518 (19.7)<br>638 (24.3)<br>467 (17.8)<br>228 (8.7)<br>34 (1.3) | = only me<br>= mainly me<br>= me and other people<br>= mainly other people<br>= only other people<br>= missing |
| **CareActivitiesHousework**<br>Who is currently taking primary responsibility for the following tasks? ("Other people" also includes your partner)<br><br>Housework | 627 (23.9)<br>606 (23.1)<br>871 (33.2)<br>437 (16.7)<br>59 (2.3)<br>24 (0.9) | = only me<br>= mainly me<br>= me and other people<br>= mainly other people<br>= only other people<br>= missing |
| **CareActivitiesGardening**<br>Who is currently taking primary responsibility for the following tasks? ("Other people" also includes your partner)<br><br>Gardening (during the gardening season) | 334 (12.7)<br>499 (19.0)<br>1051 (40.1)<br>282 (11.3)<br>63 (2.4)<br>371 (14.1)<br>24 (0.9) | = only me<br>= mainly me<br>= me and other people<br>= mainly other people<br>= only other people<br>= not applicable<br>= missing |
| **CareActivitiesErrands**<br>Who is currently taking primary responsibility for the following tasks? ("Other people" also includes your partner)<br><br>Errands (shopping, procurement) | 547 (20.9)<br>549 (20.9)<br>1137 (43.3)<br>313 (11.9)<br>36 (1.4)<br>42 (1.6) | = only me<br>= mainly me<br>= me and other people<br>= mainly other people<br>= only other people<br>= missing |
| **CareActivitiesAdministrativeTasks**<br>Who is currently taking primary responsibility for the following tasks? ("Other people" also includes your partner)<br><br>Administrative tasks (insurance, tax return, etc.) | 712 (27.1)<br>597 (22.8)<br>847 (32.3)<br>357 (13.6)<br>84 (3.2)<br>27 (1.0) | = only me<br>= mainly me<br>= me and other people<br>= mainly other people<br>= only other people<br>= missing |
| **CareActivitiesHandicraft**<br>Who is currently taking primary responsibility for the following tasks? ("Other people" also includes your partner) | 483 (18.4)<br>686 (26.1)<br>662 (25.2)<br>578 (22.0)<br>192 (7.3)<br>23 (0.9) | = only me<br>= mainly me<br>= me and other people<br>= mainly other people<br>= only other people<br>= missing |

| | | |
|---|---|---|
| Handicraft tasks in the household | | |
| **Intersectionality-related social categories** | | |
| | 1224 (46.7) | = Degree after German basic secondary school (Hauptschulabschluss) |
| **SGRelationsSchoolEducation** | | |
| School education | 767 (29.2) | = German O-levels (Mittlere Reife) |
| *(Variable from basic KORA studies S1-S4)* | 633 (24.1) | = German A-Levels (Abitur) |
| | 0 (0.0) | = missing |
| | 166 (6.3) | = no vocational qualification |
| **SGRelationsVocationalEducation** | 1464 (55.8) | = vocational school / apprenticeship |
| Highest vocational qualification | 503 (19.2) | = technical school / master school |
| *(Variable from basic KORA studies S1-S4)* | 21(0.8) | = engineering school / polytechnical school |
| | 470 (17.9) | = university of applied sciences / university |
| | 0 (0.0) | = missing |
| | 1466 (55.8) | = no |
| **SGRelationsEmployment** | 21 (0.8) | = 1-5 h/week |
| Are you employed? | 109 (4.2) | = 6-10 h/week |
| If so: How many hours do you work on | 158 (6.0) | = 11-20 h/week |
| average per week (actual working hours)? | 156 (6.0) | = 21-30 h/week |
| | 407 (15.5) | = 31-40 h/week |
| | 191 (7.3) | = 41-50 h/week |
| | 60 (2.3) | = > 50 h/week |
| | 56 (2.1) | = missing |
| | 1278 (48.7) | = a pensioner |
| **SGRelationsEmploymentCategories** | 102 (3.9) | = in partial retirement |
| Which of the following statements applies | 1026 (39.1) | = working |
| to you? | 40 (1.5) | = unemployed |
| Are you currently ... | 177 (6.7) | = other status *(e.g. housewife/ househusband, in training, other leave of absence)* |
| | 1 (0.04) | = missing |
| | 52 (2.0) | = unskilled worker |
| **SGRelationsOccupation** | 115 (4.4) | = Civil servant simple activity / semi-skilled worker |
| What position do you have, or did you have in your job? | 396 (15.1) | = Civil servant simple service / skilled worker |
| + one of four more detailed follow-up questions depending on the initial answer (worker, employee, civil-servant or self-employed) | 133 (5.1) | = Civil servant middle service / skilled worker or foreman |
| | 43 (1.6) | = Master craftsman / foreman |
| | 1040 (39.6) | = Civil servant higher service / employee with qualified activity |
| | 162 (6.2) | = Self-employed, max. 1 employee |
| | 541 (20.6) | = Civil servant higher service / employee with highly qualified activity / self-employed academic / self-employed with max. 9 employees |
| | 79 (3.0) | = Employees with extensive managerial responsibilities / self-employed with at least 10 employees |
| | 63 (2.4) | = missing |
| | 289 (11.0) | = very good |

| | | |
|---|---|---|
| **SGRelationsIncome** | 1638 (62.4) | = good |
| How do you assess your financial situation? | 620 (23.6) | = moderate |
| | 49 (1.9) | = bad |
| | 28 (1.1) | = missing |
| **SGRelationsIncomeEuro** | Continuous | variable (€) |
| Equivalent income = the household's income divided by the number of consumption units in the household. | 1902.00 | = mean |
| | 923.39 | = SD |
| | 185.00 | = min |
| (To calculate this, participants were asked about their household income and number of households' members and the age of the members) | 8000.00 | = max |
| | 195 | = missing |
| **SGRelationsFamilySituation** | 2076 (79.1) | = yes |
| Do you live with a spouse or partner in a shared household? | 548 (20.9) | = no |
| | 0 (0.0) | = missing |
| | Continuous | variable |
| **SGRelationsHouseholdMembers** | 2.08 | = mean |
| How many people live in your household at all times, including yourself? | 0.83 | = SD |
| | 1.00 | = min |
| | 9.00 | = max |
| | 2 | = missing |
| **SGRelationsDisability** | 2112 (80.5) | = no disability |
| Do you have a recognized disability? | 141 (5.4) | = disability 10 - 30% |
| If yes, what degree of disability do you have? | 280 (10.7) | = disability 40 - 60% |
| | 89 (3.4) | = disability 70-100% |
| | 2 (0.1) | = missing |
| | 1892 (72.1) | = I have no problems moving around |
| **SGRelationsMobility** | 451 (17.2) | = I have little problems moving around |
| Please indicate which of the following statements best describes your current state of health regarding mobility. | 206 (7.9) | = I have moderate problems moving around |
| | 74 (2.8) | = I have big problems moving around / I am unable to move |
| | 1 (0.04) | = missing |
| **SGRelationsUrbanisation** | 945 (36.0) | = city |
| Distribution of participants by degree of urbanisation. | 1080 (41.2) | = suburb |
| | 589 (22.5) | = rural |
| | 10 (0.4) | = missing |
| **Life-style and psychological factors** | | |
| **Health Behaviors** | | |
| | Continuous | variable |
| **HealthBehaviorAlcohol** | 14.72 | = mean |
| Alcohol consumption (g /day) | 19.55 | = SD |
| | 0.00 | = min |
| (To calculate this, participants were asked | 179.40 | = max |
| about their consumption of different kind | 1 | = missing |

| | | |
|---|---|---|
| of alcoholic drinks on weekends or working days) | | |
| **HealthBehaviorSmoking**<br>Smoking behavior based on three interview questions<br>a) Do you currently smoke cigarettes?<br>b) Do you smoke regularly or irregularly (i.e. usually less than one cigarette a day)?<br>c) Have you ever smoked cigarettes? | 303 (11.5)<br>43 (1.6)<br>1085 (41.3)<br>1191 (45.4)<br>2 (0.1) | = regular smoker<br>= irregular smoker<br>= ex-smoker<br>= never-smoker<br>= missing |
| **HealthBehaviorPhysicalActivity**<br>How often do you do sports in winter/summer? | 1021 (38.9)<br>889 (33.9)<br>322 (12.3)<br>392 (14.9)<br>0 (0.0) | = regularly > 2h a week<br>= regularly ~ 1h a week<br>= irregularly ~ 1h a week<br>= almost no or no physical activity<br>= missing |
| **Psychosocial Factors** | | |
| **LifeSatisfaction**<br>Overall, how satisfied are you with your life right now?<br>on a scale from 0 (totally dissatisfied) – 10 (completely satisfied) | Continuous<br>7.47<br>2.25<br>0.00<br>10.00<br>56 | variable<br>= mean<br>= SD<br>= min<br>= max<br>= missing |
| **PerceivedStress**<br>Based on 10 questions of the German version of the Perceived Stress Scale (see Klein [58] for exact formulation of questions)<br>Scores are obtained by reversing responses to four positively stated items and then summing across all scale items.<br>Higher scores indicated a higher level of perceived stress. | Continuous<br>14.28<br>5.56<br>0.00<br>34.00<br>233 | variable<br>= mean<br>= SD<br>= min<br>= max<br>= missing |
| **SelfEfficacy**<br>Self-efficacy scale (ASKU, see Beierlein[59])<br>Score is based on three statements on a scale from 1 (strongly disagree) – 5 (strongly agree):<br>a) I can rely on my own abilities in difficult situations.<br>b) I am able to solve most problems on my own.<br>c) I can usually solve even challenging and complex tasks well. | Continuous<br>4.02<br>0.58<br>1.00<br>5.00<br>76 | variable<br>= mean<br>= SD<br>= min<br>= max<br>= missing |

| | | |
|---|---|---|
| Score is the average answer to these three statements. | | |
| **OptimismPessimism** | Continuous | variable |
| Based on questions of the German version of the Life-Orientation-Test (LOT-R) for dispositional optimism and pessimism (see Glaesmer[60]) | 7.62 | = mean |
| | 3.59 | = SD |
| | 0.00 | = min |
| | 22.00 | = max |
| | 206 | = missing |
| Scores are obtained by reversing responses to positively stated items and then summing across all scale items. | | |
| Higher scores indicated a higher level of pessimism. | | |


58. Klein E.M.; Brahler E.; Dreier M.; Reinecke L.; Muller K.W.; Schmutzer G.; Wolfling K.; Beutel M.E. The German version of the Perceived Stress Scale - psychometric characteristics in a representative German community sample. BMC Psychiatry 2016, 16, 159. doi:10.1186/s12888-016-0875-9

59. Beierlein C.; Kovaleva A.; Kemper C.J.; Rammstedt B. ASKU - Allgemeine Selbstwirksamkeit Kurzskala [Fragebogen]. Elektronisches Testarchiv (PSYNDEX Tests-Nr. 9006490). Trier: ZPID.: Leibniz-Zentrum für Psychologische Information und Dokumentation (ZPID); 2012.

60. Glaesmer H.; Hoyer J.; Klotsche J.; Herzberg P.Y. The German version of the Life-Orientation-Test (LOT-R) for dispositional optimism and pessimism. Z Gesundh 2008, 16(1), 26-31. doi:10.1026/0943-8149.16.1.26

**Supplementary Table S2: Exposure distribution within the INGER KORA FIT sample**

| Exposure and, if applicable, questions in INGER KORA survey (Original questions were asked in German) | Answer categories and distribution in the whole INGER study sample; *N* = 2624 (100 %) | |
|---|---|---|
| **Subjective exposure measurement** | | |
| **Access to public green spaces** | 2382 (90.8) | = yes |
| Are there publicly accessible green spaces (e.g. parks, forests, meadows) in your neighbourhood? | 228 (8.7) | = no |
| | 14 (0.5) | = missing |
| **Access to high quality public green spaces** | 737 (28.1) | = high quality green |
| The green spaces in my neighbourhood are well maintained. | 1555 (59.3) | = only lower quality green |
| The green spaces in my neighbourhood are of high quality. | 228 (8.7) | = no green |
| *(Combination of three variables)* | 104 (4.0) | = missing |
| **Greenness in the neighbourhood** | 2074 (79.0) | = very green |
| How green is your neighbourhood? | 480 (18.3) | = little green |
| (From green strips along the road to gardens and parks.) | 54 (2.1) | = hardly green |
| | 16 (0.6) | = missing |
| **Objective exposure measurement** | **Continuous variables** | |
| **Greenness within a 300 m buffer around the residential address** | 0.16 | = min |
| Calculated from several satellite images between April and October in 2019. Negative pixels of the NDVI map were excluded prior to assignment to home addresses. | 0.41 | = Q1 |
| | 0.47 | = median |
| | 0.47 | = mean |
| | 0.09 | = SD |
| | 0.53 | = Q3 |
| | 0.73 | = max |
| | 10 | = missing |
| **Greenness within a 1000 m buffer around the residential address** | 0.27 | = min |
| Calculated from several satellite images between April and October in 2019. Negative pixels of the NDVI map were excluded prior to assignment to home addresses. | 0.44 | = Q1 |
| | 0.51 | = median |
| | 0.50 | = mean |
| | 0.09 | = SD |
| | 0.58 | = Q3 |
| | 0.71 | = max |
| | 10 | = missing |

**Supplementary Table S3: Further description of the INGER KORA FIT sample**

| Question in INGER KORA survey<br>(Original questions were asked in German) | Answer categories and distribution in the INGER KORA FIT sample;<br>*N* = 2624 (100 %) | |
|---|---|---|
| | | |
| Age distribution in the INGER FIT study population. | Continuous variable | (years) |
| | 63.58 | = mean |
| | 5.42 | = SD |
| | 54.00 | = min |
| | 73.00 | = max |
| | 0 | = missing |
| What is your current employment status? | 1102 (42.0) | = employed |
| | 1332 (50.8) | = retired |
| | 134 (5.1) | = other |
| | 56 (2.1) | = missing |
| Distribution of participants by degree of urbanisation. | 945 (36.0) | = city |
| | 1080 (41.2) | = suburb |
| | 589 (22.5) | = rural |
| | 10 (0.4) | = missing |
| Do you live…? | 2089 (79.6) | = in your own property |
| | 515 (19.6) | = for rent |
| | 20 (0.8) | = missing |
| How long have you lived at your current address? | Continuous variable | (years) |
| | 29 | = mean |
| | 15.34 | = SD |
| | 0 | = min |
| | 74 | = max |
| | 43 | = missing |
| How often do you usually reside at your current address? | 2562 (97.6) | = daily |
| | 15 (0.6) | = only on weekdays |
| | 16 (0.5) | = only on days off |
| | 12 (0.7) | = few days a month |
| | 19 (0.0) | = missing |
| Does your flat or house have a garden? | 1841 (70.2) | = yes, for sole use |
| | 236 (9.0) | = yes, shared with several parties |
| | 520 (19.8) | = no |
| | 27 (1.0) | = missing |
| Do you have a balcony and/or roof terrace? | 1895 (72.2) | = yes |
| | 677 (25.8) | = no |
| | 52 (2.0) | = missing |
| Do you use your garden, balcony or roof terrace for recreation? | 2363 (90.1) | = yes |
| | 194 (7.4) | = no |
| | 48 (1.8) | = neither garden, balcony nor roof terrace available |
| | 19 (0.7) | = missing |
| During the summer months, how often do you visit publicly accessible green spaces, such as… | 189 (7.2) | = (almost) never |
| | 209 (8.0) | = 3-6 times per year |
| | 185 (7.1) | = 7-10 times per year |

| | | |
|---|---|---|
| … parks, forests, meadows, which you can reach in about 10 minutes? | 367 (14.0) | = at least once a month |
| | 829 (31.6) | = at least once a week |
| | 552 (21.0) | = (almost) daily |
| | 228 (8.7) | = no publicly accessible green spaces available |
| | 65 (2.5) | = missing |
| During the summer months, how often do you visit publicly accessible green spaces, such as… | 363 (13.8) | = (almost) never |
| | 383 (14.6) | = 3-6 times per year |
| | 339 (12.9) | = 7-10 times per year |
| … parks, forests, meadows, which you cannot reach in about 10 minutes? | 581 (22.1) | = at least once a month |
| | 512 (19.5) | = at least once a week |
| | 88 (3.4) | = (almost) daily |
| | 228 (8.7) | = no publicly accessible green spaces available |
| | 130 (5.0) | = missing |
